# Renin-angiotensin system inhibitor discontinuation in COVID-19 did not modify systemic ACE2 in a randomized controlled trial

**DOI:** 10.1016/j.isci.2023.108146

**Published:** 2023-10-05

**Authors:** Vincent Rathkolb, Marianna T. Traugott, Andreas Heinzel, Marko Poglitsch, Judith Aberle, Farsad Eskandary, Agnes Abrahamowicz, Martin Mueller, Petra Knollmueller, Tarik Shoumariyeh, Jasmin Stuflesser, Ivan Seeber, Georg Gibas, Hannah Mayfurth, Viktoria Tinhof, Lukas Schmoelz, Markus Zeitlinger, Christian Schoergenhofer, Bernd Jilma, Bernd Genser, Wolfgang Hoepler, Sara Omid, Mario Karolyi, Christoph Wenisch, Rainer Oberbauer, Alexander Zoufaly, Manfred Hecking, Roman Reindl-Schwaighofer

**Affiliations:** 1 Department of Clinical Pharmacology, Medical University of Vienna, Wien, Austria; 2 Department of Internal Medicine IV with Infectious Diseases and Tropical Medicine, Clinic Favoriten, Vienna, Austria; 3 Department of Internal Medicine III, Clinical Division of Nephrology & Dialysis, Medical University of Vienna, Wien, Austria; 4 Institute of Virology, Medical University of Vienna, Wien, Austria; 5 Attoquant Diagnostics, Vienna, Austria; 6 Center for Preventive Medicine and Digital Health, Medical Faculty Mannheim, Heidelberg University, Heidelberg, Germany; 7 Faculty of Medicine, Sigmund Freud University, Vienna, Austria

**Keywords:** Virology, Human metabolism

## Abstract

Despite the similar clinical outcomes after renin-angiotensin system (RAS) inhibitor (RASi) continuation or withdrawal in COVID-19, the effects on angiotensin-converting enzyme 2 (ACE2) and RAS metabolites remain unclear. In a substudy of the randomized controlled Austrian Corona Virus Adaptive Clinical Trial (ACOVACT), patients with hypertension and COVID-19 were randomized 1:1 to either RASi continuation (n = 30) or switch to a non-RASi medication (n = 29). RAS metabolites were analyzed using a mixed linear regression model (n = 30). Time to a sustained clinical improvement was equal and ACE2 did not differ between the groups but increased over time in both. Overall ACE2 was higher with severe COVID-19. ACE-S and Ang II levels increased as expected with ACE inhibitor discontinuation. These data support the safety of RASi continuation in COVID-19, although RASi were frequently discontinued in our post hoc analysis. The study was not powered to draw definite conclusions on clinical outcomes using small sample sizes.

## Highlights

• ACE2 levels were not different between RASi continuation and discontinuation
• Changes in RAS profiles were consistent with the mode of action of ACEi and ARBs
• ACE2 increases with severe disease progression in COVID-19
• Unreasonable RAS inhibitor discontinuation appears to occur frequently in COVID-19

## Graphical abstract

**Figure:**
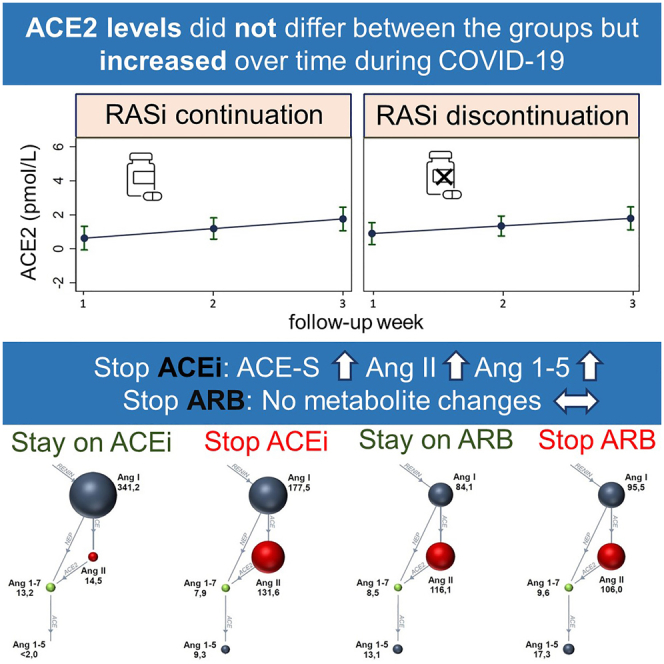

## Introduction

**Figure 1:**
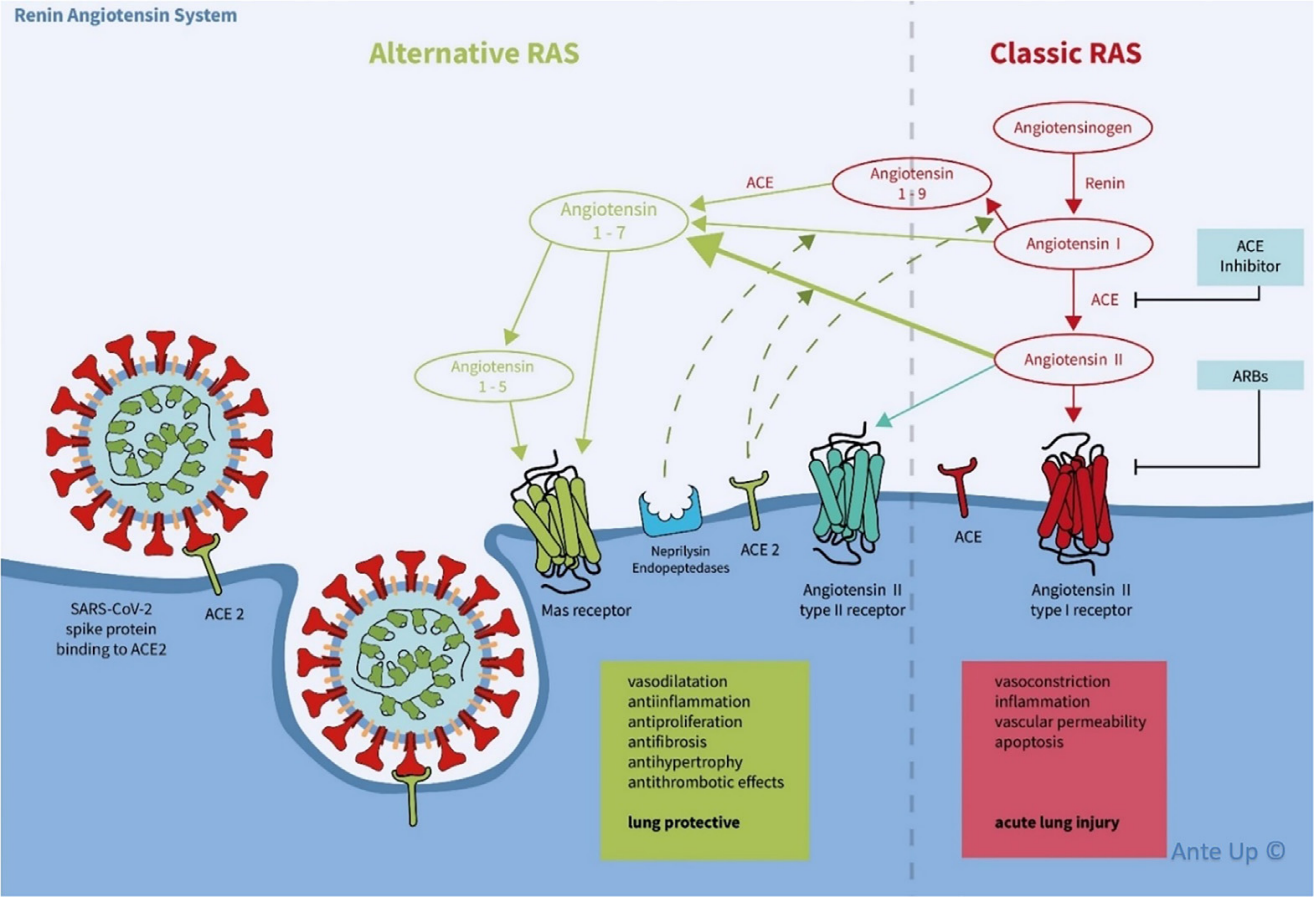
Schema of the classic (in red) and alternative (in green) renin-angiotensin systems and the cell entrance mechanism of SARS-CoV-2 using the membrane-bound ACE2 receptor ACE, angiotensin-converting enzyme; Ang, angiotensin; ARB, angiotensin receptor blocker; RAS, renin-angiotensin system; SARS-CoV-2, severe acute respiratory syndrome coronavirus 2.

Arterial hypertension, kidney disease, diabetes mellitus, and obesity are among the most important risk factors for severe coronavirus disease 2019 (COVID-19).^1^ The renin-angiotensin system (RAS) plays an important role in the pathophysiology of all these diseases, and RAS inhibitory medication is frequently prescribed.^2^^,^^3^^,^^4^ The severe acute respiratory syndrome coronavirus 2 (SARS-CoV-2) uses membrane-bound angiotensin-converting enzyme (ACE) 2 as its primary entry receptor linking RAS and COVID-19.^5^^,^^6^ ACE2 is a type I integral membrane glycoprotein that primarily metabolizes circulating angiotensin (Ang) II to Ang 1–7 and thereby represents the core enzyme of the “alternative” RAS (Figure 1).^7^

Ang 1–7 functions as an agonist of the Mas receptor and mediates vasodilatory, antiproliferative, antifibrotic, antihypertrophic, and antithrombotic effects.^8^^,^^9^ In this way, it antagonizes the effects of Ang II, the key metabolite of the classical RAS axis. Binding of Ang II to the angiotensin type 1 (AT1) receptor causes vasoconstriction, fibrosis, and pro-inflammatory reactions. A downregulation of ACE2 in the lungs of SARS-CoV-1 patients has been hypothesized to play a crucial role in the pathophysiology of SARS-related lung injury.^10^ Similar mechanisms have been postulated to cause acute respiratory distress syndrome (ARDS) in COVID-19,^11^^,^^12^ but systemic ACE2 increases markedly in COVID-19 patients with severe disease progression.^13^^,^^14^ ACE2 in serum is a biomarker of disease severity and associated with increased 30-day mortality.^15^ The classical RAS axis is highly activated in patients with severe COVID-19, noticeably driven by the effect of RAS inhibitor (RASi) therapies.^14^

Both ACE inhibitors (ACEis) and AT1 receptor blockers usually induce a shift toward the protective ACE2/Ang 1–7/Mas axis.^16^ Concerns about the safety of RASi medication in COVID-19 patients arose early in the pandemic based on the possibility that these drugs would facilitate viral infection through increased ACE2 expression in lung tissue.^17^^,^^18^^,^^19^^,^^20^ Epidemiological data, however, were neutral^21^^,^^22^^,^^23^^,^^24^ or even showed a beneficial effect of RASi medications.^25^^,^^26^^,^^27^ Several randomized controlled trials have since been conducted to assess the impact of RASi continuation or discontinuation on COVID-19 severity and found no difference in clinical outcomes.^28^^,^^29^^,^^30^

The impact of RASi continuation or discontinuation in COVID-19 on RAS metabolite and ACE2 levels has not been assessed to date. We hypothesized that stopping an ACEi or an angiotensin receptor blocker (ARB) in patients with COVID-19 would not affect ACE2 concentrations. In this randomized controlled trial on RASi continuation and discontinuation, we validated outcomes in patients hospitalized with COVID-19 and prospectively assessed changes in RAS metabolite profiles and ACE2 levels.

## Results

### Baseline characteristics

**Figure 2:**
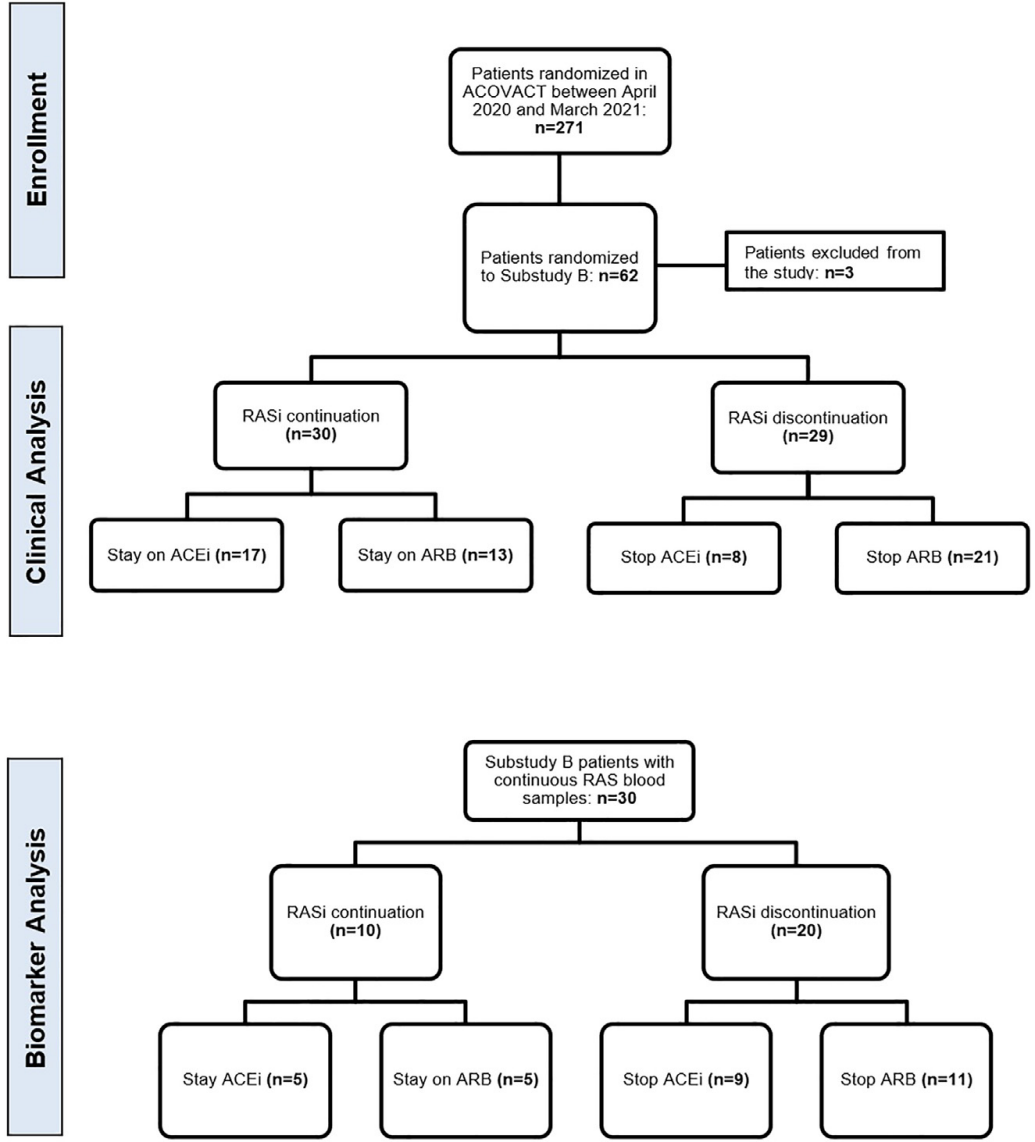
Consort Flow Chart ACEi, ACE inhibitor; ACOVACT, Austrian Corona Virus Adaptive Clinical Trial, ARB, Angiotensin receptor blocker; RAS, Renin-angiotensin system.

A total of 62 patients were enrolled in the substudy B of the ACOVACT trial, of whom 59 were assessed in an intention-to-treat analysis (30 in the RASi continuation group and 29 in discontinuation group; Figure 2). Three patients were excluded from the study: One patient tested positive for human immunodeficiency virus (HIV) who was also enrolled in the antiviral treatment study arm, whereas two patients were randomized to the RASi continuation group but not treated with RAS inhibitors. Baseline characteristics are provided in Table 1. The median age was 68 years in the RASi continuation group and 64 years in the RASi discontinuation group. Mean Charlson Comorbidity Index (CCI) score at baseline and median BMI were well-balanced between the groups (CCI: 3.5 ± 1.9 vs. 3.3 ± 1.9; BMI: 29.9 vs. 29.2 for RASi continuation vs. discontinuation, respectively; Table S1). In the RASi continuation group, 56.7% were on an ACEi and 43.3% on ARB treatment, whereas in the discontinuation group, 27.6% were on an ACEi and 72.4% on ARB at baseline. The median time interval from COVID-19 symptom onset to randomization was 7 days (RASi continuation) vs. 6 days (RASi discontinuation). The baseline WHO scores were 3.8 ± 0.7 in the RASi-continuation group and 3.8 ± 0.6 in the discontinuation group.

**Table 1:** Baseline characteristics and demographics in the clinical treatment and biomarker groups

Baseline characteristics^a^	Clinical treatment groups		Biomarker groups	
	RASi continuation (n = 30)	RASi discontinuation (n = 29)	RASi continuation (n = 10)	RASi discontinuation (n = 20)
**Demographics**				

Male	22 (73.3%)	15 (51.7%)	7 (70.0%)	12 (60.0%)
Female	8 (26.7%)	14 (48.3%)	3 (30.0%)	8 (40.0%)
Age (y)	67.5 (59.0–78.0)	64.0 (59.0–76.0)	68.0 (59.8–78.3)	62.0 (55.3–74.5)

**Comorbidities**				

Charlson Comorbidity Index score^b^	3.5 ± 1.9	3.3 ± 1.9	4.0 ± 2.3	3.3 ± 2.3
BMI (kg/m^2^)	29.9 (27.7–33.3)	29.2 (26.4–34.9)	32.9 (29.0–40.5)	29.3 (26.5–34.7)
Obesity	14 (46.7%)	12 (41.4%)	7 (70.0%)	9 (45.0%)
Chronic kidney disease (stage G2 to G3b)	4 (13.3%)	1 (3.4%)	0	2 (10.0%)
Diabetes mellitus	11 (36.7%)	11 (37.9%)	6 (60.0%)	6 (30.0%)
Arterial hypertension	30 (100.0%)	29 (100.0%)	10 (100.0%)	20 (100.0%)
Bronchial asthma	3 (10.0%)	2 (6.9%)	1 (10.0%)	2 (10.0%)
Hyperlipidemia	8 (26.7%)	10 (34.5%)	2 (20.0%)	6 (30.0%)
Coronary heart disease	4 (13.3%)	3 (10.3%)	2 (20.0%)	2 (10.0%)
Atrial fibrillation	3 (10.0%)	1 (3.4%)	2 (20.0%)	0

**RASi agents prior to admission**				

ACEi	17 (56.7%)	8 (27.6%)	5 (50.0%)	9 (45.0%)
ARB	13 (43.3%)	21 (72.4%)	5 (50.0%)	11 (55.0%)

**Blood pressure and laboratory values**				

Systolic blood pressure (mmHg)	130.0 (116.0–145.3)	127.0 (120.0–142.0)	130.0 (121.3–146.3)	130.0 (120.0–150.0)
Diastolic blood pressure (mmHg)	80.0 (70.0–87.0)	76.0 (70.0–80.5)	80.0 (70.0–86.3)	75.0 (70.0–84.0)
Potassium (mmol/L)	3.8 (3.6–4.1)	3.9 (3.5–4.2)	4.0 (3.6–4.1)	3.9 (3.8–4.2)
Sodium (mmol/L)	136.0 (132.0–139.3)	138.0 (134.0–139.5)	136.0 (130.8–138.3)	138.0 (134.0–139.0)
Creatinine (mg/dL)	0.9 (0.8–1.1)	0.9 (0.7–1.2)	0.9 (0.8–1.1)	1.1 (0.7–1.3)
Glomerular filtration rate (ml/min/1.7 m^2^)	80.8 (54.3–90.0)	82.4 (62.2–90.0)	74.1 (58.1–90.0)	78.8 (54.1–90.0)
CRP (mg/dL)	53.4 (20.1–75.9)	74.1 (35.3–106.6)	53.4 (39.0–63.3)	74.9 (40.0–117.0)
Leukocytes (G/L)	6.5 (5.2–8.2)	5.7 (4.3–8.5)	5.4 (5.0–7.0)	7.8 (5.3–10.1)
Neutrophils (G/L)	5.3 (3.9–6.9)	3.8 (3.2–7.0)	4.3 (3.5–6.0)	4.9 (3.6–7.9)
Lymphocytes (G/L)	0.8 (0.6–1.0)	0.8 (0.6–1.2)	0.7 (0.6–0.7)	0.9 (0.8–1.3)
Platelets (G/L)	185.5 (137.8–263.3)	183.5 (160.5–241.3)	159.5 (128.3–222.0)	184.0 (137.0–242.0)
Troponin T (ng/L)	16.2 (8.3–21.6)	13.1 (7.9–21.1)	15.7 (7.0–23.7)	12.0 (8.3–20.4)

**COVID-19**				

Symptom onset to hospital admission (days)	5.0 (4.0–8.5)	5.0 (3.0–7.5)	5.0 (4.0–10.0)	4.5 (3.0–7.3)
Symptom onset to randomization (days)	7.0 (4.5–10.5)	6.0 (4.0–8.0)	7.0 (5.0–12.0)	6.0 (4.0–8.0)
Baseline WHO score^b^	3.8 ± 0.7	3.8 ± 0.6	3.7 ± 0.8	3.8 ± 0.6
Baseline NEWS†	4.9 ± 2.3	3.9 ± 2.3	5.1 ± 3.3	4.8 ± 2.3

ACEi, ACE inhibitor; ARB, angiotensin receptor blocker; BMI, body mass index; COVID-19, coronavirus disease 2019; CRP, C-reactive protein; NEWS, National Early Warning Score; RAS, renin-angiotensin system; RASi, renin-angiotensin system inhibitor; WHO, World Health Organization.

a Continuous variables are presented as medians (IQRs), binary variables are presented in absolute numbers (%).

b Data are presented as means (SDs).

### Clinical outcomes

#### Primary endpoint

**Figure 3:**
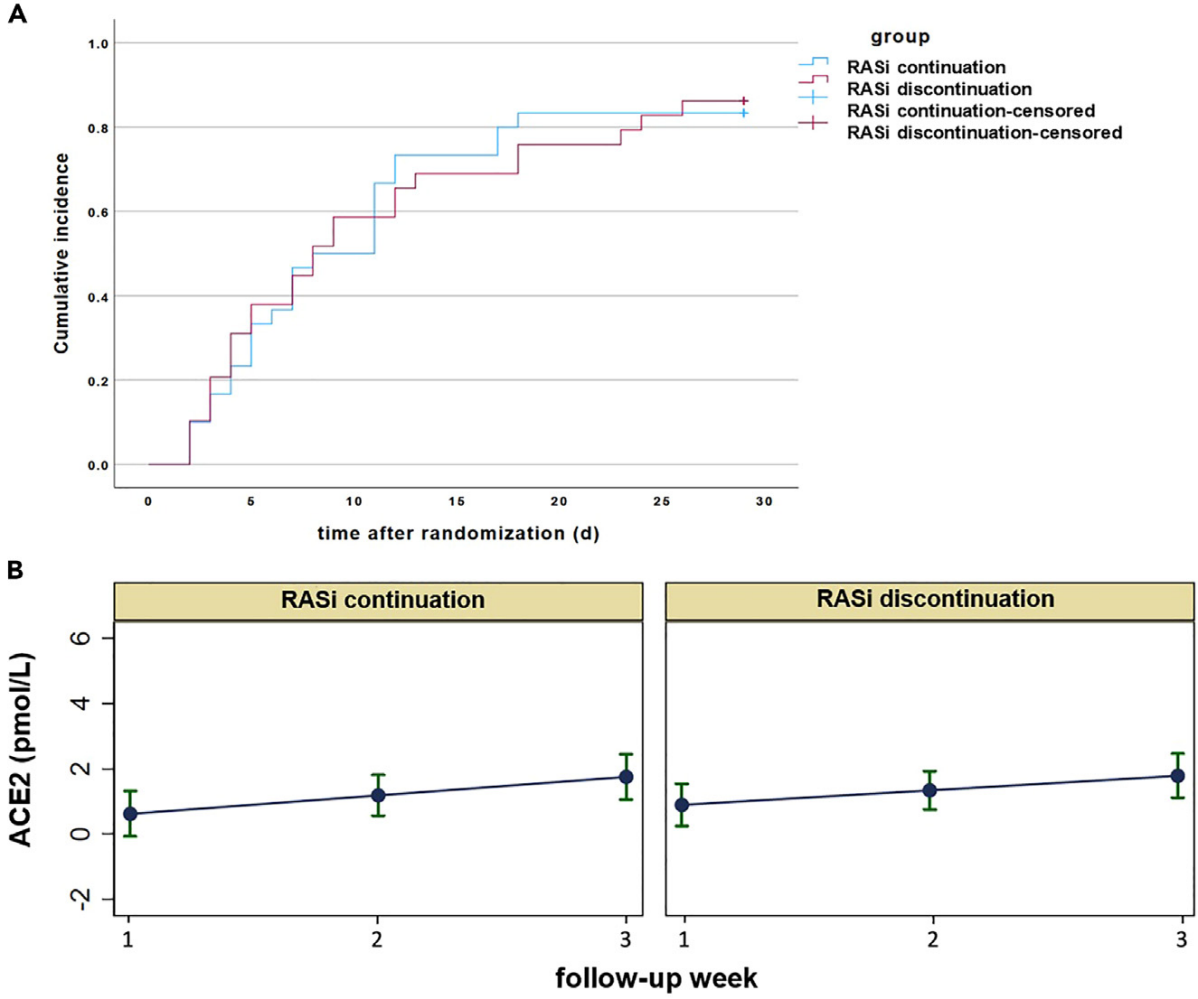
WHO score improvement from baseline and ACE2 levels over time between RASi continuation and discontinuation (A) Inverse Kaplan-Meier plot of median sustained WHO score improvement ≥1 category from baseline for a minimum of 2 days. (B) Predictive marginal plot for ACE2 levels (logarithmized) up to follow-up week 3. A mixed effects regression model was run for RAS metabolites assuming a non-linear change over weeks. ACE2 is presented in pmol/L. ACE, angiotensin-converting enzyme; RAS, renin angiotensin-system; RASi, renin-angiotensin system inhibitor.

In the intention-to-treat analysis, median time to sustained clinical improvement on the WHO scale (≥1 category for at least two days) was 12.0 days (IQR 8.8–20.3) with RASi continuation and 17.0 days (6.5–26.5) with RASi discontinuation (p = 0.801; Table 2). Time to sustained improvement was visualized in an inverse Kaplan-Meier plot (Figure 3A). The mean WHO scores with RASi continuation vs. discontinuation were comparable at day 7 (4.10 vs. 3.69, respectively), day 14 (3.08 vs. 3.21), day 21 (2.65 vs. 2.96), and day 29 (2.50 vs. 2.43; Table 2). The MMRM over the 3-week follow-up revealed no between-group difference in the non-linear change in WHO score (p = 0.729, p = 0.878, and p = 0.175 at weeks 2, 3, and 4, respectively; (Table S2; Figure S1). In the per-protocol analysis, time to sustained WHO scale improvement was 10.0 days (5.0–12.5) in the RASi continuation and 8.5 days (6.3–19.5) in the RASi discontinuation group (p = 0.592; Table S3).

**Table 2:** Clinical outcomes stratified by clinical treatment and biomarker groups

Outcome^a^	Clinical treatment groups		Biomarker treatment groups		P	P
	RASi continuation (n = 30)	RASi discontinuation (n = 29)	RASi continuation (n = 10)	RASi discontinuation (n = 20)	RASi continuation vs. RASi discontinuation (clinical)	RASi continuation vs. RASi discontinuation (biomarker)
WHO score day 7^b^	4.10 ± 1.26	3.69 ± 1.34	4.00 ± 1.05	4.32 ± 1.29	0.212	0.580
WHO score day 14^b^	3.08 ± 1.83	3.21 ± 1.55	3.30 ± 1.57	3.67 ± 1.78	0.626	0.693
WHO score day 21^b^	2.65 ± 1.90	2.96 ± 1.64	2.40 ± 1.78	3.44 ± 1.79	0.243	0.104
WHO score day 29^b^	2.50 ± 1.79	2.43 ± 1.62	2.30 ± 1.77	3.00 ± 1.88	0.898	0.294
WHO score: sustained improvement ≥1 category from baseline for minimum 2 days (days)	12.0 (8.8–20.3)	17.0 (6.5–26.5)	14.5 (10.5–25.3)	18.0 (8.0–29.0)	0.801	0.745
NEWS: time to score ≤2 or discharge from baseline (days)	11.0 (5.0–17.3)	8.0 (4.0–20.5)	7.5 (2.8–17.0)	8.0 (3.0–29.0)	0.681	0.644
ICU admissions	8 (26.7%)	7 (24.1%)	2 (20.0%)	7 (35.0%)	0.590	0.675
ICU stay (days)	20.0 (9.0–44.0)	20.0 (12.0–23.0)	8.0 (4.0–n.a.)	23.0 (14.0–36.0)	0.714	0.118
Need for mechanical ventilation	5 (16.7%)	4 (13.8%)	1 (10.0%)	5 (25.0%)	0.759	0.633
Oxygenation free days until day 29	19.5 (11.0–25.0)	22.0 (3.8–26.0)	22.5 (16.3–27.3)	18.5 (0–25.3)	0.689	0.299
Length of hospital stay (days)	23.0 (16.8–29.5)	23.0 (15.0–34.0)	17.0 (13.5–18.5)	20.0 (13.0–30.0)	0.820	0.300
29-day mortality	1 (3.3%)	2 (6.9%)	1 (10.0%)	1 (5.0%)	0.612	1.000
90-day mortality	3 (10.0%)	2 (6.9%)	1 (10.0%)	1 (5.0%)	0.669	1.000

ICU, intensive care unit; NEWS, National Early Warning Score; RAS, renin-angiotensin system; RASi, renin-angiotensin system inhibitor; WHO, World Health Organization.

a Continuous variables are presented as medians (IQRs); binary variables are presented in absolute numbers (%). Group comparisons (p values) were performed using the Mann-Whitney U test. Dichotomous variables were compared using the chi-square or Fisher’s exact test.

b Data are presented as means (SDs).

#### Secondary endpoints

Median time to improvement in NEWS (≤2 points for >24 h or hospital discharge) was 11.0 days (IQR 5.0–17.3) with RASi continuation and 8.0 days (4.0–20.5) with RASi discontinuation (p = 0.681; Figure S2). ICU admission rates were equally distributed with 26.7% (n = 8) admitted in the RASi-continuation group and 24.1% (n = 7) in the discontinuation group (p = 0.590). The groups also were similar in median time in the ICU, at 20 days (IQR 9.0–44.0) with RASi continuation and 20 days (IQR 12.0–23.0) with discontinuation (p = 0.714). In the RASi continuation group, 16.7% (n = 5) required mechanical ventilation, compared with 13.8% (n = 4) in the discontinuation group. The 29-day mortality rates were 3.3% (RASi continuation) and 6.9% (RASi discontinuation), respectively (p = 0.612), and 90-day mortality rates were 10.0% and 6.9% (p = 0.669). Median time to hospital discharge was 23 days in both groups (p = 0.820, Table 2). Information on antihypertensive and COVID-19 treatment as well as laboratory outcomes are provided in the Tables S4 and S5; Figure S3.

### Non-protocol based RASi discontinuation

In four patients (13.3%) randomized to the RASi continuation group, ACEi or ARB treatment was subsequently discontinued by the treating physicians following admission to the ICU. To further assess RASi discontinuation patterns in patients hospitalized with COVID-19, we performed a post-hoc analysis of study participants enrolled in the ACOVACT trial who were on RASi medication at the time of hospitalization but not enrolled in the RASi intervention arm of the study; this analysis identified 33 patients (Table S6; Figure S4). In this group, RASi medication was discontinued in 20 patients (60.6%) following hospitalization, whereas 13 (39.4%) remained on RASi during the hospital stay. A clinical indication for discontinuing RASi such as hypotension/vasopressor therapy or renal dysfunction could be identified in only 7 of the 20 discontinued patients (35.0%; Table S7).

### Biomarker outcomes

Biomarker analyses were performed as-treated in a cohort of 30 patients with at least two RAS profile measurements per patient. A total of 121 measurements across all patients were available, with a median of 3.0 (IQR 2.0–5.0) measurements per patient. There was no difference in overall median ACE2 and RAS metabolite levels between RASi continuation and discontinuation (Table 3). The MMRM over 3 weeks suggested a tendency for ACE2 concentrations to increase in both groups but without differences between them (Figure 3B; Table S8).

**Table 3:** RAS metabolite levels in the biomarker groups

RAS metabolites^a^	Treatment Group	Median (IQR)	P RASi continuation vs. RASi discontinuation
ACE2			0.895
	RASi continuation	3.53 (3.97)	
	RASi discontinuation	3.15 (6.35)	
ACE-S			0.086
	RASi continuation	0.31 (1.87)	
	RASi discontinuation	1.50 (1.54)	
Ang 1-7			0.946
	RASi continuation	10.15 (25.02)	
	RASi discontinuation	8.74 (44.81)	
Ang 1-5			0.084
	RASi continuation	5.55 (13.24)	
	RASi discontinuation	11.54 (38.96)	
Ang I			0.395
	RASi continuation	212.21 (370.66)	
	RASi discontinuation	114.31 (241.86)	
Ang II			0.137
	RASi continuation	23.80 (118.06)	
	RASi discontinuation	126.45 (212.43)	
PRA-S			0.772
	RASi continuation	280.36 (474.67)	
	RASi discontinuation	272.80 (605.60)	

ACE, angiotensin-converting enzyme; Ang, angiotensin; PRA-S, plasma renin activity; RAS, renin-angiotensin system; RASi, renin-angiotensin system inhibitor.

a RAS metabolites are presented as overall median (IQR) measurements over time per patient. RASi continuation and RASi discontinuation were compared (p values) using the Mann-Whitney U test. Absolute values were log-transformed for analyses. PRA-S and ACE-S are presented in pM, all other angiotensin values are reported in pmol/L.

**Figure 4:**
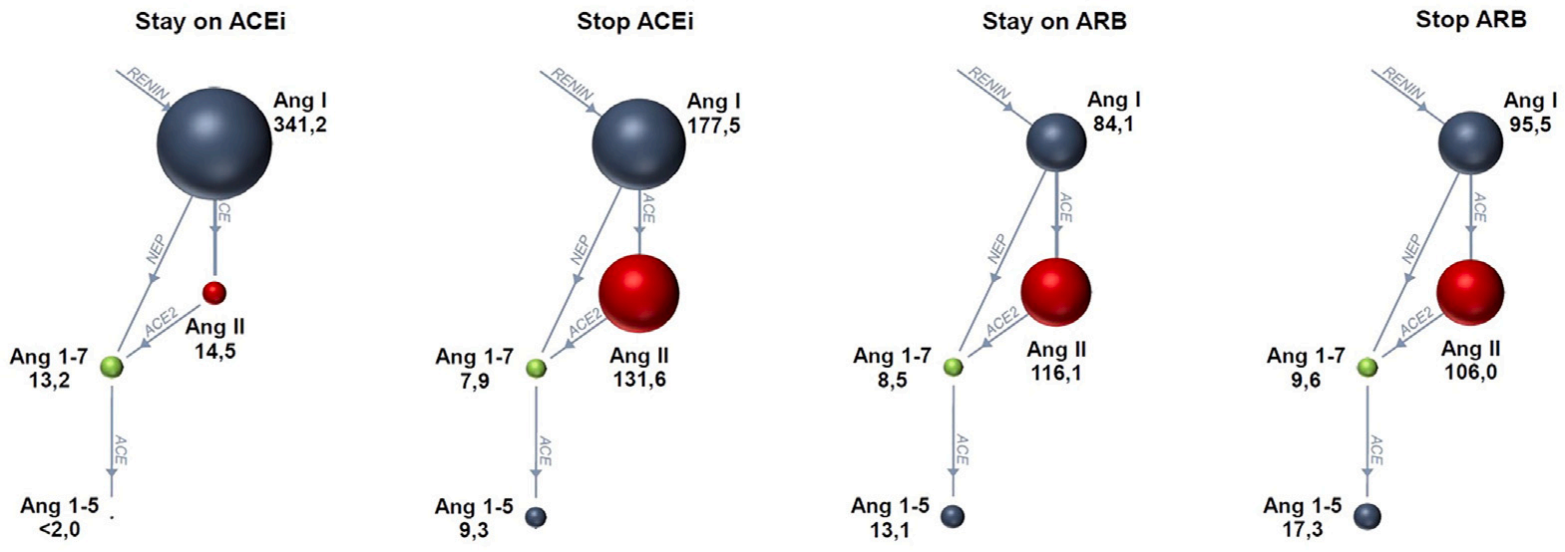
RAS metabolites illustrated for overall median measurements stratified by the specific RASi agent Absolute values are presented as medians from overall median measurements over time per patient within the respective group. All angiotensin values are reported in pmol/L. ACE, angiotensin-converting enzyme; ACEi, angiotensin-converting enzyme inhibitor; Ang, angiotensin; ARB, angiotensin receptor blocker; RAS, renin-angiotensin system; RASi, renin-angiotensin system inhibitor.

In a comparison of median baseline vs. median follow-up values for each patient, ACE2 increased significantly in both, the RASi continuation group (2.33 (IQR 1.23–4.56) pmol/L to 4.03 (IQR 3.23–6.09) pmol/L; p = 0.028) as well as the discontinuation group (2.25 (1.22–3.58) pmol/L to 3.61 (2.08–9.15) pmol/L; p < 0.001; Table S9). In a comparison of patients stratified by COVID-19 severity, ACE2 was higher in those with severe (maximum WHO Score 5–7) vs. mild (maximum WHO Score 3–4) disease (2.33 (1.77–4.22) pmol/L vs. 4.63 (2.63–41.03) pmol/L; p = 0.063; Table S9). Ang II, Ang 1–5 levels and ACE-S activity increased with ACEi discontinuation. All RAS metabolite levels remained unaltered following ARB discontinuation (Table S10; Figure 4).

## Discussion

RASi continuation or discontinuation in patients hospitalized with COVID-19 did not modify the course of disease or the clinical outcome in our randomized controlled trial. Furthermore, ACE2 levels did not differ between patients who continued RASi therapy and those who discontinued, but were increased in both groups. Overall, patients with severe COVID-19 had the highest ACE2 levels, in line with our previous data.^13^^,^^14^ Of note, we observed a high rate of RASi discontinuation in patients with severe disease, which could reflect a more general behavioral pattern with respect to RASi treatment in patients presumed to be critically ill.

Overall, our data correspond with findings from other recent large clinical trials on RASi continuation and discontinuation showing no difference in clinical outcomes; these trials include BRACE CORONA (n = 659), REPLACE COVID (n = 152), and ACEI-COVID (n = 204).^28^^,^^29^^,^^30^ In the REPLACE COVID trial, 17 of 75 (22.6%) patients randomized to stay on RASi treatment subsequently had their ACEi or ARB therapy discontinued before reaching a study endpoint. A meta-analysis by the International Society of Hypertension including 14 randomized controlled trials representing 1,838 individuals also revealed no change in all-cause mortality.^31^

Previous analyses have suggested that ACE2 levels are elevated in patients on ACEis or ARBs.^32^^,^^33^ We recently published systemic biomarker results showing significantly elevated ACE2 levels in severe COVID-19, regardless of RASi medication. ACE2 values have been reported to be highest in patients on mechanical ventilation.^14^ The current findings add to this picture with longitudinally measured RAS metabolite profiles, including ACE2 levels, in a randomized controlled trial setting comparing RASi continuation and discontinuation.

The observed changes in RAS profiles following RASi discontinuation primarily corresponded with the known mechanisms of action of ACEi and ARB therapies, with ACE-S and Ang II increasing significantly after a switch to a non-RASi medication. In addition, Ang I values were higher in patients with ACEi continuation, through the inhibited conversion of Ang I to Ang II. Ang 1–7 levels were also higher in patients continuing ACEis by inhibiting a degradation to Ang 1–5.^16^^,^^34^ In those who discontinued ACEi therapy, Ang 1–5 increased.

Functional analysis of RAS metabolites has offered new and unique insights into the effects of RAS-blocking medication on RAS activity in COVID-19 patients before and after intervention. The core strength of this study is the randomized controlled trial design and the longitudinal sampling for RAS metabolite analysis. The study population included patients with a wide range of disease severity ranging from no oxygen supplementation to mechanical ventilation. The overall number of patients included in the trial was rather small, but clinical outcomes are similar to those described in larger trials, supporting the validity of the current biomarker results.

ACE2 levels were unaltered by RASi discontinuation in our randomized controlled trial and were mainly influenced by disease severity. Clinical outcomes did not seem to be affected by RASi continuation vs. discontinuation, which is consistent with larger trials. Our observed changes in RAS metabolite levels reflect the specific mechanisms of action of ACEis and ARBs. The high rate of non-protocol-based RASi withdrawal in the study population has been reported in other trials, suggesting a general behavioral pattern in critically ill patients. We recommend raising awareness about discontinuing RASi only for a clinically reasonable indication. From the clinical and metabolic perspectives, our findings clearly support continuation of ACEi and ARB therapy in patients with COVID-19.

### Limitations of the study

The study was not powered to draw definite conclusions on clinical outcomes using small sample sizes, which differed between the clinical and biomarker analysis. This difference results by the inclusion of patients with at least two metabolite measurements within two subsequent follow-up weeks in the biomarker treatment groups. Furthermore, the quantification process of RAS metabolites is costly and had to be considered in the sample size calculation. Another reason for the smaller sample size resulted from the high challenging study conditions during the pandemic state. Laboratory samples had to be correctly stored multiple times per week and transported for RAS quantification. As the medical staff had to work at their physical and psychological limits, fewer RAS samples could be collected and subsequently analyzed as initially anticipated. All blood samples collected during the morning routine were fully considered for the RAS quantification procedure. Again, due to the overwhelming COVID-19 situation for the hospital stuff, follow-up swabs were not regularly performed and viral clearance was therefore not considered as a secondary endpoint. The non-protocol based RASi discontinuation was also a limitation in our study and can be mainly explained by the rapid clinical deterioration of numerous patients. Four out of ten protocol violations were caused by a discontinuation of RASi due to the transfer to the ICU requiring vasopressive agents. Four other patients were not switched to a non-RASi medication. Two more patients were excluded due to a *de novo* initiation of RASi in the RAS continuation cohort. As a consequence, we performed a per-protocol analysis to strengthen our clinical outcomes. Protocol violations seem to have occured frequently during the COVID-19 pandemic, as the BRACE Corona trial already registered an exclusion rate of 11% and a cumulative non-adherence rate of 8.8%.^28^ These common challenges in the clinical routine during the pandemic prompted us to conduct an additional post-hoc analysis of other study patients. The linear measurement model of RAS metabolites must be interpreted with caution because the RAS highly reacts to several clinical conditions and drug specific interactions. For this reason, both linear and descriptive overall RAS metabolite measurements were reported.

## STAR★Methods

### Key resources table

**Table:** 

REAGENT or RESOURCE	SOURCE	IDENTIFIER
**Biological samples**		

RAS blood probes	Attoquant	https://www.attoquant.com/

**Software and algorithms**		

R Version 4.0.1	R Foundation	https://www.r-project.org/
Stata Statistics Version 17	Stata Corp	https://www.stata.com/
SPSS Statistics Version 26	IBM	https://www.ibm.com/

### Resource availability

#### Lead contact

Further information and requests for resources and reagents should be directed to and will be fulfilled by the lead contact, Manfred Hecking (manfred.hecking@meduniwien.ac.at).

#### Materials availability

This study did not generate new unique reagents.

### Experimental model and study participant details

The study population comprised patients on RASi medication for chronic hypertension who were hospitalized with polymerase chain reaction–confirmed SARS-CoV-2 infection. Exclusion criteria for study participation were life expectancy <1 month (e.g., terminal disease), patients not qualifying for intensive care, pregnancy or breast feeding, severe liver disease, chronic kidney disease stage >4, anticipated hospital discharge <48 h, or severe chronic heart failure.

The study was conducted as substudy B of the Austrian Coronavirus Adaptive Clinical Trial (ACOVACT; URL: https://www.clinicaltrials.gov; NCT number: NCT04351724, EudraCT number: 2020-001302-30)^35^ and was approved by the ethics committee of the Medical University of Vienna. A written informed consent was obtained from each participant of the study. The trial protocol is provided in the supplemental information section.

#### Main study interventions

In the main study of ACOVACT, three antiviral treatment arms were implemented: The hydroxychloroquine arm, which was closed early after negative outcome trials, the lopinavir/ritonavir arm (initially given at standard dose and after a protocol amendment administered at high dosage) and the third arm with camostat mesylate, considered as standard-of-care. The randomization ratio was 1:1 after rapid discontinuation of the hydroxychloroquine treatment arm. More detailed information can be obtained from the trial protocol (supplemental information).

In addition to substudy B, two further substudies were conducted: Substudy A investigated the administration of rivaroxaban 5mg daily versus low-molecular weight heparin in a prophylactic dose. In substudy C, patients were randomized to receive asunercept at 25mg, 100mg or 400mg per week or standard-of-care therapy.

### Method details

#### Clinical outcomes

Clinical improvement was assessed using the World Health Organization (WHO) seven-category ordinal scale, defined as follows at the time of protocol development: 1. Not hospitalized, no limitations on activities; 2. Not hospitalized, limitation on activities; 3. Hospitalized, not requiring supplemental oxygen; 4. Hospitalized, requiring supplemental oxygen; 5. Hospitalized, on non-invasive ventilation or high-flow oxygen devices; 6. Hospitalized, on invasive mechanical ventilation or extracorporeal membrane oxygenation; 7. Death. The primary clinical endpoint was time to clinical improvement, defined as time from randomization to sustained improvement by at least one category on two consecutive days compared with baseline.

As secondary clinical endpoints, we assessed the National Early Warning Score (NEWS) in hospitalized patients, including respiratory rate, oxygen saturation, supplemental oxygen, temperature, blood pressure, heart rate, and level of consciousness,^36^ as well as hospital length of stay (LOS), admission to the intensive care unit (ICU), time in ICU, requirement for mechanical ventilation, and death.

Scores were re-evaluated daily, with documentation of the worst clinical findings of the day. On day 29 and day 90 after randomization, sustained health improvement in discharged patients was assessed by a telephone visit. Adverse and serious adverse events were documented until day 90 (supplemental information). Clinical outcomes were analysed according to randomized treatment assignment.

#### Biomarker analysis

The primary objective was to determine a change in ACE2 levels following RASi continuation or discontinuation. We further assessed differences in RAS metabolite levels (i.e., Ang I, Ang II, Ang 1-7, Ang 1-5; ACE activity (ACE-S); plasma renin activity (PRA-S); and ACE2) between groups stratified by RASi medication (ACEi and ARB) and disease severity. Biomarker analyses were performed as-treated.

#### Procedures

Patients were randomized to either continue their previous RASi medication or to switch to a non-RASi drug. Target blood pressure levels were <140/90 mmHg.

#### Post hoc analyses

For post hoc analyses of RASi discontinuation rates in patients hospitalized with COVID-19, we also analysed patients on RASi in a non-randomized control group within the ACOVACT trial who were not enrolled in the RASi intervention substudy.

#### Sample collection and processing, measurement of RAS metabolites

Blood samples for RAS assessment were collected up to three times weekly in hospitalized patients and immediately transported to the Institute of Virology of the Medical University of Vienna. Samples were centrifuged under biosafety level 2 conditions to obtain serum, which then was virus-inactivated and stored frozen at -20°C. Serum samples were then transferred to Attoquant Diagnostics laboratory for quantitative analysis. Under incubation at 37°C, the RAS peptides were stabilized at equal formation and degradation rate by enzyme-inhibiting cocktails. Under these equilibrated levels, angiotensin metabolites were further analysed using liquid chromatography-mass spectrometry/mass-spectroscopy. The high concentration of angiotensinogen in human plasma results in a steady Ang I formation without substantial angiotensinogen degradation, providing a stable condition for *ex vivo* quantification.^37^ Equilibrium RAS analysis has been shown to detect higher angiotensin levels than circulating peptides in plasma and to correlate highly with quantified metabolites that are stabilized by an enzyme inhibitor during blood sampling.^37^^,^^38^^,^^39^ The biochemical background of the equilibrium approach have previously been validated in chronic kidney and heart disease.^38^^,^^40^ Further details on the diagnostic procedures are given in the supplemental information section. PRA-S was calculated by Ang I + Ang II, and ACE-S was calculated using the quotient of Ang II/Ang I.

### Quantification and statistical analysis

Demographics of the study participants were summarized as medians (interquartile range (IQR)) for continuous variables and means (standard deviation (SD)). Absolute and relative frequencies were used for categorical parameters.

Clinical endpoints were analysed in an intention-to-treat analysis. The primary endpoint was visualized in a Kaplan-Meier plot. The comparisons of the time-to-event curves were performed using a log-rank test at a two-sided alpha level of 5%. Furthermore, changes in median WHO scores over time were compared using a mixed linear regression model for repeated measurements (MMRM) at baseline and the weeks after hospitalization (up to follow-up week 4), adjusted for age, sex, and baseline values.

The analyses of all secondary endpoints (NEWS, LOS, need for mechanical ventilation, death) were considered as exploratory endpoints, and no correction for multiple testing was performed. Time to sustained improvement in NEWS (decrease ≤2 points from baseline for >24 h or hospital discharge) was visualized in a Kaplan-Meier plot and compared between the RASi continuation and discontinuation group using the log-rank test. LOS, ICU stay, and median WHO score/NEWS were compared using the Mann-Whitney U test and dichotomic variables with the chi square or Fisher’s exact test.

For the biomarker analysis, only individuals with at least two measurements within two subsequent follow-up weeks were included. ACE2 levels over time were assessed by applying the MMRM. In addition, overall median (IQR) measurements per patient were compared between the groups as well as median (IQR) baseline RAS measurements per patient with median measurements over time per patient (follow-up) using the Wilcoxon test and Mann-Whitney U test. Subgroups were stratified by mild (maximum WHO score 3-4) and severe (maximum WHO score 5–7) COVID-19, and median measurements over time per patient were compared using the Mann-Whitney U test. Further subgroups were stratified for the specific RASi agent (ACEi/ARB), and median values were also tested with the Mann-Whitney U test.

For statistical analyses and plot design, IBM SPSS Statistics Version 26, Stata Statistics Version 17, and R Version 4.0.1 were used.

## Data Availability

• This paper does not report any original code. • All data reported in this paper will be shared by the lead contact upon reasonable request. • Any additional information required to reanalyze the data reported in this paper is available from the lead contact upon reasonable request.

## Supplemental information

Document S1. Figures S1–S4 and Tables S1–S10

Document S2. ACOVACT study protocol
