# Supplementary material for: Renin-angiotensin system inhibitor discontinuation in COVID-19 did not modify systemic ACE2 in a randomized controlled trial
Source: iScience. 2023 Oct 5;26(11):108146. doi: 10.1016/j.isci.2023.108146 (PMC10585392; doi:10.1016/j.isci.2023.108146)
Supplement: Document S1. Figures S1–S4 and Tables S1–S10 [file mmc1.pdf]

iScience, Volume *26*

Supplemental information

Renin-angiotensin system inhibitor discontinuation in COVID-19 did not modify systemic ACE2 in a randomized controlled trial

Vincent Rathkolb, Marianna T. Traugott, Andreas Heinzel, Marko Poglitsch, Judith Aberle, Farsad Eskandary, Agnes Abrahamowicz, Martin Mueller, Petra Knollmueller, Tarik Shoumariyeh, Jasmin Stuflesser, Ivan Seeber, Georg Gibas, Hannah Mayfurth, Viktoria Tinhof, Lukas Schmoelz, Markus Zeitlinger, Christian Schoergenhofer, Bernd Jilma, Bernd Genser, Wolfgang Hoepler, Sara Omid, Mario Karolyi, Christoph Wenisch, Rainer Oberbauer, Alexander Zoufaly, Manfred Hecking, and Roman Reindl-Schwaighofer

Supplemental information

Supplementary methods

Equilibrium approach for angiotensin quantification

Serum conditioning for equilibrium analysis was performed at 37°C followed by sample stabilization through blocking angiotensin metabolism (Attoquant Diagnostics, Vienna, Austria). For quantification of angiotensin levels, equilibrated and stabilized serum samples were spiked with stable isotope-labeled internal standards for each individual angiotensin metabolite. The samples then were subjected to C-18–based solid-phase extraction and liquid chromatography-mass spectrometry/mass-spectroscopy analysis using a reversed-phase analytical column operating in line with a Xevo TQ-S triple quadruple mass spectrometer (Waters, Milford, MA, USA). Internal standards were used to correct for peptide recovery of the sample preparation procedure for each analyte in each individual sample. Analyte concentrations were reported in pmol/L (pM) and were calculated considering the corresponding response factors determined in appropriate calibration curves in the original sample matrix, given that integrated signals exceeded a signal-to-noise ratio of 10.

Active ACE2 concentration (in plasma) was determined in diluted samples (phosphate-buffered saline, pH 7.4 / 5 mM $ZnCl_2$) after samples were spiked with angiotensin II as the natural substrate, with subsequent incubation at 37°C in the presence and absence of the specific ACE2 inhibitor MLN-4760 (10 µmol/L, Sigma-Aldrich, Munich, Germany). Aminopeptidase inhibitor, Z-Pro-prolinal, and lisinopril (all 10 µmol/L, Sigma-Aldrich) were added to all samples for substrate and product (angiotensin 1-7) stabilization. Quantification of angiotensin II and angiotensin 1-7 was conducted using liquid chromatography-mass spectrometry/mass-spectroscopy as described above. The ACE2-specific angiotensin 1-7 formation rate was calculated, and the active ACE2

concentration was determined via a calibration curve of recombinant human ACE2 (R&D Systems, Minneapolis, MN, USA) in human serum.

Equilibrium analysis is feasible from standard heparinized plasma or serum samples. In the equilibrium analysis, angiotensin product-to-substrate ratios directly reflect the activity of the corresponding enzymes, with absolute equilibrium levels providing information on the state of the soluble RAS. Of note, the concentrations of ACE2 can also be measured from serum using Mca-Ala-Pro-Lys at room temperature. The alternative RAS can be “boosted” by RAS-interfering agents.

Supplementary tables

Table S1, related to Table 1: Entire specification for the charlson comorbidity index (CCI) between the main treatment groups. [a]

Parameter	RASi continuation (n=30)	RASi discontinuation (n=29)
**Charlson Comorbidity Index**		
CCI score [b]	3.5±1.9	3.3±1.9
Myocardial infarction	3 (10.0%)	2 (6.9%)
Congestive heart failure	3 (10.0%)	2 (6.9%)
Peripheral vascular disease	3 (10.0%)	2 (6.9%)
Cerebrovascular accident or transient ischemic attack	4 (13.3%)	2 (6.9%)
Dementia	0	0
Chronic obstructive pulmonary disease	4 (13.3%)	3 (10.3%)
Connective tissue disease	2 (6.7%)	1 (3.4%)
Peptic ulcer disease	2 (6.7%)	0
Mild liver disease	0	0
Uncomplicated diabetes	9 (30,0%)	9 (31.0%)
Hemiplegia	0	0
Moderate to severe chronic kidney disease	0	2 (6.9%)
Diabetes with end-organ damage	2 (6.7%)	2 (6.9%)
Localized solid tumor	6 (20.0%)	4 (13.8%)
Leukemia	0	0
Lymphoma	0	0
Moderate to severe liver disease	0	0
Metastatic solid tumor	0	0
AIDS	0	0

Abbreviations: RAS, renin-angiotensin system; RASi, renin-angiotensin system inhibitor.
[a] Continuous variables are presented as medians (IQRs); binary variables are presented in absolute numbers (%).

[b] Data are presented as means (SDs).

Table S2, related to Table 2 and Figure S1: WHO score over time assuming a non-linear change up to follow-up week 3. [a]

Parameter	Measurement	z	P>|z|	95% CI
**WHO Score**	RASi discontinuation baseline	0.11	0.915	[-0.547; 0.610]
	RASi discontinuation week 1	-0.53	0.593	[-0.489; 0.280]
	RASi discontinuation week 2	0.35	0.729	[-0.323; 0.461]
	RASi discontinuation week 3	0.15	0.878	[-0.388; 128]
	RASi discontinuation week 4	-1.36	0.175	[-0.720; 0.131]
	Baseline WHO score	3.79	0.000	[0.326, 1.024]
	Male	0.52	0.602	[-0.358; 0.617]
	Age	0.37	0.710	[-0.018; 0.027]

Abbreviations: CI, confidence interval; RAS, renin-angiotensin system; RASi, renin-angiotensin system inhibitor; WHO, World Health Organization.

[a] Model: Mixed-effects regression model for the WHO score assuming a non-linear change over weeks using “RASi continuation” as reference group. Intention-to-treat analysis was performed. The WHO scale (1-7) is unitless.

Table S3, related to Table 2: Clinical outcomes stratified by clinical treatment and biomarker groups in the per-protocol analysis. [a]

Outcome	Clinical treatment groups		P
	RASi continuation (n=24)	RASi discontinuation (n=25)	RASi continuation vs. RASi discontinuation
WHO score day 7 [b]	3.81±1.17	3.79±1.25	0.770
WHO score day 14 [b]	2.62±1.63	3.21±1.50	0.145
WHO score day 21 [b]	2.19±1.60	3.00±1.56	0.024
WHO score day 29 [b]	2.14±1.59	2.54±1.64	0.206
WHO score: sustained improvement ≥1 category from baseline for minimum 2 days (days)	10.0 (5.0-12.5)	8.5 (6.3-19.5)	0.592
NEWS: time to score ≤2 or discharge from baseline (days)	7.0 (4.0-11.0)	7.0 (3.5-12.5)	0.771
ICU admissions	4 (16.7%)	5 (20.0%)	0.763
ICU stay (days)	12.0 (4.0-n.a.)	21.5 (8.8-23.0)	0.721
Need for mechanical ventilation	2 (8.3%)	3 (12.0%)	0.672
Oxygenation free days until day 29	22.0 (17.5-26.0)	21.5 (3.8-26.0)	0.508
Length of hospital stay (days)	12.0 (11.0-17.0)	18.0 (11.0-27.0)	0.090
29-day mortality	1 (4.2%)	2 (8.0%)	0.576
90-day mortality	1 (4.2%)	0	0.302

Abbreviations: ICU, intensive care unit; NEWS, National Early Warning Score; RAS, renin-angiotensin system; RASi, renin-angiotensin system inhibitor; WHO, World Health Organization.

[a] Continuous variables are presented as medians (IQRs); binary variables are presented in absolute numbers (%). Group comparisons (p values) were performed using the Mann–Whitney U test. Dichotomous variables were compared using the chi-square or Fisher's exact test.

[b] Data are presented as means (SDs).

Table S4, related to Table 1: Specifics regarding antihypertensive and COVID-19 related treatment in the RASi continuation and discontinuation group during hospitalization. [a]

Group	Drug	Daily dosage	n
**RASi continuation (n=30)**			
	**ACEi**		
	Lisinopril	15.0 (8.8–20.0)	10
	Ramipril	3.8 (2.5–6.3)	6
	Enalapril	20.0 (n.a.)	1
	**ARB**		
	Candesartan	12.0 (8.0–16.0)	8
	Valsartan	160.0 (160.0–180.0)	5
	Irbesartan	300.0 (n.a.)	1
	**Cortisone drug (i.v.)**		
	Dexamethasone	6.0 (6.0–6.0)	21
	Prednisolone	27.2 (n.a)	2
	Hydrocortisone	257.5 (n.a.)	1
	**Cortisone drug (inhaled)**		
	Budesonide	320.0 (n.a.)	1
	Beclomethasone dipropionate	200.0 (n.a.)	2
	Fluticasone propionate	500.0 (n.a.)	2
	**Calcium antagonist**		
	Amlodipine	-	12 (40.0%)
	Nitrendipine	-	3 (10.0%)
	Lercanidipine	-	1 (3.3%)
	**Beta-blocker**		
	Bisoprolol	-	4 (13.3%)
	Metoprolol	-	3 (10.0%)
	Nebivolol	-	5 (16.7%)
	Atenolol	-	1 (3.3%)
	Propranolol	-	1 (3.3%)
	Carvedilol	-	2 (6.7%)
	**Aldosterone antagonist**		
	Spironolactone	-	2 (6.7%)

	**Diuretic**		
	Furosemide	-	7 (23.3%)
	Hydrochlorothiazide	-	8 (26.7%)
	Xipamide	-	0
	**Alpha-blocker**		
	Doxazosin	-	5 (16.7%)
	Urapidil	-	1 (3.3%)
	**Alpha agonist**		
	Clonidine	-	1 (3.3%)
	**Central antisympathicotonic**		
	Rilmenidine	-	2 (6.7%)
	**Total antihypertensive agents [b]**	-	1.3±1.5
**RASi discontinuation (n=29)**			
	**ACE inhibitor**		
	Lisinopril	7.5 (n.a.)	2
	Ramipril	2.5 (1.3–5.0)	3
	Fosinopril	20.0 (n.a.)	1
	Enalapril	20.0 (n.a.)	1
	Zofenopril	30.0 (n.a.)	1
	**ARB**		
	Candesartan	16.0 (8.0–16.0)	15
	Valsartan	160.0 (120.0–160.0)	5
	Losartan	50.0 (n.a.)	1
	**Cortisone drug (i.v.)**		
	Dexamethasone	6.0 (6.0–6.0)	15
	Prednisolone	46.9 (14.7–50.0)	4
	Solu Dacortin	300.0 (n.a.)	1
	**Cortisone drug (inhaled)**		
	Budesonide	1280.0 (n.a)	1
	Fluticasone propionate	100.0 (n.a.)	2
	Beclomethasone dipropionate	174.0 (93.5–300.0)	5
	**Calcium antagonist**		
	Amlodipine	-	9 (31.0%)
	Nitrendipine	-	19 (65.5%)

Lercanidipine	-	1 (3.4%)
**Beta-blocker**		
Bisoprolol	-	3 (10.3%)
Metoprolol	-	2 (6.9%)
Nebivolol	-	2 (6.9%)
Atenolol	-	2 (6.9%)
Propranolol	-	1 (3.4%)
Carvedilol	-	0
**Aldosterone antagonist**		
Spironolactone	-	1 (3.4%)
**Diuretic**		
Furosemide	-	8 (27.6%)
Hydrochlorothiazide	-	8 (26.7%)
Xipamide	-	0
**Alpha-blocker**		
Doxazosin	-	5 (16.7%)
Urapidil	-	1 (3.3%)
**Alpha-agonist**		
Clonidine	-	1 (3.3%)
**Central antisympathicotonic**		
Rilmenidine	-	2 (6.7%)
Hydrochlorothiazide	-	8 (26.7%)
Xipamide	-	0
**Alpha-blocker**		
Doxazosin	-	5 (16.7%)
Urapidil	-	2 (6.9%)
**Alpha-agonist**		
Clonidine	-	1 (3.4%)
**Central antisympathicotonic**		

Abbreviations: ACEi, ACE inhibitor; ARB, angiotensin receptor blocker; RAS, renin-angiotensin system; RASi, renin-angiotensin system inhibitor.

[a] Continuous variables are presented as medians (IQRs); binary variables are presented in absolute numbers (%).

[b] Data are presented as means (SDs).

Table S5, related to Table 2 and Figure S3: Clinical and laboratory outcome parameters in the main treatment groups. [a]

Outcome	RASi continuation (n=30)	RASi discontinuation (n=29)	P RASi continuation vs. RASi discontinuation
Systolic blood pressure (mmHg)	125.0 (120.0–130.0)	129.0 (125.0–130.0)	0.052
Diastolic blood pressure (mmHg)	70.0 (65.0–71.0)	71.0 (70.0–77.0)	0.003
SpO_2 (%)	95.0 (95.0–96.0)	95.0 (95.0–95.5)	0.254
Leukocytes (G/L)	9.4 (8.2–11.3)	8.7 (7.6–10.1)	0.095
Platelets (G/L)	285.5 (227.0–343.5)	236.0 (215.0–269.5)	0.009
Erythrocytes T/L	3.6 (3.1–4.4)	4.1 (3.7–4.5)	0.026
Hemoglobin g/dl	10.4 (9.2–12.8)	12.0 (10.7–12.8)	0.051
Neutrophils (G/L)	7.3 (5.8–8.3)	5.8 (4.5–7.7)	0.056
Lymphocytes (G/L)	1.4 (1.0–1.6)	1.2 (1.1–1.5)	0.276
CRP (mg/L)	29.6 (16.1–48.2)	29.6 (12.4–53.3)	0.994
Sodium (mmol/L)	139.5 (138.8–140.8)	140.0 (139.0–141.0)	0.397
Potassium (mmol/L)	4.2 (4.0–4.4)	4.0 (3.9–4.1)	0.005
Creatinine (mg/dl)	0.8 (0.7–0.9)	0.7 (0.7–0.8)	0.304
Glomerular filtration rate (ml/min/1.7 m^2)	85.9 (77.5–89.5)	90.0 (85.4–90.0)	0.002
Creatine kinase (U/L)	45.0 (24.5–76.5)	57.5 (31.5–74.5)	0.359
Troponin-T (µg/L)	40.0 (18.4–83.3)	14.2 (11.6–72.0)	0.093

Abbreviations: CRP, C-reactive protein; RAS, renin-angiotensin system; RASi, renin-angiotensin system inhibitor; SpO_2, oxygen saturation.

[a] Continuous variables are presented as medians (IQRs). Group comparisons (p values) were performed using the Mann-Whitney U test.

Table S6, related to Table 1 and Figure S4: Baseline characteristics and demographics in ACOVACT included non-substudy B patients treated with RASi. [a]

Parameter	RASi discontinued (n=20)	RASi continued (n=13)
**Demographics**		
Male	18 (90.0%)	9 (69.2%)
Female	2 (10.0%)	4 (30.8%)
Age (y)	60.5 (51.8–76.3)	68.0 (53.5–78.0)
BMI (kg/m^2)	30.7 (25.9–34.9)	29.6 (25.6–33.8)
Obesity	10 (50.0%)	4 (30.8%)
Charlson Comorbidity Index score [b]	3.8±2.6	3.5±2.1
Chronic kidney disease (stage G2 to G3b)	3 (15.0%)	1 (7.7%)
**COVID-19–related medication**		
Camostat	6 (30.0%)	7 (53.8%)
Lopinavir/Ritonavir	13 (65.0%)	5 (38.5%)
Hydroxychloroquine	1 (5.0%)	1 (7.7%)
Remdesivir	7 (35.0%)	5 (38.5%)
Pooled plasma	3 (15.0%)	1 (7.7%)
Asunercept	0	1 (7.7%)
Cortisone intravenously	15 (75.0%)	11 (84.6%)
Cortisone inhaled	1 (5.0%)	1 (7.7%)
**RASi agents prior to admission**		
ACEi	11 (55.0%)	7 (53.8%)
ARB	9 (45.0%)	6 (46.2%)

Abbreviations: ACEi, ACE inhibitor; ARB, angiotensin receptor blocker; BMI, body mass index; RAS, renin-angiotensin system; RASi, renin-angiotensin system inhibitor.

[a] Continuous variables are presented as medians (IQRs); binary variables are presented in absolute numbers (%).

[b] Data are presented as means (SDs).

Table S7, related to Table 2: Clinical outcome parameters for the non-substudy B RASi continuation and RASi discontinuation groups. [a]

Outcome	RASi discontinued (n=20)	RASi continued (n=13)
Time from hospital admission to discontinuation (days) [b]	1.6±2.6	–
Time from hospital admission to RASi discontinuation (days) [b]	1.6±2.6	–
Median time from hospital admission to RASi discontinuation (days)	0.5 (0.0–2.0)	–
**Events for discontinuation**		
Renal dysfunction	3 (15.0%)	–
Post kidney failure	1 (5.0%)	–
Vasopressor therapy	3 (15.0%)	–
Missing indication for discontinuation	13 (65.0%)	–
**Blood pressure** [b]		
Systolic blood pressure (mmHg)	126.0±8.8	131.7±8.8
Diastolic blood pressure (mmHg)	73.9±8.1	76.4±6.9
**Laboratory values** [b]		
Potassium (mmol/L)	4.0±0.4	3.8±0.5
Creatinine (mg/dl)	1.1±0.4	0.9±0.2
Glomerular filtration rate (ml/min/1.7 m^2)	72.3±19.2	78.9±10.9
**RASi agents**		
**ACEi discontinued**	11 (55.0%)	–
Ramipril	5 (25.0%)	–
Lisinopril	3 (15.0%)	–
Enalapril	2 (10.0%)	–
Fosinopril	1 (5.0%)	–
**ARB discontinued**	9 (45.0%)	–
Candesartan	5 (25.0%)	–
Valsartan	3 (15.0%)	–
Losartan	1 (5.0%)	–
**ACEi continued**	–	7 (53.8%)
Lisinopril	–	3 (23.1%)
Ramipril	–	2 (15.4%)
Enalapril	–	2 (15.4%)

**ARB continued**	–	6 (46.2%)
Candesartan	–	3 (23.1%)
Valsartan	–	3 (23.1%)
**Total antihypertensive agents during hospital stay** [b]	2.3±2.1	1.8±2.0
**Clinical outcome**		
WHO score [b]	3.9±0.6	3.3±0.5
NEWS [b]	4.3±1.3	3.4±1.4
Maximum WHO score 3	0	1 (7.7%)
Maximum WHO score 4	6 (30.0%)	9
Maximum WHO score 5	10 (50.0%)	2
Maximum WHO score 6	4 (20.0%)	1 (7.7%)
Death	1 (5.0%)	1 (7.7%)
ICU admission	9 (45.0%)	1 (7.7%)
Intubated	3 (15.0%)	0
Received vasopressors	3 (15.0%)	0
Total hospital stay (days)	19.0 (11.0–27.8)	11.0 (7.5–17.5)

Abbreviations: ACEi, ACE inhibitor; ARB, angiotensin receptor blocker; BMI, body mass index; COVID-19, coronavirus disease 2019; ICU, intensive care unit; NEWS, National Early Warning Score; RAS, renin-angiotensin system; RASi, renin-angiotensin system inhibitor; WHO, World Health Organization.

[a] Continuous variables are presented as medians (IQRs); binary variables are presented in absolute numbers (%).

[b] Data are presented as means (SDs).

Table S8, related to Table 3 and Table S9: RAS metabolites over time assuming a non-linear change up to follow-up week 3. [a]

Parameter	Measurement	z	P>|z|	95% confidence interval
**ACE2**	RASi discontinuation baseline	0.08	0.935	[-0.697; 0.758]
	RASi discontinuation week 2	1.14	0.255	[-0.344; 1.300]
	RASi discontinuation week 3	1.23	0.219	[-0.354; 1.542]
**ACE-S**	RASi discontinuation baseline	1.12	0.263	[-0.500; 1.830]
	RASi discontinuation week 2	0.17	0.868	[-0.736; 0.873]
	RASi discontinuation week 3	2.69	0.007	[0.360; 2.295]
**Ang 1-7**	RASi discontinuation baseline	-1.48	0.138	[-1.774; 0.246]
	RASi discontinuation week 2	-0.42	0.678	[-1.432; 0.930]
	RASi discontinuation week 3	0.20	0.838	[-1.230; 1.516]
**Ang 1-5**	RASi discontinuation baseline	0.77	0.441	[-0.646; 1.485]
	RASi discontinuation week 2	-0.18	0.859	[-1.340; 1.117]
	RASi discontinuation week 3	1.53	0.126	[-0.318; 2.579]
**Ang I**	RASi discontinuation baseline	-1.32	0.187	[-1.843; 0.360]
	RASi discontinuation week 2	-0.89	0.372	[-1.991; 0.745]
	RASi discontinuation week 3	-0.58	0.561	[-2.033; 1.104]
**Ang II**	RASi discontinuation baseline	1.12	0.263	[-0.427; 1.565]
	RASi discontinuation week 2	-0.87	0.382	[-1.795; 0.688]
	RASi discontinuation week 3	1.64	0.102	[-0.235; 2.623]
**PRA-S**	RASi discontinuation baseline	-0.75	0.450	[-1.417; 0.629]
	RASi discontinuation week 2	-0.81	0.418	[-1.787; 0.743]
	RASi discontinuation week 3	0.25	0.800	[-1.265; 1.641]

Abbreviations: ACE, angiotensin-converting enzyme; Ang, angiotensin; PRA-S, plasma renin activity; RAS, renin–angiotensin system; RASi, renin–angiotensin system inhibitor.

[a] Model: Mixed-effects regression model for RAS metabolites assuming a non-linear change over weeks using "RASi continuation" as reference group. As-treated analysis was performed. Absolute values are log-transformed and adjusted for baseline. PRA-S and ACE-S are reported in pM, and all other angiotensin levels are presented in pmol/L. The WHO scale (1-7) is unitless.

Table S9, related to Table 3 and Table S8: RAS metabolites in the biomarker groups stratified by mild (maximum WHO score 3-4) and severe (maximum WHO score 5–7) COVID-19 disease. [a]

RAS Metabolite	Biomarker group	Median (IQR)	P RASi continuation baseline vs. RASi continuation follow-up	P RASi discontinuation baseline vs. RASi discontinuation follow-up	P RASi continuation follow-up vs. RASi discontinuation follow-up	P mild vs. severe COVID-19
**ACE2**			0.028	<0.001	0.758	0.063
	RASi continuation baseline	2.33 (1.23–4.56)				
	RASi discontinuation baseline	2.25 (1.22–3.58)				
	RASi continuation follow-up	4.03 (3.23–6.09)				
	RASi discontinuation follow-up	3.61 (2.08–9.15)				
	Mild COVID-19	2.33 (1.77–4.22)				
	Severe COVID-19	4.63 (2.63–41.03)				
**ACE-S**			0.093	0.136	0.028	0.107
	RASi continuation baseline	0.29 (0.04–1.91)				
	RASi discontinuation baseline	1.36 (0.23–2.32)				
	RASi continuation follow-up	0.34 (0.06–1.93)				
	RASi discontinuation follow-up	1.58 (0.71–2.53)				
	Mild COVID-19	1.87 (0.18–2.28)				
	Severe COVID-19	0.91 (0.47–1.50)				
**Ang 1-7**			0.441	0.796	0.678	0.186

	RASi continuation baseline	12.15 (2.90–34.97)				
	RASi discontinuation baseline	12.01 (1.50–42.72)				
	RASi continuation follow-up	12.55 (7.22–36.19)				
	RASi discontinuation follow-up	6.26 (3.01–64.20)				
	Mild COVID-19	8.52 (4.60–17.64)				
	Severe COVID-19	27.33 (6.54–100.56)				
**Ang 1-5**			0.398	0.711	0.063	0.285
	RASi continuation baseline	2.97 (1.00–18.05)				
	RASi discontinuation baseline	14.06 (4.75–20.10)				
	RASi continuation follow-up	4.75 (1.00–10.15)				
	RASi discontinuation follow-up	9.66 (3.71–43.53)				
	Mild COVID-19	8.35 (2.46–17.55)				
	Severe COVID-19	10.21 (6.28–79.90)				
**Ang I**			0.575	0.084	0.435	0.544
	RASi continuation baseline	273.83 (70.31–648.66)				
	RASi discontinuation baseline	195.32 (35.79–530.56)				
	RASi continuation follow-up	190.80 (50.32–505.29)				
	RASi discontinuation follow-up	82.91 (31.38–294.32)				
	Mild COVID-19	97.49 (43.21–249.85)				
	Severe COVID-19	232.31 (25.44–432.50)				

**Ang II**			0.878	0.334	0.048	0.950
	RASi continuation baseline	21.36 (8.84–334.84)				
	RASi discontinuation baseline	170.09 (49.99–348.43)				
	RASi continuation follow-up	29.44 (13.34–75.45)				
	RASi discontinuation follow-up	119.14 (57.56–273.37)				
	Mild COVID-19	105.99 (14.75–230.49)				
	Severe COVID-19	87.33 (21.71–235.22)				
**PRA-S**			0.575	0.099	0.985	0.544
	RASi continuation baseline	524.12 (76.39–715.88)				
	RASi discontinuation baseline	422.80 (127.92–1066.58)				
	RASi continuation follow-up	302.56 (100.22–579.29)				
	RASi discontinuation follow-up	209.50 (78.68–747.62)				
	Mild COVID-19	256.82 (131.71–455.26)				
	Severe COVID-19	305.55 (91.54–836.88)				

Abbreviations: ACE, angiotensin-converting enzyme; Ang, angiotensin; COVID-19, coronavirus disease 2019; PRA-S, plasma renin activity; RAS, renin-angiotensin system; RASi, renin-angiotensin system inhibitor; WHO, World Health Organization.

[a] RAS metabolites are presented as median (IQR) measurements at baseline and median values over time per patient (follow-up) within the respective group. RASi continuation and RASi discontinuation were compared (p values) using the Wilcoxon test and Mann-Whitney U test; COVID-19 severity (mild versus severe) was compared using the Mann-Whitney U test. Absolute values were log-transformed for analyses. PRA-S and ACE-S are presented in pM, all other angiotensin values are reported in pmol/L.

Table S10, related to Figure 4: Effect of RASi continuation and discontinuation on RAS metabolites stratified by type of medication. [a]

RAS metabolite	ACEi		ARB		P	
	Stay on ACEi (n=5)	Stop ACEi (n=9)	Stay on ARB (n=5)	Stop ARB (n=11)	Stay on ACEi vs. stop ACEi	Stay on ARB vs. stop ARB
**ACE2**	3.55 (3.71)	4.34 (10.77)	3.50 (49.99)	2.33 (7.10)	0.386	0.533
**ACE-S**	0.05 (0.09)	0.52 (1.60)	1.87 (0.94)	1.77 (1.32)	0.009	0.865
**Ang 1-7**	13.22 (27.71)	7.85 (94.01)	8.51 (53.55)	9.62 (27.71)	0.641	0.909
**Ang 1-5**	1.00 (6.10)	9.25 (51.51)	13.06 (86.01)	17.33 (44.89)	0.027	0.821
**Ang I**	341.24 (288.83)	177.51 (391.91)	84.05 (707.09)	95.52 (193.56)	0.257	0.955
**Ang II**	14.45 (12.14)	131.61 (187.74)	116.14 (1324.82)	105.99 (390.39)	0.028	0.955
**PRA-S**	469.06 (449.56)	305.55 (591.07)	193.59 (2005.73)	248.68 (675.64)	0.641	0.955

Abbreviations: ACE, angiotensin-converting enzyme; ACEi, angiotensin-converting enzyme inhibitor; Ang, angiotensin; PRA-S, plasma renin activity; ARB, angiotensin receptor blocker; RAS, renin-angiotensin system; RASi, renin-angiotensin system inhibitor.

[a] Values are presented as total median (IQR) RAS measurements per patient over time within the respective group. Group comparisons (p values) were performed using the Mann-Whitney U test. Values were log-transformed for calculation. PRA-S and ACE-S are presented in pM, and all other angiotensin values are reported in pmol/L.

Supplementary figures:

Figure S1, related to Table 2 or Table S2: Weekly course of the WHO score (1–7) for each clinical treatment group. [a]

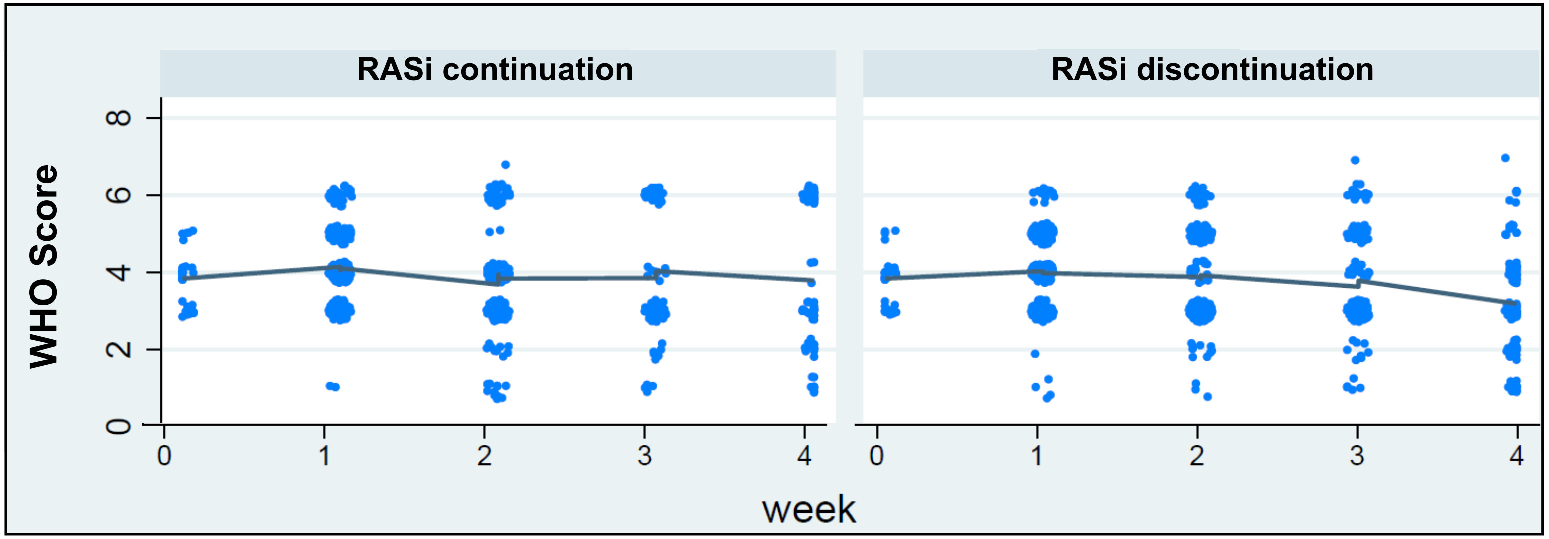

Abbreviations: RAS, renin-angiotensin system; RASi, renin-angiotensin system inhibitor; WHO, World Health Organization.

[a] Model: Intention-to-treat analysis was performed applying the mixed linear regression model accounting for repeated measurements assuming a non-linear change over time (weeks).

Figure S2, related to Table 2: Inverse kaplan–meier plot of median time (days) to national early warning score ≤ 2 or discharge from baseline between the RASi continuation and discontinuation groups.

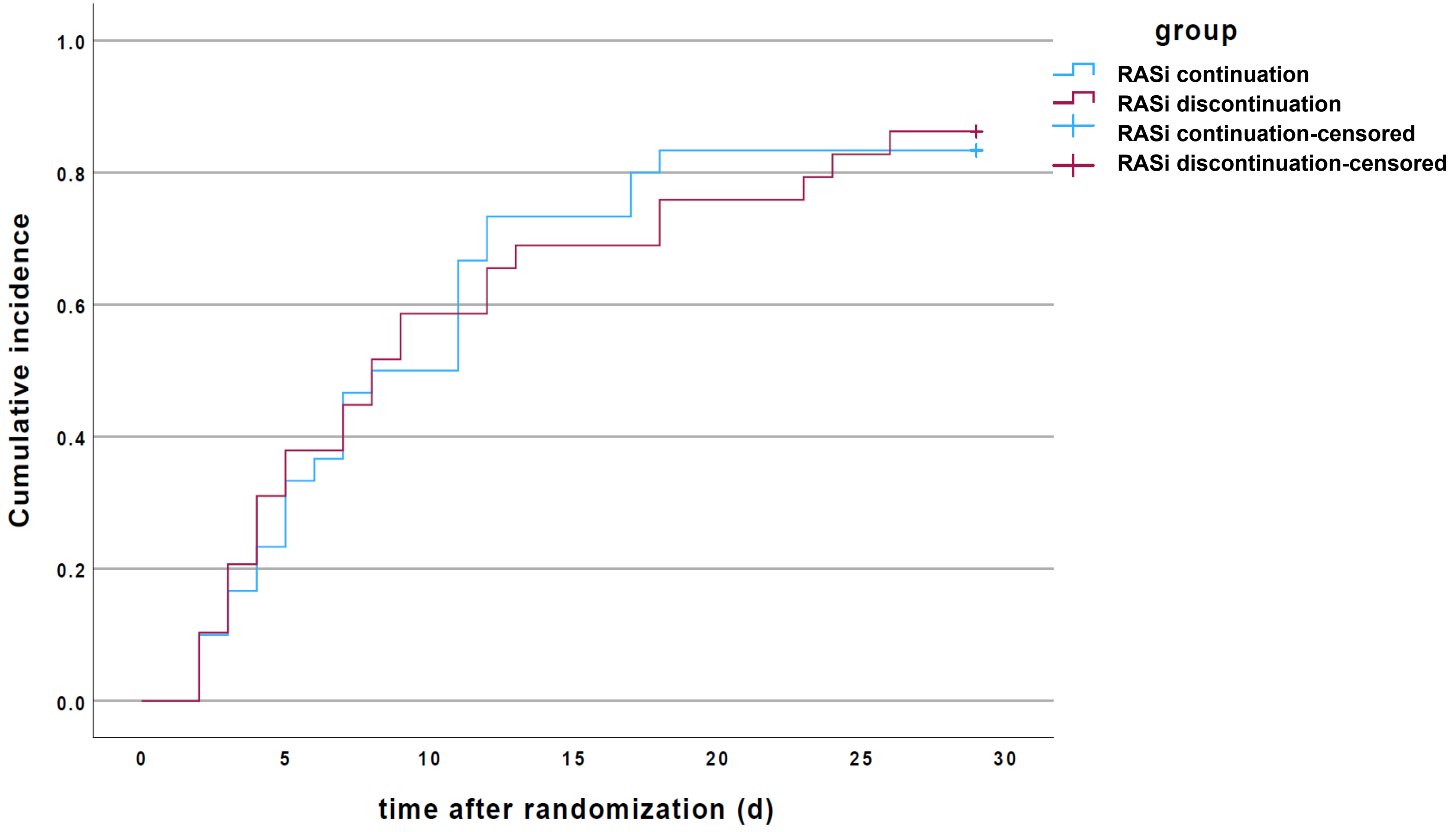

Abbreviation: RASi, renin-angiotensin system inhibitor.

Figure S3, related to Table 2 or Table S5: Blood pressure and glomerular filtration rate over time between RASi continuation and discontinuation in the clinical treatment group analysis. A.) Median systolic blood pressure. B.) Median glomerular filtration rate.

A

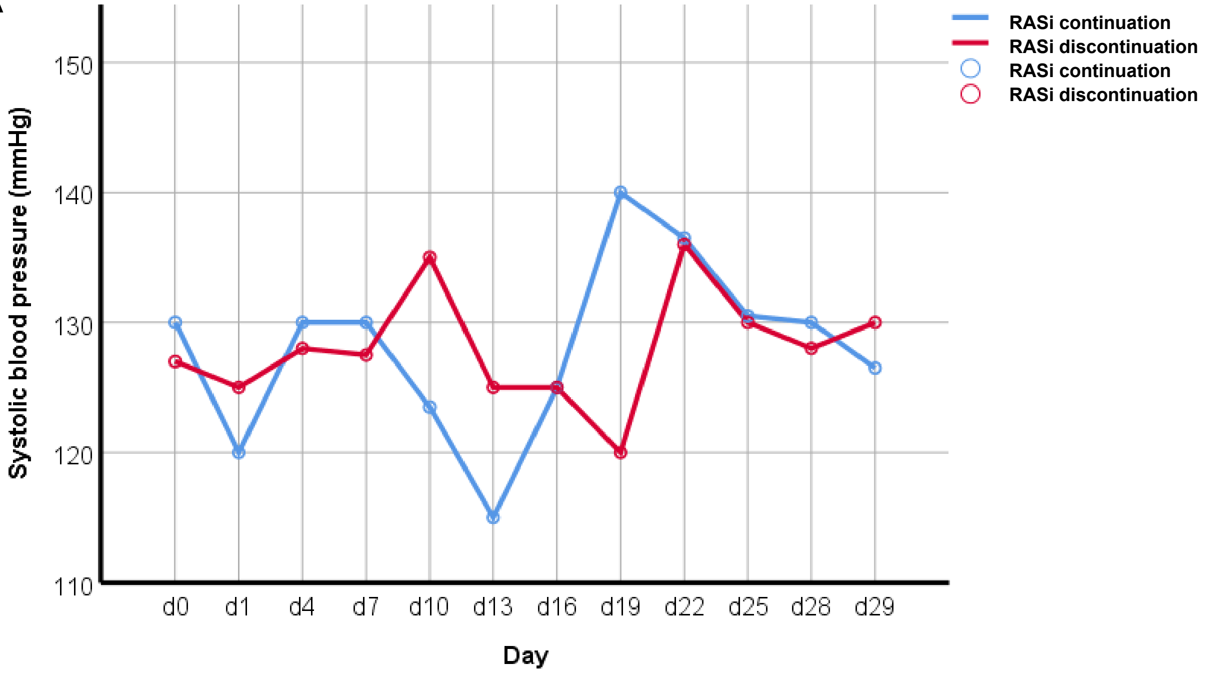

B

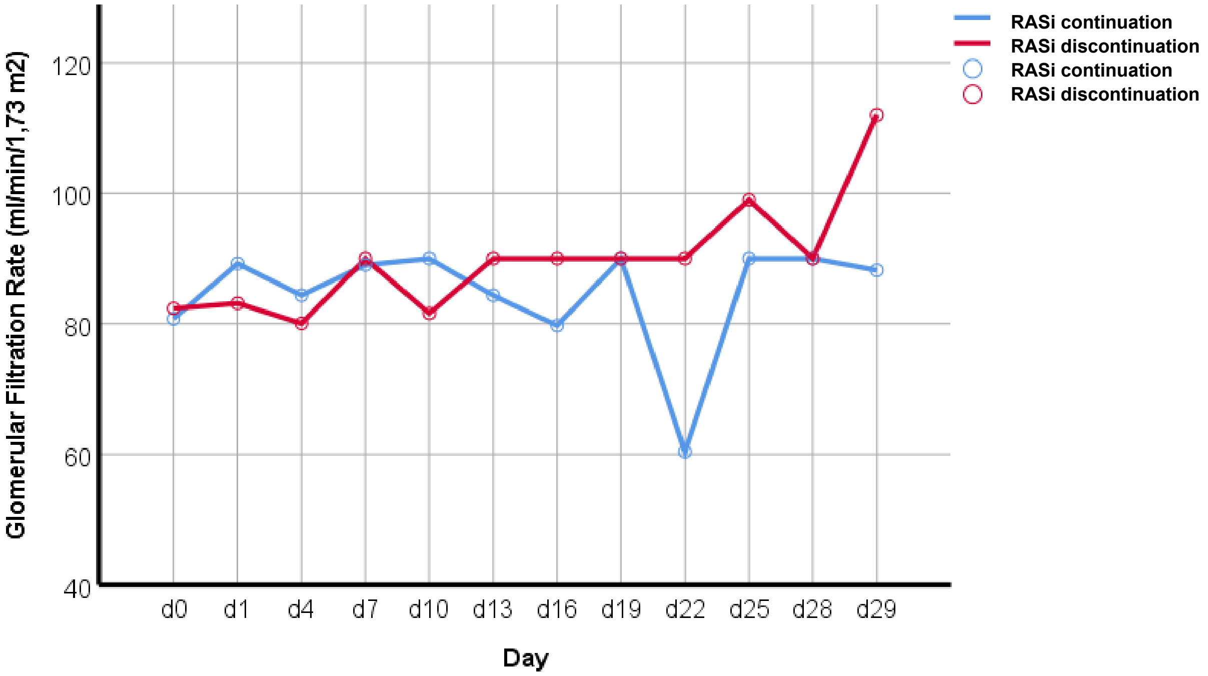

Abbreviation: RASi, renin–angiotensin system inhibitor.

Figure S4, related to Figure 2 and Table S6: Allocation of non-substudy B patients with arterial hypertension and RAS-inhibitory treatment randomized to ACOVACT.

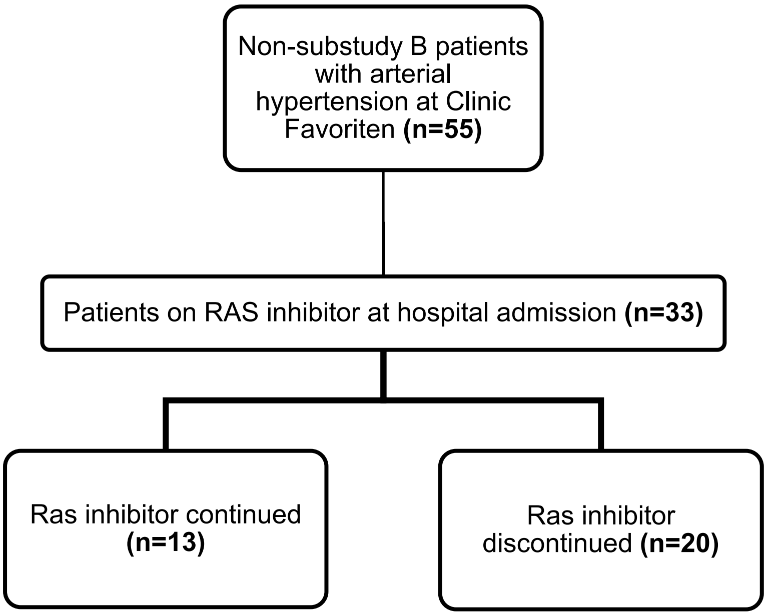

Abbreviations: ACOVACT, Austrian Corona Virus Adaptive Clinical Trial; RAS, renin-angiotensin system.

Adverse events: Adverse events listed for the two main treatment groups. [a]

Adverse event	RASi continuation (n=30)	RASi discontinuation (n=29)	P
Minimum 1 clinically mild adverse event per patient	21	23	0.412
Minimum 1 clinically moderate adverse event per patient	16	15	0.902
Minimum 1 clinically severe adverse event per patient	5	3	0.478
Total clinically mild adverse events	69	81	-
Total clinically moderate adverse events	61	37	-
Total clinically severe adverse events	17	4	-
Acute kidney failure	4	4	-
Hyperkalemia	1	2	-
Hypokalemia	2	3	-
Hypertensive episode	1	3	-
Hypotensive episode	2	2	-
Hepatic enzymes increased	9	9	-
Diarrhea	5	3	-
Colitis	1	0	-
Bleeding event	2	1	-
Anemia	5	1	-
Bradycardia	3	1	-
Tachycardia	1	0	-
Atrial fibrillation	2	2	-
Left ventricular dysfunction	1	0	-
Coronary artery stent implantation	0	1	-
Edema	5	3	-
Pleural effusion	2	0	-
Bacterial infection	5	7	-
Mycotic infection	3	1	-
Hypernatremia	1	0	-
Hyponatremia	1	0	-
Hypophosphatemia	3	0	-

Abbreviation: RASi, renin–angiotensin system inhibitor.

[a] Group comparisons were performed using the chi square or Fisher's exact test, depending on sample size.

Serious adverse events: Serious adverse events listed for the two main treatment groups. [a]

Serious adverse event	RASi continuation (n=30)	RASi discontinuation (n=29)	P
Minimum 1 serious adverse event per patient	6	7	0.701
Total serious adverse events	10	9	0.850
Fatal serious adverse events	4	2	0.413
Respiratory failure	6	3	-
Lung bleeding	1	0	-
multiorgan failure	1	1	-
Incomplete paraplegic syndrome	0	1	-
Acute polyneuropathy	0	1	-
Superinfection	0	1	-
Malignant hyperthermia	0	1	-
Fournier's gangrene	0	1	-
Generalized tonic-clonic seizure	1	0	-
Delirium	1	0	-

Abbreviation: RASi, renin–angiotensin system inhibitor.

[a] Group comparisons were performed using the chi square or Fisher’s exact test, depending on sample size.
