# Supplementary material for: Renin-angiotensin system inhibitor discontinuation in COVID-19 did not modify systemic ACE2 in a randomized controlled trial
Source: iScience. 2023 Oct 5;26(11):108146. doi: 10.1016/j.isci.2023.108146 (PMC10585392; doi:10.1016/j.isci.2023.108146)
Supplement: Document S2. ACOVACT study protocol [file mmc2.pdf]

Clinical Study Protocol

A multicenter, randomized, active controlled, open label, platform trial on the efficacy and safety of experimental therapeutics for patients with COVID-19 (caused by infection with severe acute respiratory syndrome coronavirus-2)

ACOVACT (*Austrian CoronaVirus Adaptive Clinical Trial)*

EUDRACT-NR: 2020-001302-30

3.1 / 10.12.2020

Confidentiality Statement

The information contained in this document, especially unpublished data, is the property of the sponsor of this study. It is therefore provided to you in confidence as an Investigator, potential Investigator, or consultant, for review by you, your staff, and an Independent Ethics Committee or Institutional Review Board. It is understood that this information will not be disclosed to others without written authorization from the principal investigator, except to the extent necessary to obtain informed consent from those persons to whom the study drug may be administered.

**Test drug (IMP) and Pharmaceutical Company**	**"ANTIVIRAL THERAPY"**<br><br>Hydroxychloroquine (Sanofi Aventis) (Inactive)*<br><br>Lopinavir/Ritonavir (Abbvie)<br><br>Remdesivir (Gilead)<br><br>Rivaroxaban (Bayer)<br><br>RAS blockade: Candesartan (Takeda), [Nitrendipin (Bayer), doxazosin (Gerot Lannach) as control]<br><br>Asunercept (Apogenix AG)<br><br>Pentaglobin (Biotest Pharma GmbH)<br><br>*treatment arm not active anymore due to safety concerns
**Protocol author**	Christian Schörgenhofer
**Investigator**	Bernd Jilma
**Document type**	Clinical study protocol
**Study phase**	II
**Document status**	final
**Date**	10.12.2020
**Number of pages**	92

1 SPONSOR, INVESTIGATOR, MONITOR AND SIGNATURES

Sponsor/or representative (OEL) (AMG §§ 2a, 31, 32)

Assoc. Prof. Priv. Doz. Dr. Markus Zeitlinger, Department of Clinical Pharmacology Medical University of Vienna, Austria

______________________________ ________________________

Signature (OEL) Date

Investigator (AMG §§ 2a, 35, 36)

Univ.-Prof. Dr. Bernd Jilma, Department of Clinical Pharmacology, Medical University of Vienna, Austria

______________________________ ________________________

Signature Date

Monitor (AMG §§ 2a, 33, 34)

Univ.-Prof. Dr. med. univ. Michael Wolzt, Clinical Trials Coordination Centre, Medical University of Vienna, Austria

______________________________ ________________________

Signature Date

Statistician

Assoc. Prof. Dr. Franz König, Center for Medical Statistics, Informatics and Intelligent Systems, Medical University of Vienna, Austria

______________________________ ________________________

Signature Date

2 PROTOCOL SYNOPSIS

TITLE	A multicenter, randomized, active controlled, open label, platform trial on the efficacy and safety of experimental therapeutics for patients with COVID-19 (caused by infection with severe acute respiratory syndrome coronavirus-2)
OBJECTIVES	**Primary Objective**<br>• To investigate the efficacy of various experimental therapeutics for patients with severe acute respiratory syndrome coronavirus 2 (SARS-CoV-2); for efficacy assessment a 7 category ordinal scale for clinical severity assessment as proposed by the World Health Organization will be used:<br>▪ Time to sustained improvement of one category from admission<br><br>**Secondary Objectives**<br>• To assess efficacy according to the National Early Warning Score (NEWS):<br>▪ Time to discharge or to a NEWS of ≤2 and maintained for 24 hours, whichever occurs first<br>▪ Change from baseline (once daily)<br>• Oxygenation<br>▪ Oxygenation free days until day 29<br>▪ Incidence and duration of new oxygen use during the trial<br>• Mechanical Ventilation<br>▪ Ventilator free days until day 29<br>▪ Incidence and duration of new mechanical ventilation use during the trial<br>• Viral load/viral clearance<br>▪ Baseline, then three times a week<br>• Hospitalization<br>▪ Duration of hospitalization<br>▪ Duration of intensive care unit treatment<br>▪ Intensive care unit admissions<br>• Mortality<br>▪ 15-day, 29-day, 60-day, 90-day mortality<br>• Sub-Study A: number of thromboembolic events<br>• Renin-Angiotensin System (RAS)- and bradykinin fingerprint at baseline and at least once weekly (on days 7±1, 14±1, 21±1, 28±1, through recovery), depending on whether patients are hospitalized with SARS-CoV-2 infection, or treated as outpatients (the latter applies only to patients in sub-study B). RAS- and bradykinin

	fingerprint analysis will depend on availability of samples and may therefore only be performed in a subgroup of patients.<br>• Within all patients, the impact of obesity and associated diseases on mortality will be investigated (e.g. mortality, inflammatory response, duration of hospitalization, intensive care unit admission, new oxygen use, duration of oxygen)<br>• Exploratory assessment of transaminases (including alkaline phosphatase, gamma-glutamyltransferases (GGT), aspartate aminotransferase (AST), alanine aminotransferase (ALT)) and liver function parameters (including bilirubine, prothrombin time, international normalized ratio, albumin, fibrinogen) and their course during the disease and treatment<br>• An exploratory endpoint will encompass a comprehensive assessment of inflammatory parameters and their changes during treatment and washout time, as well as exploratory genotype and RNA analysis with a focus on inflammation, coagulation, and the specific pathophysiology of the disease (if possible for center).<br>• Pharmacokinetics of antiviral substances, if assays are available (e.g lopinavir/ritonavir trough level analysis)<br><br><u>Sub-Study B</u><br><br>• Blood pressure (in mmHg, self-reported in outpatients)<br>• Dyspnea (NYHA classification; self-reported in outpatients)<br>• Body temperature (in°C, self-reported in outpatients)<br>• Quality of life: EuroQoL- 5 Dimension (EQ-5D)<br>• Activities of daily living/life participation, Instrumental activities of daily living scale (IADLS)<br>• PROMIS social function measures<br>• Dizziness<br>• Gastrointestinal symptoms including nausea and vomiting (PROMIS gastrointestinal symptom scales)<br>• Gastrointestinal symptom rating scale (before respiratory symptoms, many patients with COVID-19 had diarrhea, nausea, vomiting, abdominal discomfort)<br>• Headache: headache impact test 6-item (HIT-6)<br>• Anxiety: hospital anxiety and depression scale (HADS)<br>• Fear/anxiety reported with SARS-CoV-2 infection (qualitative interview at baseline and recovery, GAD-7 scale value at all study visits [telephone or in person])<br>Safety<br>▪ Cumulative incidence of serious adverse events

<table>
<tr><td></td><td>Discontinuation or temporary suspension of therapy
Changes in white cell count, hemoglobin, platelets, creatinine, glucose, total bilirubin, alanine aminotransferase (ALT), aspartate aminotransferase (AST) over time
Sub-study C: modified SOFA score, paO2/FiO2 ratio, or SpO2/FiO2 ratio</td></tr>
<tr><td>DESIGN / PHASE</td><td>Main “antiviral therapy” study: Prospective, multicenter, randomized, open label, active controlled, platform, phase II trial. It is expected that different centers have variable access to experimental treatments and each center will randomize between open arms.
Substudies will be performed permitting further randomization to
rivaroxaban vs. standard of care,
angiotensin receptor blocker therapy vs. alternative anti-hypertensives
asunercept (at three different doses) vs. best standard of care and
Pentaglobin vs best standard of care</td></tr>
<tr><td>STUDY PLANNED DURATION</td><td><table><tr><td>First patient First visit</td><td>2Q/ 2020</td><td>Last patient First visit</td><td>2Q/ 2021</td><td>Last patient Last visit</td><td>3Q/ 2021</td></tr></table></td></tr>
<tr><td>CENTER(S)
/ COUNTRY(IES)</td><td>≥2 centers in 1 country.
Austria</td></tr>
<tr><td>PATIENTS / GROUPS</td><td>For the main “antiviral therapy” study currently 4 treatment arms are planned. Treatment arm 4 refers to standard of care (SOC) and will be the “control arm” of the study. A preliminary sample size calculation has been performed for the comparison of two groups based on a recent publication.
Therefore, we plan for n=100 patients per treatment arm in the main study. This number may increase after the interim analysis. This means that currently the main study is planned for approximately N=500 patients.

After inclusion of 20 and 50 patients per arm, interim analyses will be performed to draw conclusions on the sample size and to stop trial arms for safety or futility.

An even randomization ratio for “antiviral” arms (1:1:1:1) is theoretically intended but a dynamic assignment will occur according to available and permissible treatments (i.e. where no absolute contra-indication exists).</td></tr>
</table>

	**Sub-Study A Anticoagulation**: Patients with eGFR of >20 mL/min will be randomized to treatment with **rivaroxaban** or standard of care which likely includes prophylactic doses of low molecular weight heparins.<br><br>**Sub-Study B RAS Blockade**: Patients with **blood pressure ≥130/85mmHg** (or established and treated arterial hypertension) may be included into a sub-study labeled as B. The decision whether to include patients also in sub-study B will be taken within the first 24 hours after randomization to the main study. Two consecutive blood pressure measurements are a prerequisite for randomization.<br>Patients in the sub-study B will be randomized to two groups with different antihypertensive treatment: RAS-blocker containing treatment (candesartan) and non-RAS-blocker containing treatment (e.g. nitrendipine, doxazosin).<br>We expect that about 1/3 of patients in the main part might also be included in sub-study B.<br>Additionally, two control groups will be included: control group 1 will consist of patients with suspected COVID-19 infection, who test negatively for SARS-COV-2 infection (n=10) and healthy volunteers with negative tests for SARS-COV-2 (n=10)<br><br>**Sub-Study C Anti-inflammatory/Immunomodulatory treatment**: patients may be eligible for experimental anti-inflammatory treatment. In short, in this randomized sub-study hospitalized patients with clinical or radiological signs of pneumonia and oxygen demand may be included. These patients are at increased risk to develop a severe cause or fatal disease. Thus, they may receive asunercept, a fully human CD95L-binding protein, or pentaglobin, IgM enriched immunoglobulins. This part of the study will be randomized against standard of care.<br>This part of the study may include patients who were also included into sub study A and/or B.<br>For the asunercept part of sub-study C, four treatment groups will be available: one control arm (standard of care) and three different dose groups (25mg, 100 mg and 400 mg per week i.v.). randomization will be carried out in 1:3 ratio. Initially, inclusion of 100 patients is planned, although, the final sample size will be determined during interim analyses (see detailed information in the statistical analysis part).<br>For the Pentaglobin part of sub-study C the primary objective is the inclusion of patients with uni-/bilateral pneumonia, elevated C-reactive protein levels (>5 mg/dl) and respiratory deterioration in danger of sepsis or acute respiratory distress syndrome (ARDS) who are admitted to an ICU.

	Importantly, randomization to only one of the mentioned treatments will be possible (if the substances are available in the respective center). This means, a patient may not receive two of the mentioned substances, e.g. asunercept and pentaglobin.
INCLUSION CRITERIA	• Laboratory confirmed (i.e. PCR-based assay) infection with SARS-CoV-2 (ideally but not necessarily ≤72 hours before randomization for "antiviral" treatments) OR radiological signs of COVID-19 in chest X-ray or computed tomography*<br>• Hospitalisation due to SARS-CoV-2 infection (for anti-viral treatment arms)<br>• Requirement of oxygen support (due to oxygen saturation <94% on ambient air or >3% drop in case of chronic obstructive lung disease)<br>• Informed Consent obtained, the patient understands and agrees to comply with the planned study procedures, except for sub-study C: obtaining informed consent may be impossible due to the severe condition of the patient and may be waived<br>• ≥18 years of age<br>• For female patients with childbearing potential: willingness to perform effective measures of contraception during the study.<br>• Sub-study A: eGFR of >20 mL/min<br>• Sub-study B: outpatients with COVID-19 may be included<br>• Sub-study B: blood pressure ≥130/85mmHg in 2 consecutive measurements OR patients with established and treated hypertension<br>• Sub-study B: Control group 1: Patients with suspicion of but negative tests for COVID-19. This group may consist of hospitalized and non-hospitalized patients.<br>• Sub-study B: control group 2: healthy volunteers<br>• Sub-study C: Signs of respiratory deterioration and progressing inflammation: need for oxygen supplementation, non-invasive ventilation, high-flow oxygen devices or mechanical ventilation AND CRP levels >5mg/dL (for Pentaglobin only), and admission to an ICU (for Pentaglobin only).<br>If for any given reason a patient does not qualify to participate in the main study, this will not preclude participation in sub-study C.<br>*In case of negative PCR but clear radiological signs of COVID-19 patients have to be retested with serial nasopharyngeal swabs and PCR and, if possible antibody based assays. In any case, a laboratory based proof of COVID-19 is required or else the subject may be excluded from the per protocol analysis.
EXCLUSION CRITERIA	• Moribund or estimated life expectancy <1 month (e.g. terminal cancer, etc.)<br>• Patient does not qualify for intensive care, based on local triage criteria

	• Pregnancy or breastfeeding<br>• Severe liver dysfunction (e.g. ALT/AST > 5 times upper limit of normal)<br>• Stage 4 chronic kidney disease or requiring dialysis for direct anticoagulant treatment<br>• Allergy or intolerances to any of the experimental substances -> exclusion for the respective treatment arm; for asunercept known hereditary fructose intolerance<br>• Anticipated discharge of hospital within 48 hours (for anti-viral treatment arms)<br>• Contraindications treatment arm 2 (lopinavir/ritonavir): severe hepatic impairment, CYP3A4/5 metabolized drugs as deemed relevant by treating physicians, HIV positive<br>• Contraindication treatment arm 3 (remdesivir): Bodyweight <40kg,<br>• Sub-study A Contraindications: active bleeding or bleeding diathesis, lesion or condition considered as major risk factor for bleeding, recent brain or spinal injury, recent brain or spinal or ophthalmic surgery, recent intracranial hemorrhage, known or suspected esophageal varices, arteriovenous malformations, vascular aneurysms, major intraspinal or intracerebral vascular abnormalities.<br>• Sub-study A: ongoing therapeutic anticoagulation, which will continue, according to clinical practice<br>• Sub-study B Contraindications chronic heart failure, allergies, hypersensitivities and intolerances, severe hepatic impairment and/or cholestasis, concomitant therapy with aliskiren-containing medications (for patients with diabetes mellitus or a GFR<60ml/min/1.73m^2), known significant bilateral renal artery stenosis or renal artery stenosis of a solitary kidney<br>• Sub-study B: Control group 1: with or without RAS blockers, Control group 2: Healthy volunteers: concomitant medication with RAS-blockers<br>• Sub-study C: known active HIV or viral hepatitis<br>• Asunercept: females of childbearing potential<br>• Sub-Study C: Known active tuberculosis.
STUDY PERIODS	This is a multicenter, randomized, open label, controlled platform trial and new treatment arms may be opened, while futile therapy arms may be closed, depending on the availability of new treatments, relevant publications of new results and our own results.

INVESTIGATIONAL DRUG	**Treatment arm 1:** Hydroxychloroquine 200mg 2-0-2 on day 1 followed by 200mg 1-0-1 for 7 days (recommended, or up to maximum dose of 600mg or 200mg 1-1-1 where standard practice)<br>**THIS TREATMENT ARM IS INACTIVE**<br>**Treatment arm 2:** Lopinavir/Ritonavir 200mg/50mg 4-0-4 loading dose, followed by 3-0-3 maintenance dose<br>**Treatment arm 3:** Remdesivir 200mg on day 1, 100mg/day for ≥4 further days (total treatment ≥5 days, according to locas standard)<br>**Treatment arm 4:** standard of care (SOC)<br>**Sub-study A:** Rivaroxaban as anticoagulant 10 mg ½-0-½ (or 2.5mg 2-0-2, as available) vs standard of care thromboprophylaxis with low molecular weight heparins (e.g. enoxaparin 40mg)<br>**Sub-study B:** Candesartan as RAS blocking agent vs standard of care (e.g. nitrendipine, amlodipine or doxazosin as control agents (depending on concomitant medication and comorbidities), dose titrated to normotension.<br>**Sub-study C:**<br>• Asunercept, administered once weekly intravenously at 25mg, 100mg or 400mg<br>• Pentaglobin, continuous IV application over 12h of a total dose of 7ml/kg/day for 5 days, provided in a 50mg/ml solution

COMPARATIVE DRUG /CONTROL CONDITION	**Main Study:**<br>Different "antiviral" treatment arms will be tested against each other and against SOC<br>**Sub-study A:**<br>Randomization will be carried out against standard of care which will likely include prophylactic doses of low molecular weight heparin.<br>**Sub-study B:**<br>randomized switch of patients with previously known and treated hypertension to non-RAS blocking agents (standard of care, e.g. nitrendipine, amlodipine or doxazosin) versus "stay-on-any-RAS-blockade", or de novo treatment of patients with blood pressure >130/85mmHg in two consecutive measurements with RAS blocking agent (candesartan) versus non-RAS-blocking agents (e.g. nitrendipine, amlodipine or doxazosin), or no treatment, if blood pressure <140/90mmHg. RAS blockers will be compared vs. non-RAS blocking antihypertensive agents.<br>Healthy volunteers as another control group<br>**Sub-study C:**<br>**Asunercept**: Randomization will be done between groups receiving Asunercept at different dose levels (group 1: 25 mg; group 2: 100 mg; group 3: 400 mg) and best standard of care. Thus, there will be a 3:1 randomization between patients receiving *verum* and control patients. In case of a safety signal at interim analyses at a given dose, the arm will be terminated and patients will be switched to the remaining dose(s) which is/are tolerated. In case differences in efficacy are seen at interim analyses, patients will be switched from the less effective dose(s) to the more effective dose(s)or to equally effective lower dose(s)<br>**Pentaglobin**: Randomisation will be carried out against best standard of care (without IVIG or IL-6 blockage).

CONCOMITANT MEDICATION	**Allowed**<br>Other treatment will not be affected by this trial, but the control groups should not be contaminated with similar drugs (e.g. no direct anticoagulants (apixaban, dabigatran, edoxaban) in sub-study A, other angiotensin converting enzyme inhibitors or angiotensin receptor blockers in sub-study B),<br>**Special caution:**<br>Concomitant medication of hydroxychloroquine with other QTc prolonging agents (e.g. metoclopramide, ondansetron, (es)citalopram, haloperidol, sotalol, flecainide, domperidone, azithromycin, clarithromycin, tacrolimus, vardenafil, fluconazole, ketoconazole, lithium) should only be used with special precautions. Serial electrocardiograms with QTc monitoring are warranted.<br><br>**Not allowed**<br>Contraindications implicated by the respective treatment, e.g. relevant CYP3A4 dependent substances (according to investigator judgement) and Ritonavir<br>Asunercept: Specific immunotherapy in the control group (e.g. IL-6 blockers, TNF-blockers, immune checkpoint blockers), unless standard of care
EFFICACY ENDPOINTS	The primary endpoint is time to clinical improvement, which is defined as time from randomization to a sustained improvement of at least one category on two consecutive days compared to the status at randomization measured on a seven-category ordinal scale (proposed by WHO).<br><br>The 7-categories of the World Health Organization proposed scale, as follows:<br>1. Not hospitalized, no limitations on activities<br>2. Not hospitalized, limitation on activities;<br>3. Hospitalized, not requiring supplemental oxygen;<br>4. Hospitalized, requiring supplemental oxygen;<br>5. Hospitalized, on non-invasive ventilation or high flow oxygen devices;<br>6. Hospitalized, on invasive mechanical ventilation or ECMO;<br>7. Death.<br>▪ Time to sustained (i.e. >48h) improvement of one category from admission<br>▪ Clinical status on a daily basis<br>▪ Mean change in the ranking on an ordinal scale from baseline (assessment once daily)<br>- NEWS

	▪ Time to discharge or to a NEWS of ≤2 and maintained for 24 hours, whichever occurs first<br>▪ Change from baseline (assessment once daily)<br>- Oxygenation<br>▪ Oxygenation free days until day 29<br>▪ Incidence and duration of new oxygen use (e.g. oxygen insufflation, high-flow oxygen, non-invasive ventilation, mechanical ventilation, etc.) during the trial<br>- Viral clearance<br>- Duration of hospitalization, of ICU treatment and number of ICU admissions<br>- Mortality at day 15, 29, day 60 and day 90<br>- Sub-Study A: number of thromboembolic events<br>- Sub-study B: To investigate the RAS and bradykinin fingerprint of patients with SARS-CoV-2 infection, with and without RAS blocking treatment, as specified above; to investigate blood pressure, dyspnea, body temperature, fear/anxiety (subject to availability of samples)<br>- Within all patients, the impact of obesity and associated diseases on mortality will be investigated (e.g. mortality, inflammatory response, duration of hospitalization, intensive care unit admission, new oxygen use, duration of oxygen)<br>- Exploratory assessment of transaminases (including alkaline phosphatase, gamma-glutamyltransferases (GGT), aspartate aminotransferase (ASAT), alanine aminotransferase (ALAT)) and liver function parameters (including bilirubin, prothrombin time, international normalized ratio, albumin, fibrinogen) and their course during the disease and treatment<br>- An exploratory endpoint will encompass a comprehensive assessment of inflammatory parameters and their changes during treatment and wash out time, as well as exploratory genotype and RNA analysis with a focus on inflammation, coagulation, and the specific pathophysiology of the disease (if possible for center).<br>- Sub-study C: modified SOFA score, paO_2/FiO_2 ratio, or SpO_2/FiO_2 ratio<br>- Substudy C, Pentaglobin part: CRP, PCT, IL-6, differential blood count (baseline and day 2, 3, 7), IgM, IgA and IgG levels (baseline, day 2, 3, 4, 5), modified SOFA score (baseline, day 7 and day 28)

TOLERABILITY / SAFETY ENDPOINTS	• Cumulative incidence of serious adverse events<br>• Discontinuation or temporary suspension of therapy<br>• Changes in white cell count, hemoglobin, platelets, creatinine, glucose, total bilirubin, alanine aminotransferase (ALT), aspartate aminotransferase (AST), uric acid over time<br>• Occurrence of drug induced liver injury (DILI) in various treatment arms<br>• Sub-study A: bleeding events
PHARMACOKINETIC / PHARMACODYNAMIC ENDPOINTS	Within all patients randomized to Lopinavir/Ritonavir treatment a drug-drug interaction check will be performed in a retrospective manner. However, if questions concerning CYP interactions or QTc prolonging agents occur during the trial, a drug-drug-interaction check will be possible after contact with the Department of Clinical Pharmacology.<br><br>- Pharmacokinetics of antiviral treatment (if assays are available, e.g. lopinavir/ritonavir)
QUALITY OF LIFE / PHARMACOECONOMIC ENDPOINTS	Substudy B, as specified above.
STATISTICAL METHODOLOGY	**Primary Endpoint**<br>Time to sustained improvement (i.e. >48h) of one category from admission in the 7-point clinical performance scale, which will be measured daily till day 29<br>**Null and alternative hypotheses:**<br>H_0 There is not difference between the treatment arms with regards to the primary endpoint<br>H_1: There is a significant difference between treatment arms with regards to the primary efficacy endpoint<br>**Sample size calculation**<br>As the initial phase of the main study will start with not all treatment arms, the initial sample size calculation is based on the comparison of two treatment arms. A sample size of 100 per treatment arm will ensure a power of >80% to detect an improvement of 6 days in the primary endpoint time to clinical improvement, if the median time in one group is 16 days.<br>Based on the recent publication of Cao et al. (1) we assumed that the median time in the treatment arm 2 (Lopinavir/Ritonavir) is about 16 days. Assuming an improvement between either two groups of 6 days in the median time to clinical improvement, a log-rank test at a two-sided significance level of $\alpha = 0.05$ with a sample size of 100 per treatment group would yield a power larger >80%. These assumptions would translate in a hazard ratio of 1.6 when assuming exponential time-to-event curves.

	There is currently only limited or no data available on expectable effect sizes. Therefore, we plan an interim analysis as soon as 20 and 50 patients have been randomized to the respective treatment arms. Study arms may be closed at interim analyses and after the second interim analysis, a sample size recalculation will be performed.<br><br>In short, n=100 per group for the asunercept (in total 400 patients) will be included. For the Pentaglobin part, n=20 will be included.<br>**Statistical methodology**<br>To test whether there is a difference between treatment arms, a log rank test will be performed at two-sided level alpha of 5%. The time-to-event data will be visualized by Kaplan-Meier plots.<br>Interim analysis<br>After inclusion of 20 and 50 patients per treatment arm.<br>The first interim analysis is mainly for safety and futility only. In the second interim analysis a sample size reassessment will be performed. To allow for early stopping in case overwhelming effects are observed, an alpha spending function with O´Brien and Fleming (OF) boundaries will be used.<br>For all sub-studies detailed statistical analysis plans will be created, which will also provide detailed information on sample size calculation.<br><br>Treatment arm four will only be analyzed in a descriptive manner.

3 LIST OF ABBREVATIONS

ADR	Adverse Drug Reaction
ACE-I	Angiotensin Converting Enzyme inhibitor
ARB	Angiotensin II Receptor Blocker
AE	Adverse Event
ALT	Alanin Aminotransferase
ARDS	Acute Respiratory Distress Syndrome
ATP	Adenosine Tri-Phosphate
AST	Aspartate Aminotransferase
BP	Blood Pressure
CRF	Case Report Form
CRO	Clinical Research Organization
CSR	Clinical Study Report
DILI	Drug induced liver injury
DOH	Declaration of Helsinki
DSUR	Development Safety Update Report
ECG	Electrocardiography
EOS	End of Study
EU	European Union
GCP	Good Clinical Practice
GGT	Gamma-Glutamyltransferase
HBsAg	Hepatitis B Surface Antigen
HBV	Hepatitis B Virus
HCG	Human Corionic Gonadotropin
HVC	Hepatitis C Virus
HIV	Human Immunodeficiency Virus
ICH	International Conference on Harmonization
IEC	Independent Ethics Committee
IMP	Investigational Medicinal Product
ISF	Investigator Site File
ISO	International Standardisation Organization
KKS	Koordinationszentrum für Klinische Studien
LLT	Lower level terms
mm	Millimeter
mmol/l	Millimol/Liter
RBC	Red Blood Cells
SAE	Serious Adverse Event
SAP	Statistical analysis plan
SAR	Serious Adverse Reaction
SOP	Standard Operating Procedure
SUSAR	Suspected Unexpected Serious Adverse Reaction
TMF	Trial Master File
TRAIL	Tumor necrosis factor related apoptosis inducing ligan

WBC	White Blood Cells
WHO	World Health Organization

4 TABLE OF CONTENTS

1 SPONSOR, INVESTIGATOR, MONITOR AND SIGNATURES 3

2 PROTOCOL SYNOPSIS 4

3 LIST OF ABBREVATIONS 16

4 TABLE OF CONTENTS 18

5 BACKGROUND INFORMATION 23
5.1 Background 23
5.2 Study rationale 27

6 STUDY OBJECTIVES (HYPOTHESES) 31
6.1 Primary objective (Hypothesis) 31
6.2 Secondary objectives (Hypothesis) 31

7 STUDY DESIGN 33
7.1 Study population 34
7.1.1 Subject population 34
7.1.2 Inclusion criteria 35
7.1.3 Exclusion criteria 36
7.1.4 Females of childbearing potential 36
7.1.5 Study duration 36
7.1.6 Withdrawal and replacement of subjects 37
7.1.7 Premature termination of the study 37

8 METHODOLOGY 38
8.1 Study medication 38
8.1.1 Dosage and administration 39
8.1.2 Study-drug up- and down titration 41
8.1.3 Study drug interruption or discontinuation 41
8.1.4 Study drug premature permanent discontinuation 41
8.1.5 Study-drug delivery & drug storage conditions 42
8.1.6 Study drug packaging and labeling 42
8.1.7 IMP administration & handling 42
8.1.8 Drug accountability 42
8.1.9 Procedures to assess subjects compliance 42

8.1.10 Concomitant medication 42
8.2 Randomization and stratification 43
8.3 Blinding 43
8.3.1 Emergency procedure for unblinding 44
8.3.2 Unblinding at the end of the study 44
8.4 Benefit and risk assessment 44
8.5 Study procedures 46
8.5.1 General rules for trial procedures 46
8.5.2 Screening investigation 46
8.5.3 Treatment phase 47
8.5.4 End-of-study (EOS) examination 48
8.5.5 Laboratory tests 48
8.5.5.1 Laboratory analysis 48
8.5.5.2 Viral load 48
8.5.5.3 RAS Fingerprint 48
8.5.5.4 Exploratory endpoints 49
8.5.5.5 Electrocardiogram 49
8.5.5.6 Vital parameters 50
8.5.6 Definition of the end of the trial 50

9 SAFETY DEFINITIONS AND REPORTING REQUIREMENTS 51
9.1 Averse events (AEs) 51
9.1.1 Summary of known and potential risks of the study drug 51
9.1.1.1 Treatment arm 1: Chloroquine and hydroxychloroquine 51
9.1.1.2 Treatment arm 2: Lopinavir/ritonavir 55
9.1.1.3 Treatment arm 4: IVIG 59
9.1.1.4 Substudy-A: Rivaroxaban 59
9.1.1.5 Substudy-B: Candesartan 61
9.1.1.6 Substudy-C: Clazakizumab **Error! Bookmark not defined.**
9.1.2 Definition of adverse events 65
9.2 Serious adverse events (SAEs) 66
9.2.1 Hospitalization – Prolongation of existing hospitalization 67
9.2.2 SAEs related to investigational drug 67
9.2.3 Suspected unexpected serious adverse reactions (SUSARs) 67
9.2.4 Pregnancy 67
9.3 Severity of adverse events 68
9.4 Relationship to study drug 68
9.5 Reporting procedures 69
9.5.1 Reporting procedures for SAEs 69
9.5.2 Reporting procedures for SUSAR 69

9.5.3 Development safety update report 70

10 FOLLOW-UP 71
10.1 Follow-up of study participants including follow-up of adverse events 71
10.2 Treatment after end of study 71

11 STATISTICAL METHODOLOGY AND ANALYSIS 72
11.1 Analysis sets 72
11.2 Sample size considerations 72
11.3 Relevant protocol deviations 73
11.4 Statistical analysis plan 73
11.5 Missing, unused and spurious data 73
11.6 Endpoints analysis 73
11.6.1 Primary endpoint analysis 73
11.6.2 Secondary endpoint analysis 74
11.6.3 Safety and tolerability endpoints 76
11.6.4 Baseline parameters and concomitant medications 76
11.7 Interim analysis 77
11.8 Software program(s) 77

12 DOCUMENTATION AND DATA MANAGEMENT 78
12.1 Documentation of study results 78
12.1.1 Case report form (CRF) 78
12.1.2 Data collection 78
12.2 Safekeeping 78
12.3 Quality control and quality assurance 79
12.3.1 Periodic Monitoring 79
12.3.2 Audit and inspections 79
12.4 Reporting and publication 79
12.4.1 Publication of study results 79

13 ETHICAL AND LEGAL ASPECTS 80
13.1 Informed consent of subjects 80
13.2 Acknowledgement / approval of the study 80
13.2.1 Changes in the conduct of the study 80
13.3 Insurance 81
13.4 Confidentiality 81
13.5 Ethics and good clinical practice (GCP) 81

14 REFERENCES 83

Table 1 VISIT AND ASSESSMENT SCHEDULE

**PERIODS**	**Name**	**SCREENING**		**TREATMENT**					**FOLLOW-UP Treatment arms 1-4**	**Follow-up substudies**
	**Duration**			**28 days**					**Day 29**	**Up to 7 days after recovery**
**VISITS**	**Number**	**1**	**2**	**3**	**3 A**	**3 B**	**3 C**	**4**	**4**	**4**
	**Name**	**Screening**	**Randomization**	**Treatment arms 1-4**	**Substudy A**	**Sub-study B**	**Sub-study C**	**All treatment arms**	**Follow-up(Telephone call/inpatient visit)**	**Follow-up ‘Telephone call/inpatient visit)**
	**Time**			**Day 1-7, or longer**	**Days 1 – discharge**	**Days 1 - recovery**	**Days 1 - recovery**	**Day 1-29**	**Day 29 (±7)**	
**Informed Consent**		X								
**Inclusion / Exclusion Criteria**		X								
**Medical History**		X								
**Concomitant/change in medication**		X	X	X	X	X	X		X	X
**Physical Examination**		X							X, if possible	X, if possible
**Body weight and height**		X							X, if possible	X, if possible
**Vital Signs (BP, PR)**		X		X	X	X (daily, self-reported, telephone or clinical visits 1x/week), or more often if necessary)	X		X	
**12-lead ECG**		X					X		X, if possible	X, if possible

**Laboratory Tests, incl. oropharyngeal swabs**	X		X	X	X	X	3x/week, until discharge	X	X
**Pregnancy Test**	X							X, if possible	X, if possible
**WHO-ordinal scale, NEWS**	X	X					X		
**Study Drug Dispensing / Return**			X	X	X	X		X	X
**Adverse Events**		X	X	X	X	X		X	x

5 BACKGROUND INFORMATION

5.1 Background

In December 2019 a formerly unknown form of pneumonia occurred in Wuhan, China. The infection spread rapidly and is currently prevalent in almost all countries around the globe. Only a few days after its first occurrence the cause of this new disease was found and confirmed by several independent laboratories: the severe acute respiratory syndrome coronavirus 2 (SARS-CoV-2) (2). On March 12th the WHO announced the outbreak of this new virus a "pandemic".

Coronaviruses are highly-diverse, enveloped, single-stranded RNA-viruses, which cause a variety of diseases in animals and humans (3). Noteworthy, two novel viruses caused severe human diseases: SARS-CoV-1 with >800 deaths and a mortality rate of approximately 10% and MERS-CoV, the middle east respiratory syndrome coronavirus, which caused 334 deaths (35% mortality) (2, 4). Although it may be too early to estimate mortality of the current SARS-CoV-2 pandemic, according to the John Hopkins University currently 2 188 194 patients have been tested positively for the disease, of which 147 632 patients died (approximately 4.8%, April 17th, 2020, https://www.arcgis.com/apps/opsdashboard/index.html#/bda7594740fd40299423467b48e9ecf6).

Other calculations consider the time-shift of infection to death (approximately 14days) and report a mortality of approximately 5% in China and 15% outside of China (5). Noteworthy, overflowing health care systems with limited availability of intensive or intermediate care units contribute to the mortality, which may be lower in case of optimal medical therapy. This phenomenon was reported for China, where mortality rates varied between 1% and 12%, depending on the area and the associated breakdown of the healthcare system (6).

The SARS-CoV-1 and supposedly also SARS-CoV-2 facilitates viral enters into target cells via its spike protein and engages pulmonary angiotensin converting enzyme-2 (ACE-2) as entry receptor (7, 8). In short, entry depends on the interaction of the spike protein, ACE-2 and a cellular protease (TMPRSS2) (8). The spectrum of SARS-CoV-2 infection encompasses asymptomatic infection, mild respiratory symptoms and severe pneumonia with acute respiratory distress syndrome (ARDS) and multiorgan failure (7). In their study in almost 200 patients, Zhou et al. reported the following risk factors for non-survivors in multivariable models: age (odds ratio (OR) 1.10; 95% confidence intervals (CI) 1.03-1.17), Sequential Organ Failure Assessment (SOFA) Score (5.65; 95%CI 2.61-12.23) and elevated D-Dimer levels >1.0 µg/mL (18.42; 95%CI 2.62-128.55). In univariate analysis, but also as reported by other authors the following characteristics were risk factors for severe or fatal course of the disease: arterial hypertension, diabetes mellitus, increased liver function parameters, an increased quick SOFA score, leukocyte counts <4 and >10 *109/L, increased serum ferritin levels, increased creatinine levels, increased lactate dehydrogenase (LDH), creatinine kinase (CK), troponin T or I levels, prothrombin time >16s, and increased IL-6 levels as risk factors for a severe or fatal course of the diseases (7, 9-11). In summary, older patients and those with more comorbidities have an increased risk of a severe and possibly fatal course of disease, while some laboratory parameters may be helpful in the early identification of patients at risk of a severe course of the disease.

There is no established therapy for SARS-CoV-2 infection. However, there is some experience from the mentioned SARS-CoV-1 and MERS-CoV epidemic and numerous trials are currently ongoing. Among the most promising candidates with regards to efficacy, but also availability, are chloroquine (or hydroxychloroquine, as available) and lopinavir/ritonavir (12). The impact of the renin angiotensin

system (RAS) on the risk of infection itself, but also on the course of the disease is currently intensively discussed. Currently there are accumulating reports describing patients developing ARDS who show severe signs of hyper-inflammatory reactions (i.e. cytokine storm), which might be responsible for the high demand of mechanical ventilation in this COVID-19 subgroup (13). The potential of IL-6 and IL-6R blockers is being discussed widely and might offer a promising rescue therapy for patients with deterioration of respiratory function or already in need of mechanical ventilation (14). We plan to test Clazakizumab, an IL-6 antibody in this subgroup of COVID-19 patients. The characteristics of the experimental therapies and the current body of literature are summarized, as follows:

"ANTIVIRAL THERAPY"

There are different (yet unproven) approaches to decrease viral replication, and we consider them as equally likely to succeed or fail, and therefore we will randomize to that backbone for the main trial.

Hydroxychloroquine: **THIS TREATMENT ARM IS INACTIVE**

Hydroxychloroquine is a widely used anti-malarial and immunomodulatory substance. Already during the SARS-CoV-1 outbreak hydroxychloroquine showed antiviral effects *in vitro (15)*. Furthermore, first studies in SARS-CoV-2 patients reported positive effects (15, 16). The complete mode of action remains to be elucidated, but the following effects are hypothesized: (i) an altered pH value on the cell surface of cell membranes, which may inhibit fusion of the virus with the cell membrane, (ii) inhibition of nucleic acid replication, glycosylation of viral proteins, viral assembly, new virus particle transport, virus release (17) and (iii) alkalization of the phagolysosome, which hampers the low-pH dependent steps of viral replication including fusing and uncoating (18). Currently, numerous trials investigate the efficacy and safety of hydroxychloroquine in SARS-CoV-2 patients (16). The major advantage of hydroxychloroquine is the vast experience in clinical use, the well-known side-effect profile and the relatively cheap price.

Lopinavir/Ritonavir:

Lopinavir is an approved, human immunodeficiency virus 1 (HIV-1) protease inhibitor that is usually combined with ritonavir, a potent CYP3A4 inhibitor, to increase its half-life. Two case series suggested potential effectiveness of the combination of lopinavir/ritonavir in SARS-CoV-1 (in combination with ribavirin) (19, 20). In a MERS-CoV-1 model in marmosets, the combination was shown to be an effective treatment (21) while in a murine model treatment with lopinavir/ritonavir did not reduce viral loads, while it improved lung function (22). While a recent study indicated no superiority of this treatment over placebo, treatment was initiated late, so that further investigation during the early course of disease appears to be still warranted (1). Furthermore, the amount of protein binding of lopinavir is approx. 99%. Taking the *in vitro* investigated half-maximal effective concentration of 4-10µg/mL for SARS-COV-1 and MERS-COV into account, a higher dose of lopinavir/ritonavir may be required to be effective against SARS-COV-2 (1, 23). In one case of an intoxication with lopinavir (54g)/ritonavir (13.5g) only mild and unspecific side effects occurred (vomiting, diarrhea, etc.) (24). Also in clinical trials investigating different lopinavir/ritonavir doses, no dose-dependency of adverse events was reported (24, 25).

Remdesivir:

Remdesivir is an inhibitor of the viral RNA-dependent RNA polymerase and demonstrated in vitro efficacy against SARS-COV-2 (26). It soon became one of the most promising candidate drugs in the treatment of COVID-19 and was tested in large clinical trials. However, available data are contradictory and it is therapeutic value is currently unknown. Beigel et al. reported that in their randomized trial involving

>1000 patients remdesivir reduced time to recovery in patients with COVID-19 and mortality on day 14 (27). However, the effect on the time to recovery endpoint was mainly driven by the group of patients receiving oxygen supplementation, while the effects were not statistically significant in patients requiring no oxygen supplementation, or in patients who required non-invasive or invasive mechanical ventilation or even extracorporeal membrane oxygenation. Of note, the group of patients requiring oxygen supplementation was also the largest group in the trial and the smaller sample sizes in other groups may have caused the lack of statistical significance. The same was true for day-14 mortality. Interestingly, there was no effect on overall mortality in the entire study period. Moreover, in their study remdesivir reduced the duration of hospitalization and the duration of oxygen requirement. On the other hand, the Solidarity Trial led by the WHO (unpublished interim report, doi: 10.1101/2020.10.15.20209817) found no effects of remdesivir with respect to overall mortality in >5500 patients compared to the control group. In another study including approximately 600 patients with moderate COVID-19, remdesivir showed no statistically significant effect if given for a 10-day course (28). There was a statistically significant improvement in patients treated with a 5-day course of remdesivir compared to standard of care, but the authors concluded that the clinical relevance is highly questionable. Wang et al. investigated the effects of remdesivir in patients with severe COVID-19 and found that it had no effects on clinical outcomes. However, there was a numerical trend to a quicker recovery in patients who received remdesivir within 10 days of symptom onset (29).
In conclusion, the net clinical benefit of remdesivir remains to be established and the patient cohort most likely to benefit from treatment with remdesivir with regards to disease duration and to disease severity remains to be defined.

"ADJUNCTIVE THERAPIES"

Apart from antiviral therapy a number of possible adjunctive therapies (anticoagulant, antihypertensive or anti-inflammatory) may act synergistically and disease modifying, and patients may receive one of these treatment arms in addition to the "antiviral therapy" of the main trial. If patients do not fulfill the inclusion/exclusion criteria for the antiviral therapy, they may still be eligible for the substudies.

Rivaroxaban

D-dimer, a marker of (coagulation and subsequent) fibrinolysis is massively elevated in severe, critical or lethal COVID-19 (7, 30). D-dimer was also the strongest predictor of clinical outcome (OR 18; 95%CI: 3-129) in Zhou' study (7). Patients with coronavirus pneumonia frequently have coagulopathy (31), and 71% of non-surviving patients developed disseminated intravascular coagulation during their hospital stay. While autopsy studies have not yet been published, hyaline membrane formation (ie. fibrin deposits severely compromising gas exchange) are a hallmark of ARDS. Shi et al. (32) have shown that rivaroxaban can ameliorate lung injury by inhibiting PAR-2 mediated signaling in a mouse model. We hypothesize that rivaroxaban, a small molecule direct anticoagulant with a volume of distribution of ~50L will be able to reach the lung space and inhibit pulmonary coagulopathy and subsequent formation of fibrin deposits (ameliorating gas exchange). We further hypothesize that standard care using the usual prophylactic anticoagulation with low molecular weight heparins may be less effective in this regard because of the limited volume of distribution of 5L (i.e. their confinement to the intravascular space), and their dependency on antithrombin which may decrease during later stages of systemic inflammation/sepsis.

Renin-Angiotensin-System (RAS) blockade:

The RAS system is closely linked with SARS-CoV-2 infection. As mentioned above SARS-CoV-2 engages ACE-2 for cellular entry. In a controversial correspondence letter submitted to Lancet Resp Medicine, ACE-2 was reported to be up-regulated in patients with diabetes and hypertension under the assumption that these patients might be treated with RAS-blockers (33), but the cited references (34-36) to the best of our understanding did not provide further evidence for this hypothesis. The authors concluded that pre-existing treatment with RAS blockers might therefore facilitate infection with SARS-CoV-2. Interestingly, the Council on Hypertension of the European Society of Cardiology immediately replied that ACE-i and ARB might be rather protective against serious lung complications in patients with SARS-CoV-2 and should, in any case, not be discontinued (37). Various animal models of ARDS showed beneficial effects of RAS interference through an ACE-i (captopril) or ARB (losartan) [reviewed in (38)], although clinical data, especially from randomized trials are currently lacking. Thus, understanding the role of RAS and its components in SARS-CoV-2 is complex and it remains unclear whether RAS blockade may be detrimental before infection with SARS-CoV-2 or whether it may be beneficial if infected. The current project aims at investigating these complex relationships, and simultaneously employs the RAS-fingerprint, which uses mass-spectrometry-based quantification of angiotensin metabolites, as previously described (39-41).

<u>Asunercept</u>

Asunercept (CD95-Fc) (Apogenix AG, Heidelberg, Germany) is a fully human CD95L-binding protein. It consists of the extracellular domain of human CD95 fused to the Fc region of human IgG1. The drug interferes with CD95-dependent signaling by binding to CD95L, thereby blocking subsequent CD95-dependent activation (42). The natural role of CD95L is to maintain homeostasis by induction of apoptotic cell death in immune and other cells, most importantly in T cells as CD95L has been demonstrated to mediate activation induced cell death (AICD) in T cells (43). Dysregulation of this key immune checkpoint has been described in various diseases, including in solid tumors and hematological malignancies, but also in viral infections, importantly most recently including in SARS-CoV-2. Newly published evidence implicates CD95L in induction of the life-threatening lymphopenia and epithelial damage in COVID-19 patients (44). Lymphopenia, potentially caused by CD95L-mediated AICD, has been shown to be highly associated with dismal prognosis in this disease (45).

Murine models indicate that uncontrolled death of lymphocytes (46) and of lung epithelial cells, followed by pulmonary fibrosis, form a major part of the pathology of virus-induced acute lung failure and virus-induced acute respiratory distress syndrome (ARDS) (47-50). Correspondingly, inhibition of death receptor-mediated pathways severely attenuates virus-induced lung pathology in murine models. Genetic deficiency in the Fas/FasL (CD95/CD95L) system or blockage of apoptotic cells death by zIETD attenuated cell death, inflammation and fibrosis in a reovirus-induced model of acute lung failure (50). Importantly, treatment of mice with CD95-Fc (Fas-Fc), a recombinant protein highly similar to Asunercept, increased survival in an in-vivo model of lethal influenza A virus-infection using 100 microgram/animal (51). Similarly, inhibition of TRAIL with an anti-TRAIL antibody significantly increased survival of influenza A virus-infected mice (48, 52). Thus, inhibition of cell death induced by these two death ligands, TRAIL and CD95L, importantly also when individually inhibited, appears to afford significant therapeutic benefit with regards to virus-induced ARDS. In addition, CD95L inhibitors such as Asunercept (CD95-Fc) may also attenuate virus-induced lymphopenia. In summary, the therapeutic inhibition of CD95L or of TRAIL may provide therapeutic benefit to patients suffering from viral airway infection-induced disease, including when the disease is induced by influenza viruses or SARS corona viruses.

Whereas the clinical development of a TRAIL-blocking biotherapeutic agent has not yet been reported, Asunercept, a first-in-class CD95L (FasL) blocker, has been employed in various clinical trials over the past few years (42, 53). Asunercept can be safely applied to human individuals intravenously (i.v.) up to a dose of 20 mg/kg. A phase II clinical trial in human glioma patients showed that application of Asunercept is safe at a weekly dose of 400 mg in combination with radiotherapy in 58 patients, with no increase in side effects in patients receiving Asunercept as compared to control, yet with a better therapeutic outcome in patients who received Asunercept with radiotherapy as compared to radiotherapy alone (53). There is also additional data on the application the drug in the context of phase II clinical trials of in patients suffering from other diseases, including myelodysplastic syndrome (covered in detail below).

Pentaglobin

1 ml solution of Pentaglobin (Biotest Pharma GmbH) contains 50 mg human plasma proteins, of which at least 95% are immunoglobulin M (IgM, 6 mg), immunoglobulin A (IgA, 6 mg) and immunoglobulin G (IgG, 38 mg). The distribution of the IgG subclasses is approx. 63% IgG1, 26% IgG2, 4% IgG3, 7% IgG4.
In the 2015 CIGMA study with BT086, another IgM-enriched immunoglobulin preparation, in severe pneumonia, it was shown for the first time that patients benefit greatly from substitution with IgM. Lethality was significantly reduced in subgroups of patients with low IgM levels or high C-reactive protein levels.
Since immunoglobulins are natural, human proteins, the use of which in this indication has been approved since 1985, the positive benefit-risk ratio is well established.
The increased dose as compared to the product information is due to the critical condition of the patients (SOFA ≥ 8). The dosages in the technical information are recommendations and can serve as a guidance. In the studies by Rodriguez et al. (2005) and Welte et al. (2015), the higher doses used here have already been applied successfully.

5.2 Study rationale

Currently there is no established therapy for SARS-CoV-19 infections. The current protocol is designed as a randomized platform protocol that aims to compare different experimental therapies with each other and with “standard of care” (SOC) to identify the most effective treatment, but also relevant and possibly dangerous side effects, as rapidly as possible. The overarching aim of this trial is to prevent uncontrolled, single-arm trials that are unable to provide information on efficacy or safety for experimental treatments. The current situation of an unexpected pandemic requires a certain degree of flexibility, which was implemented in the study design. Numerous studies are currently being performed and aim at providing evidence for an effective and safe treatment. Companies are simultaneously interested in delivering new medications and new strategies to treat SARS-CoV-2 infection, prevent ARDS or treat ARDS on an almost daily basis. Likewise, treatments may be proven futile and may lead to an early termination of a treatment arm. Other agents may become available and new treatment arms may be added later in the form of substantial amendments. We chose the current design of a platform trial with various treatment arms to be able to respond quickly to these requirements. Noteworthy, the WHO launched a master protocol, which our project is based on (Coronavirus disease, COVID-2019, R&D. Geneva: World Health Organization, www.who.int/blueprint/priority-diseases/key-action/COVID-19).

All listed substances are currently being used in the treatment of SARS-CoV-2 or have been shown to be effective under certain experimental conditions. The doses chosen for treatment are the approved doses and the ones recommended by respective guidelines, recommendations etc. As no established treatment is available and most of the substances would be used for treatment of these patients anyway, inclusion of patients into this platform trial does not increase the individual risk of each patient with regards to side effects. However, inclusion of patients into our trial may help to identify the most effective treatment as soon as possible and may help the included patients themselves and future SARS-CoV-2 patients. The trial builds on the hypothesis that "antiviral therapy" may act synergistically with adjunctive or supportive optimization of standard of care.

"Antiviral therapy":

Hydroxychloroquine (Inactive treatment arm) is currently used in many patients, although its antiviral effects are not fully understood. Furthermore, patients receive the combination of lopinavir and ritonavir. Although some data hints to efficacy, treatment with these substances has never been properly investigated in randomized controlled clinical trials.

Update amendment: The hydroxychloroquine arm has been set inactive due to published data indicating safety issues (54). Negative results were also published for the combination of lopinavir and ritonavir (1, 55). However, in this trial a higher dose of lopinavir/ritonavir is used, which may be more effective than above mentioned trials. Therefore, continuation of this arm seems justified in spite of published data.

Expectedly, "best standard of care" has also changed within the last couple of months. Remdesivir was superior to placebo in a randomized trial with regards to time to recovery in hospitalized COVID-19 patients, as discussed in detail above (27). The findings of this trial resulted in the approval of remdesivir for COVID-19 treatment. However, other randomized trials including the large SOLIDARITY trial led by the WHO challenged these results and mostly found no or clinically irrelevant effects of remdesivir. Moreover, availability of remdesivir may become limited in certain hospitals, which also depends on the number of COVID-19 patients admitted to these hospitals. Therefore, we believe it is ethically acceptable to use remdesivir in a randomized trial, in hospitals where remdesivir is not part of standard of care and to test it against other antiviral substances or against best standard of care. However, since remdesivir is approved for COVID-19 treatment, the ultimate decision to use remdesivir will be in the hands of treating physicians.

Dexamethasone treatment reduced 28-day mortality in patients receiving oxygen and in patients requiring invasive ventilation (56). Since only such patients will be eligible for this trial, all patients may receive dexamethasone, based on the judgement of the treating physicians or local standard of care. Overall, the observed effect sizes of dexamethasone, but also of remdesivir, as discussed in detail, were relatively small and many patients still required intensive care or even died within these trials. Therefore, more effective treatments are still desperately required, which is why this study will also continue to recruit patients.

Adjunctive Therapies:

Rivaroxaban has shown to ameliorate lung injury in a mouse model (32), and may inhibit pulmonary coagulopathy and subsequent fibrin deposits (hyaline membranes) in the lungs.
RAS blockers have served as standard medications for arterial hypertension and heart failure for decades. The specific properties of SARS-CoV-2, specifically the interaction with ACE-2, have put this drug class in the focus of a broad and controversial scientific discussion. Recent reports on safety concerns of ACE inhibitors and ATII blockers in patients with SARS-CoV-2 infection have caused uncertainty perhaps due to the large number of patients currently on treatment with these drugs (JAMA interview Anthony Fauci head of NIH, March 18th, 2020). Controversially, preclinical evidence, hoverer, suggests a potential beneficial effect of RAS blockers in ARDS (38, 57). There is a lack of evidence with respect to RAS inhibitor therapy in patients with SARS-CoV-2 infections to guide physicians and patients. First and foremost, data are needed to provide evidence for the safety of continuing RAS inhibitor treatment in patients with SARS-CoV-2 infections. Additionally, a broader understanding of the complex interplay of the constituents of RAS could provide important insight in disease evolution, development of a severe disease course and produce evidence whether intentional RAS blockade by ACE inhibitors or ATII blockers *de novo* could even be beneficial in patients with SARS-CoV-2 infection.

Asunercept:
Intriguingly, alveolar epithelial cell death plays a critical role in the pathogenesis of ARDS (58). There are various lines of evidence from distinct angles and disease scenarios of severe lung damage that suggest a critical involvement of CD95L in, if not its responsibility for, the pathological cell death that occurs in the lungs of COVID19 patients, contributing to severe lung damage, development of ARDS and, ultimately the death of patients with COVID-19:

1. Soluble CD95L is found in the bronchoalveolar lavage fluid of patients with ARDS and other types of lung injuries (59-62).

2. Activation of the CD95L/CD95 pathway induces lung injury and fibrosis in vivo (63, 64).

3. CD95L blockade prevents influenza (65), bacterial (66), bleomycin-, LPS or haemorrhagic shock-induced lung injury in vivo (60, 61, 67-69).

4. Cell death induced by immune cell-derived CD95L is responsible for influenza-induced lethality in cIAP2-deficient mice (70). Therapeutic inhibition of CD95L with CD95-Fc significantly reduces influenza-induced lethality in mice (51).

In addition, virus-infection-induced lymphopenia, which is associated with dismal prognosis in ARDS patients in general but also in COVID-19 patients specifically, may be promoted by the CD95/CD95L system whose known primary function is lymphocyte homeostasis (43). Together, this provides the scientific rationale for the therapeutic inhibition of the death ligand CD95L with CD95-Fc (Asunercept) with the intention of providing an effective treatment for COVID19 patients by preventing alveolar epithelial cell death and the ensuing inflammation which causes ARDS on the one hand, and possibly by contributing to prevent the lymphopenia observed in COVID-19 patients on the other, which consequently altogether often result in the death of these patients.
Regarding the safety of this treatment it is important to note that it was shown that the CD95L/CD95 system is neither required for the clearance of pulmonal bacterial nor influenza infections (65, 66, 70),

and therefore we do not anticipate the blockade of CD95L to have a negative effect on the clearance of SARS-COV2 virus. Indeed, blocking Fas/FasL was shown to facilitate bacterial clearance in lungs (71).

Pentaglobin

IgM enriched intravenous immunoglobulin (IgGAM) may play a role in modulating an immune system that is in a hyperinflammatory state. Tailored therapy in stage IIb hinges on the use of immunomodulatory agents to reduce systemic inflammation before it overwhelmingly results in multi-organ dysfunction.

Rodriguez et al. 2005 showed in a prospective, randomized, double-blind, multicentre clinical study with 56 patients the effectiveness of Pentaglobin® (IgGAM) in patients with peritonitis and severe sepsis / septic shock (72).

According to a 2007 meta-analysis, 8 studies performed with IgGAM on 560 patients showed a pooled relative risk of death of 0.64, whereas the pooled effect of 7 studies performed with IVIG on 932 patients was 0.85 (73).

Nevertheless, there is still insufficient data, so that experts - in relation to the above meta-analysis – are only able to pronounce a grade C recommendation in the German sepsis guidelines for IgGAM. No more recent data are available. This study is therefore intended to significantly expand the data on adjunctive therapy with IgGAM in severe pneumonia and ARDS in COVID-19.

6 STUDY OBJECTIVES

The overall objective of this trial is to identify effective and safe treatments for SARS-CoV-2 infection. Various treatments are currently already used, although data on their efficacy for SARS-CoV-2 are lacking. These treatments will be compared with each other in this randomized controlled trial.

6.1 Primary objective

To investigate the efficacy of various experimental therapeutics for patients with laboratory-proven SARS-CoV-2 infection. A 7-category ordinal scale for clinical severity assessment as proposed by the World Health Organization will be used for efficacy assessment:

- Time to sustained (>48h) improvement of one category from admission
- Clinical status, assessed once daily
- Mean change in the ranking on an ordinal scale from baseline (assessed once daily)

6.2 Secondary objectives

- To assess efficacy according to the National Early Warning Score (NEWS Score): time to
    - Time to discharge or to a NEWS score of ≤2 and maintained for 24 hours, whichever occurs first
    - Change from baseline until day 29, discharge or death, assessed once daily
- Oxygenation
    - Oxygenation free days until day 29
    - Incidence and duration of new oxygen use during the trial
- Mechanical Ventilation
    - Ventilator free days until day 29
    - Incidence and duration of new mechanical ventilation use during the trial
- Viral load/viral clearance using PCR-based assays.
- Hospitalization
    - Duration of hospitalization
    - Duration of intensive care unit treatment
    - Intensive care unit admissions
- Mortality
    - 15-day, 29-day, day 60 mortality, day 90 mortality
- Sub-Study A: number of thromboembolic events
- Exploratory assessment of transaminases (including alkaline phosphatase, gamma-glutamyltransferases (GGT), aspartate aminotransferase (ASAT), alanine aminotransferase (ALAT)) and liver function parameters (including bilirubin, prothrombin time, international normalized ratio, albumin, fibrinogen) and their course during the disease and treatment
- An exploratory endpoint will encompass a comprehensive assessment of inflammatory parameters and their changes during treatment and wash out time, as well as exploratory genotype and RNA analysis with a focus on inflammation, coagulation, and the specific pathophysiology of the disease (if possible for center).

- Within all patients, the impact of obesity and associated diseases on mortality will be investigated
- Safety
    - Cumulative incidence of serious adverse events
    - Discontinuation or temporary suspension of therapy
    - Changes in white cell count, hemoglobin, platelets, creatinine, glucose, total bilirubin, alanine aminotransferase (ALT), aspartate aminotransferase (AST), uric acid over time
    - Occurrence of drug induced liver injury (DILI) in various treatment arms
    - Sub-study A: bleeding events
- Treatment Arm 2: Analysis of CYP3A4 interaction of lopinavir/ritonavir with other prescribed medication in a retrospective manner
- Sub-study B: To investigate clinical outcomes (blood pressure, dyspnea, body temperature, patient-reported fear/anxiety) and the RAS-fingerprint of patients with SARS-CoV-2 infection and arterial hypertension randomized to RAS blocking ("stay-on-any-RAS-blockade" for those patients with previously treated hypertension; candesartan for those with blood pressure ≥140/90, measured twice after SARS-CoV-2 infection) versus non-RAS blocking treatment (nitrendipine or doxazosin).
- Sub-study C: modified SOFA score, paO2/FiO2 ratio, or SpO2/FiO2 ratio
- Pharmacokinetics of antiviral substances, if assays are available (e.g. trough level analysis of lopinavir/ritonavir)
- Sub-Study C for Pentaglobin: CRP, PCT, IL-6, differential blood count (baseline and day 2, 3, 7), IgM, IgA and IgG-levels (baseline, day 2, 3, 4, 5), modified SOFA score (baseline, on day 7 and day 28 of study participation)

7 STUDY DESIGN

The current study design, a multi-arm adaptive platform trial, was chosen to adjust to the very dynamic circumstances of the SARS-CoV-2 pandemic. Furthermore, its design, but also the choice of endpoints, was based on the master protocol published by the World Health Organization (74). Numerous trials are currently being conducted and new information, both on trial results and on potential new experimental treatments, are being published on a daily basis. In this context, the choice of an adaptive, multi-arm platform design seems most suitable to be able to rapidly adjust to new circumstances. Some of the treatment arms may only be opened when the respective product and all necessary documentation becomes available (e.g. IVIgG). A new treatment arm will only be opened after submission of a substantial amendment. Moreover, new treatment arms may be added later and other treatment arms may be closed, if proven futile or even harmful (always with notification/submission of substantial amendments to the ethics committee or competent authorities). Since there is no established treatment for COVID-19 inclusion of a SOC arm seems justified and allows another dimension of comparison. While the SOC may differ between hospitals, it may include currently available, approved treatments for COVID-19, e.g. remdesivir and/or dexamethasone, but may also include other treatments which have been used well before remdesivir has become available. If remdesivir is not part of SOC, then patients may be randomized to receive remdesivir vs. SOC. However, since remdesivir is the only approved treatment of COVID-19, treating physicians may still decide to use remdesivir, if the clinical situation requires it. Sub-study A will only be available to patients without therapeutic anticoagulation. Sub-study B will only be opened for patients with a blood pressure >130/85 mmHg. In these patients, treatment with the lowest possible dose of candesartan is considered safe. The required dose will be titrated to normotension and may therefore be increased. However, in- and outpatients may be recruited. Two control groups will be included: Control group 1: In- and outpatients with suspicion of COVID-19, but with negative test results and control group 2: healthy volunteers with negative oral/nasopharyngeal swabs for SARS-COV-2. Sub-study C, designed to investigate immune-modulatory treatments/anti-inflammatory treatments vs. standard care (±placebo) will only be opened for patients with pneumonia, oxygen demand and incipient or established acute respiratory distress syndrome (in detail in in- and exclusion criteria). Patients may be included in the “main trial” or in sub-studies only. Patients may also only participate in one or more sub-studies or in the “main” antiviral part of the trial. Thus, one patient may also be eligible to more than one treatment arm.

There is no established, effective treatment for SARS-CoV-2 (or the disease SARS-CoViD-19). The aim of this trial is to answer several questions as rapidly as possible and to provide potentially beneficial treatment to these patients, always based on current knowledge and scientific data. Due to multiple arms with drugs of different sizes and formulation it will be impossible to blind the trial except for sub-study C.

Patients may be eligible for sub-studies even if they do not fulfill criteria for antiviral therapy.

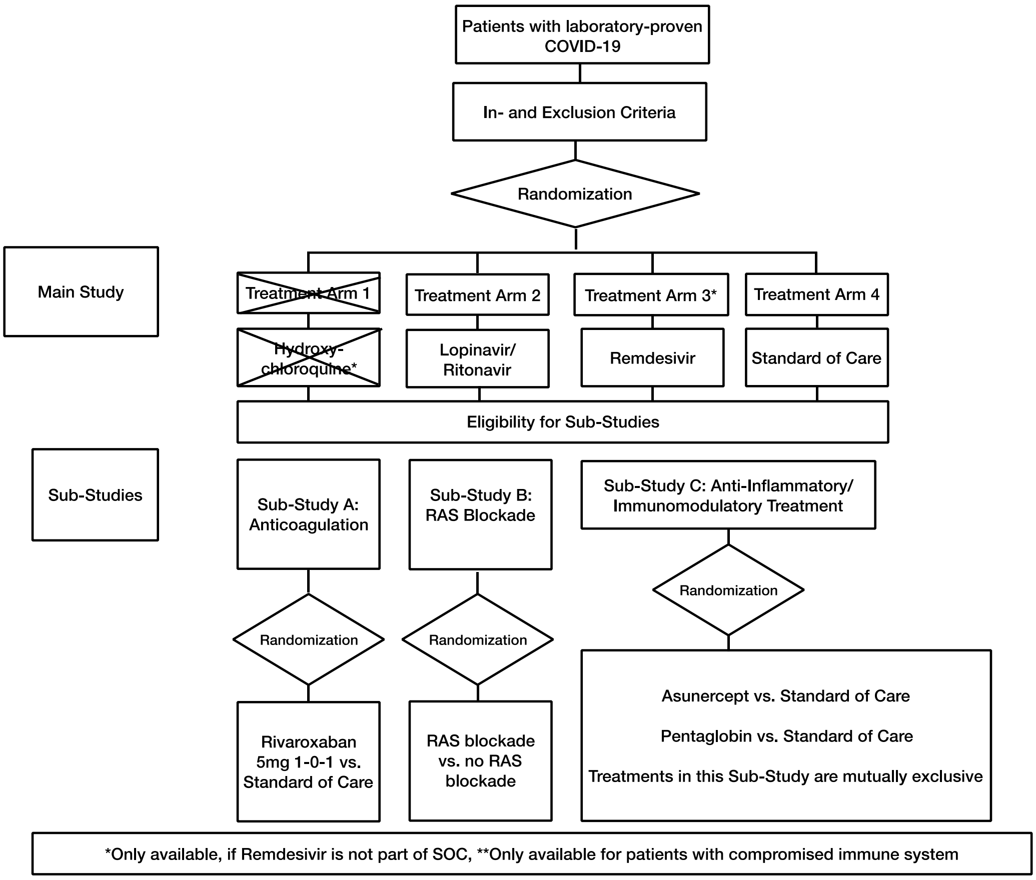

Figure 1. flow-chart of the platform trial

Patients randomized to antiviral treatments are also eligible for the sub-studies. In contrast to other treatment arms and sub-studies, sub-study B may include in- and outpatients. For Sub-study B two further control groups will be included: control group 1: in- and outpatients with a suspicion of COVID-19, but who tested negatively for COVID-19 (n=10) and control group 2: healthy volunteers (n=10) will be included as another control group. Control group 1 will be recruited at the Departments of emergency medicine and the Department infectiology at the Medical University of Vienna. Control group 2 will be recruited at the Department of Nephrology, in collaboration with the Department of clinical pharmacology.
Sub-study C: depending on available medication in the respective trial centers, randomization to one of the substances will be possible.
Treatment arm 1 is inactive due to international reports on safety issues for (hydroxy-)chloroquine.

7.1 Study population

7.1.1 Subject population

The study population of the main study will consist of approximately 500 patients with a laboratory- or radiologically proven (i.e. PCR-based assay) infection with SARS-CoV-2. For those patients who have clear radiological signs of COVID-19, but a negative PCR result, repetitive PCR tests and an antibody-based

assays are required. The sub-studies A and B also aim to include 100 patients, while the initially planned sample sizes for sub-study C are n=300 (3 different doses for 100 patients each) plus 100 control patients for asunercept and n=20 for pentaglobin (plus 20 controls). Patients of the main study may participate in different sub-studies, thus treatment arms are not mutually exclusive. Treatments within one sub-study (or within the main trial) are mutually exclusive. For instance, within sub-study C patients may only be randomized to one treatment. Patients may also only participate in sub-studies, but not in the main trial (e.g. sub-study C). All patients in the study are diagnosed with COVID-19 except for the two control groups of sub-study B. For "antiviral" treatment arms only hospitalized patients will be able to participate, for sub-study A only patients without chronic anticoagulation are eligible, for sub-study B only patients with blood pressure >130/85mmHg in two repetitive measurements will be included. Noteworthy, only sub-study B may also include outpatients. Sub-study B may include two control groups: patients with suspicion of and negative test results for COVID-19 (Control Group 1) and healthy volunteers with negative test for COVID-19 (Control Group 2). Control group 1 will be recruited at the Departments of emergency medicine and the Department infectiology at the Medical University of Vienna. Control group 2 will be recruited at the Department of Nephrology, in collaboration with the Department of clinical pharmacology. For sub-study C, patients showing signs of respiratory deterioration will be included.

7.1.2 Inclusion criteria

- Laboratory confirmed (i.e. PCR-based assay) infection with SARS-CoV-2 (ideally but not necessarily ≤72 hours before randomization for "antiviral" treatments) OR radiological signs of COVID-19 in chest X-ray or computed tomography
- Hospitalisation due to SARS-CoV-2 infection, except for sub-study B, which may also include outpatients with COVID-19
- Requirement of oxygen support (due to oxygen saturation <94% on ambient air or >3% drop in case of chronic obstructive lung disease)
- Informed Consent obtained, the patient understands and agrees to comply with the planned study procedures, except for sub-study C: obtaining informed consent may be impossible due to the severe condition of the patient and may be waived
- ≥18 years of age
- Sub-study A: not on chronic anticoagulation
- Sub-study B: Sub-study B: blood pressure ≥130/85mmHg in 2 consecutive measurements OR patients with established and treated hypertension
- Sub-study B: Control group 1: Patients with suspicion of but negative tests for COVID-19. This group may consist of hospitalized and non-hospitalized patients.
- Sub-study B: healthy volunteers
- Sub-study C: Signs of respiratory deterioration and progressing inflammation: need for oxygen supplementation, non-invasive ventilation, high-flow oxygen devices or mechanical ventilation AND CRP levels >5mg/dL (for Pentaglobin only) and ICU admission (for Pentaglobin only)
- For female patients with childbearing potential: willingness to perform effective measures of contraception during the study.

7.1.3 Exclusion criteria

- Moribund, or estimated life expectancy <1 month (e.g. terminal cancer, etc.)
- Patient does not qualify for intensive care, based on local triage criteria
- Pregnancy or breastfeeding
- Severe liver dysfunction (e.g. ALT/AST > 5 times upper limit of normal)
- Stage 4 chronic kidney disease or requiring dialysis for direct anticoagulant treatment
- Allergy or intolerances to experimental substance (ineligibility for treatment arm), for Asunercept known hereditary fructose intolerance
- Anticipated discharge from hospital within 48 hours (for any given reason)
- Contraindications for treatment arm 2 (lopinavir/ritonavir): severe hepatic impairment, CYP3A4/5 metabolized drugs, as deemed relevant by treating physicians
- Contraindications for treatment arm 3 (remdesivir): <40kg bodyweight
- Known active HIV or viral hepatitis
- Substudy A contraindications for rivaroxaban: active bleeding or bleeding diathesis, lesion or condition considered as major risk factor for bleeding, recent brain or spinal injury, recent brain or spinal or ophthalmic surgery, recent intracranial hemorrhage, known or suspected esophageal varices, arteriovenous malformations, vascular aneurysms, major intraspinal or intracerebral vascular abnormalities, ongoing therapeutic anticoagulation, which will be continued, according to clinical practice
- Sub-study B contraindications for nitrendipine: chronic heart failure, allergies, hypersensitivities and intolerances, severe hepatic impairment and/or cholestasis, concomitant therapy with aliskiren-containing medications (for patients with diabetes mellitus or a GFR<60ml/min/1.73m^2), known significant bilateral renal artery stenosis or renal artery stenosis of a solitary kidney
- Sub-study C contraindications for IL-6 blockade: Contraindications: allergies and intolerances, active untreated diverticulitis, inflammatory bowel disease, any treatment with an IL-6 or IL-6R blocking drug (e.g. tocilizumab, sarilumab, siltuximab) <30 days before study inclusion.
- Sub-study C: Known active tuberculosis.
- Asunercept: females of childbearing potential
- Sub-study C with Pentaglobin: Contraindications to Pentaglobin

7.1.4 Females of childbearing potential

Females of childbearing potential may participate in this clinical trial, although a negative pregnancy test is required during screening. Moreover, for the duration of the trial, negative pregnancy tests are required at least once a month. However, after evaluation of day 29 status (hospitalization, clinical performance etc.) the study ends for each participating patient. Thus, a pregnancy test during the screening visit should be sufficient.
Females of childbearing potential may not participate in the asunercept part of the study.

7.1.5 Study duration

The clinical status of all patients will be evaluated on day 29. Thereafter, the trial ends for the individual patient randomized to antiviral treatment arms. The study ends after full recovery (or death) for patients in sub-studies B and C.

7.1.6 Withdrawal and replacement of subjects

Criteria for withdrawal

Subjects may prematurely discontinue from the study at any time. Premature discontinuation from the study means that the subject did not undergo an end of study examination as planned per protocol.

Subjects must be withdrawn under the following circumstances:

- at their own request
- if the Investigator feels it would not be in the best interest of the subject to continue
- if the subject violates conditions laid out in the consent form / information sheet or disregards instructions by the study personal

In all cases, the reason why subjects are withdrawn must be recorded in detail in the CRF and in the subject's medical records. Should the study be discontinued prematurely, all study materials (completed, partially completed and empty CRFs) will be retained.

Follow-up of patients withdrawn from the study

In case of premature discontinuation after study drug intake, the investigations scheduled for the EOS visit will be performed on day 29 after study entry. Day 60-day and 90-day mortality will be assessed, either by telephone visit, mortality register or by chart review (in case of hospitalization). The subjects will be advised that participation in these investigations is voluntary. Furthermore, they may request that from the time point of withdrawal no more data will be recorded and that all biological samples collected in the course of the study will be destroyed.

Replacement policy

Only patients who discontinue the trial before intake of any trial drugs will be replaced. Patients who received at least one dose of the respective treatment will not be replaced, due to the limited availability of trial drugs.

7.1.7 Premature termination of the study

The sponsor has the right to close this study at any time. The IEC and the competent regulatory authority must be informed within 15 days of early termination.

The trial or single dose steps will be terminated prematurely in the following cases by the local PI:

- If adverse events occur which are so serious that the risk-benefit ratio is not acceptable.
- If the number of dropouts is so high that proper completion of the trial cannot realistically be expected.
- If futility of treatment is proven (e.g. relevant publication by other groups)

8 METHODOLOGY

8.1 Study medication

Treatment Arm 1 (INACTIVE):
Active agent and characteristics: Hydroxychloroquine
Trade name of the agent: Quensyl
Manufacturer: Sanofi Aventis
Drug supply: central pharmacy of each trial center
Storage Instructions: room temperature
Route of administration: p.o.

Treatment Arm 2:
Active agent and characteristics: Lopinavir/Ritonavir
Trade name of the agent: Kaletra
Manufacturer: AbbVie
Drug supply: central pharmacy of each trial center
Storage Instructions: room temperature
Route of administration: p.o.

Treatment Arm 3:
Active agent and characteristics: remdesivir
Trade name of the agent: Veklury
Manufacturer: Gilead Sciences
Drug supply: central pharmacy of each trial center
Storage Instructions: refrigerator (2-8 degrees Celsius)
Route of administration: i.v.

Sub-study A:
Active agent and characteristics: rivaroxaban
Trade name of the agent: Xarelto
Manufacturer: Bayer AG
Drug Supply: central pharmacy of each trial center
Storage instructions: room temperature
Route of administration: p.o.

Sub-study B:
Active agents and characteristics: candesartan
Trade names of the agents: Blopress
Manufacturer: Takeda Pharma
Drug supply: central pharmacy of each trial center
Storage Instructions: room temperature
Route of administration: p.o.

Sub-Study C:

Active agent and characteristics: Asunercept
Trade name of the agent: None
Manufacturer: Nuvisan GmbH
Drug supply: central pharmacy of each trial center
Storage Instructions: refrigerated at 2-8 °C until use
Route of administration: i.v.

Active agent and characteristics: Immunoglobulin
Trade name of the agent: Pentaglobin®
Manufacturer: BIOTEST PHARMA GMBH
Drug supply: central pharmacy of each trial center
Storage Instructions: refrigerated at 2-8 °C until use
Route of administration: continuous iv infusion

8.1.1 Dosage and administration

„ANTIVIRAL“ THERAPIES

Treatment Arm 1: Hydroxychloroquine (INACTIVE)
Initial dose: 200mg 2-0-2
Maintenance dose: 200mg 1-0-1
Route of administration: p.o.
Duration: minimum of 7 Days but at least until sustained improvement

Treatment Arm 2: Lopinavir/Ritonavir
Initial dose: 200mg/50mg 4-0-4
Maintenance dose: 200mg/50mg 3-0-3
Route of administration: p.o.
Duration: minimum of 7 Days but at least until sustained improvement

Treatment Arm 3: Remdesivir
Initial dose: 200mg/day
Maintenance dose: 100mg/day
Route of administration: i.v.
Duration: minimum of 5 days in total

„ADJUNCTIVE“ THERAPIES

Sub-Study A: Rivaroxaban
Initial dose: 10 mg 1/2-0-1/2 (10 mg was non-inferior to 40 mg enoxaparin in acutely ill patients (75); the split dose is provided to minimize peak levels and to obtain smaller peak/trough ratios)

Maintenance dose: down-titration allowed in case of high anti-FXa activity before the next dose
Route of administration: p.o.
Duration: during hospitalization, unless discontinuation is necessary for clinical reasons

Sub-Study B: RAS blockade

Initial dose: candesartan 8 mg 1-0-0; (compared to standard of care, e.g. nitrendipin 10 mg 1-0-1, amlodipine 5mg 1-0-0 or doxazosin 2 mg 1-0-1)
Maintenance dose: Uptitration to normotension
Route of administration: p.o.
Duration: Through recovery, unless discontinuation is necessary for clinical reasons

Sub-Study C:

Asunercept
Initial dose: 25 mg, 100 mg or 400 mg
Maintenance dose: same as first dosage, unless other specified by DSMB or interim analysis.
Route of administration: i.v. (in 200 mL NaCl (0.9%)
Duration: up to 4 weeks, treatment will cease when patient can be discharged from hospital (because of significant amelioration) or when patient has deceased.

25mg infusion solution:
For preparation of the infusion solution, 1.25 mL Asunercept (corresponding to 25 mg) is taken from the vial with a syringe and is diluted into the infusion solution. The preparation of the final Asunercept solution for infusion should be performed at room temperature (if possible under aseptic conditions, e.g., laminar flow hood)

100mg infusion solution:
For preparation of the infusion solution, 5mL Asunercept (corresponding to 100 mg) is taken from the vial with a syringe and is diluted into the infusion solution. The preparation of the final Asunercept solution for infusion should be performed at room temperature (if possible under aseptic conditions, e.g., laminar flow hood).

400mg infusion solution:
For preparation of the infusion solution, the complete content of two Asunercept vials (corresponding to 400 mg) is taken with a syringe and is diluted into the infusion solution. The preparation of the final Asunercept solution for infusion should be performed at room temperature (if possible under aseptic conditions, e.g., laminar flow hood).

The total amount of the infusion solution will be approx. 201 – 270 mL depending on the size of the NaCl solution container and the dose of Asunercept.
The IMP solution ready for infusion should be kept at room temperature, and must be applied to the patient within 3 h after preparation (including infusion time).

Asunercept will be administered once per week as an approx. 30 min i.v. infusion in a total volume of approx. 201 – 270 mL and using a commercial 0.2 µm in-line-filter (0.22 µm nominal pore size).

If the weight of a patient is below 50 kg and 400 mg per dose are applied, the infusion time needs to be increased and Asunercept must be administered as an approx. 45 min i.v. infusion.
For safety reasons, patients will be closely monitored throughout infusion. In case of an allergy or incompatibility reaction during infusion, infusion should be stopped and the event must be documented in the CRF.

For Pentaglobin:
dose: continuous IV application of a total dose of 7ml/kg/day
Route of administration: continuous iv infusion over 12h
Duration: 5 days

8.1.2 Study-drug up- and down titration

Sub-study A: Down titration is allowed in case of excessive anti-FXa activity
Sub-study B: Up/down titration of antihypertensive medication until normotension is planned.
Sub-study C: Asunercept: in case of efficacy or safety signals during the interim analyses, treatment arms may be closed and patients still treated with Asunercept may be switched to the safe or effective higher or lower dose.
Dose modification for individual patients
Dose of Asunercept in individual patients may be considered in the event of:

1. In case signs of intolerability or toxicity signs are detected by the clinician. In such case, Asunercept dose may be reduced to the next lower dose group.
2. In case differences in efficacy are seen at interim analyses, patients will be switched from the less effective dose(s) to the more effective dose(s)or to equally effective lower dose(s).

8.1.3 Study drug interruption or discontinuation

The Investigator must temporarily interrupt or permanently discontinue the study drug if continued administration of the study drug is believed to be contrary to the best interests of the patient.
The interruption or premature discontinuation of study drug might be triggered by an AE, a diagnostic or therapeutic procedure, an abnormal assessment (e.g., laboratory abnormalities), or for administrative reasons, in particular withdrawal of the patient’s consent.
The reason for study drug interruption or premature permanent discontinuation must be documented in the CRF.

8.1.4 Study drug premature permanent discontinuation

Study drug premature permanent discontinuation due to an adverse event
If the reason for premature permanent discontinuation of study treatment is an AE, the patient should have a “Premature End of Study (EOS)” visit with all the assessments performed before the study drug discontinuation, whenever possible.

Study drug premature permanent discontinuation due to another reason than adverse event
If the reason for premature permanent discontinuation of study treatment is not an AE, the patient should be withdrawn from the study (withdrawal of consent) and have the end of study (EOS) visit with all the assessments performed before the study drug discontinuation, whenever possible.

8.1.5 Study-drug delivery & drug storage conditions

Hydroxychloroquine, lopinavir and ritonavir, remdesivir and RAS blocking agents are all approved substances that are stored and released by the central pharmacy of each trial center.

Asunercept will be provided for 300 patients (100 for each dose group) by Apogenix AG.

Pentaglobin is an approved substance that is stored and released by the central pharmacy of each trial center.

8.1.6 Study drug packaging and labeling

This is an open label trial. Medication will therefore not be labelled.

8.1.7 IMP administration & handling

All trial substances should be handled and administered according to manufacturer's recommendations. Tablets/pills may be broken or crushed and administered via nasogastric tubes.

8.1.8 Drug accountability

All medication for the open label trials will be dispensed by the local central hospital pharmacies.
Drug Accountability will be recorded at on-going basis on paper form/source data. Drug dispensing to patients (e.g. early discharge) have to be entered into the CRF. Furthermore, the correct intake of IMP or any variations concerning that will be recorded in the (e)CRF at each visit during treatment period.

8.1.9 Procedures to assess subjects compliance

For "antiviral" treatment arms and sub-studies only hospitalized patients will be included and intake of peroral medication will be controlled. In sub-study C all substances will be administered intravenously, therefore therapy adherence will not be an issue. In substudy B, in case of a discharge, patients will receive the required medication for the full treatment course.

8.1.10 Concomitant medication

The treatment for SARS-CoV-2 infection will not interfere with other treatment of patients and the treating physicians may decide upon concomitant medication without limitations. Noteworthy, the interaction potential of some substances, especially ritonavir, is remarkable and this should be kept in mind when treating these patients.

Allowed: in general, concomitant treatment is allowed and will be decided upon by treating physicians, best standard of care, according to local standards

Not allowed: Contraindication of the specific experimental treatments (according to their summary of product characteristics) have to be **considered by treating physicians**

8.2 Randomization and stratification

This is a randomized trial with different active treatments. However, for this platform trial, some special circumstances need to be considered. In the “Main” antiviral study three treatment options remain active within the randomized trial. Sub-study A, B, C, will also be conducted as randomized trials against standard of care. Randomization will be carried out online by www.meduniwien.ac.at/randomizer. No stratification will be applied, except for the asunercept part of the trial. Based on the currently known risk factors, hospitalized patients are probably older and have more comorbidities, compared to a “general population”. Moreover, the sample sizes are relatively small, which precludes further stratification due to statistical reasons.

For sub-study B randomization will occur within the treatment group. In this cohort (labelled as “sub-study B”), patients with established arterial hypertension (and treatment) may be switched and patients with blood pressure ≥130/85mmHg will be randomized to new treatments. Importantly, those patients with blood pressure ≥130/85mmHg but <140/90mmHg, who are randomized to non-RAS blocking treatment will not receive any treatment. Patients will be randomized to the following two treatment options using an allocation ratio of 1:1

- RAS blocker containing treatment (candesartan)
- Non-RAS blocker containing treatment (e.g. nitrendipin/amlodipin/doxazosin)

Thus, clinicians may decide within the first 24 hours if patients who participate in the main antiviral study A will also fulfill the criteria for one of the sub-studies. It is currently not intended to simultaneously enroll a patient in sub-studies A or B. Within sub-studies randomization will be done using the same software.

For sub-study C:

Asunercept: a 3:1 randomization against standard of care will be done, whereas three different doses of asunercept will be tested. Stratification according to the WHO scale at screening will be performed (only WHO scales 4, 5, and 6 are available for inclusion (patients with oxygen demand).

Pentaglobin: randomization against best standard of care will be done.

8.3 Blinding

Not applicable, open label trial.

8.3.1 Emergency procedure for unblinding

Not applicable, open label trial.

8.3.2 Unblinding at the end of the study

For statistical analysis, unblinding will be performed at interim analyses and at the end of the trial.

8.4 Benefit and risk assessment

SARS-CoV-2 is a global pandemic. Current data about its mortality are somewhat diverging and report mortality rates between 1% and 15%, depending on the statistical approach (5). The rapid spread of the disease and the high number of infected patients may cause a breakdown of the healthcare system, which dramatically increased the mortality rate from 1% to 12% in China (6). Similar numbers are expected in Italy. In general, the following risk factors are reported: older age and patients with more comorbidities. However, it is important to note that also young patients without comorbidities may have severe disease courses. To date there is no established, effective treatment. Within the scope of the exponential increase in patient numbers in the current pandemic effective treatments, which possibly reduce the duration of hospitalization or even reduce the number of necessary hospitalizations are of extreme importance to maintain the functionality of the healthcare system within each concerned country. Thus, we believe that the current project may help to answer some important answers in the fight against SARS-CoV-2.
All of the chosen treatments showed promising results *in vivo* or *in vitro,* although data from high quality randomized controlled trials are missing. Hydroxychloroquine, but also lopinavir/ritonavir are already in clinical use, although no data for efficacy from randomized controlled trials are available. These drugs are approved for other diseases and their side effect profile is well-known. For hydroxychloroquine, the risk of clinically relevant arrhythmias needs to be considered. Drug-drug interactions, old age of patients and inflammatory responses may cause relevant changes in the metabolism of both substances, which may cause arrhythmias. QTc monitoring is recommended for these patients, although it may be difficult to perform in isolated patients. It the QTc time is prolonged relevantly during screening, clinicians may exclude patients from this treatment arm. The Department of Clinical Pharmacology may be contacted for an interaction check, if drug-drug interactions are suspected or if clinically indicated. For lopinavir/ritonavir a relatively high-dose was chosen. However, the safety profile is excellent, with mainly gastrointestinal side effects reported in various studies and case reports and available data suggest that higher drug concentrations are necessary.

RAS blockers may alleviate the disease course especially towards ARDS and it is currently unknown whether RAS blockade increases the risk of infection itself. Subjects randomized to RAS blockers treatment are only available for randomization in case of a “high normal blood pressure (≥130/85mmHg) and this treatment should not pose any relevant risks on patients, if started at the lowest available dose. Those with a blood pressure <140/90mmHg randomized to the control group will not receive any treatment. Moreover, hospitalized patients will be monitored for blood pressure anyway. The inclusion of ten healthy volunteers and negatively tested patients as another control groups is scientifically

justified and the associated risks seem acceptable. Only blood samples will be drawn and oral/nasopharyngeal virus swabs will be obtained (to prove SARS-COV-2 negativity).
Asunercept might be able to avoid the need for mechanical intubation in COVID-19 patients and also ameliorate the course of disease in patients in need of high oxygen flow devices, which has been shown to be a risk factor for infectiousness due to the creation of virus particle containing aerosol. Importantly, no particular risks were identified associated with Asunercept in the clinical trials in which it has been employed. Therefore, no risk populations are excluded from participation.

Pentaglobin is human IgM enriched immunoglobulin for intravenous use. It contains immunoglobulin G (IgG) and increased concentrations of immunoglobulin A (IgA) and immunoglobulin M (IgM) with a broad spectrum of antibodies against various pathogens of infectious diseases and their toxins.
Pentaglobin contains the antibody spectrum of the normal population. Because of the increased levels of IgA and especially IgM, Pentaglobin has higher titers of agglutinating antibodies against bacterial antigens than pure IgG preparations. Pentaglobin is made from pooled plasma from at least 1000 donors. With suitable doses of this drug, abnormally low immunoglobulin concentrations can be restored to normal. The mechanism of action has not yet been fully elucidated, but includes immunomodulatory effects.
Pharmacokinetic properties
Human immunoglobulin is immediately and completely bioavailable in the recipient's bloodstream after intravenous administration. It is distributed relatively quickly between the plasma and the extravascular fluid. After about 3-5 days, a balance is reached between the intra- and extravascular compartment.
The half-life of the immunoglobulins contained in Pentaglobin is comparable to the body's own immunoglobulins. This half-life can vary from patient to patient, especially in primary immunodeficiency syndromes.

In conclusion, we believe that the risks for most of the patients, based on side-effect profile of the individual treatments, are acceptable and the benefits, especially in the light of a lack of standard treatment and also with a focus on the upcoming challenges of the healthcare system, are remarkable, for the individual patients, as well as for the large number of expected SARS-CoV-2 patients worldwide.

General Update Amendment: Although, the estimates for COVID-19 associated mortality expectedly decreased (mainly due to a different testing strategy) and although, dexamethasone and remdesivir have partially shown beneficial effects in COVID-19 patients, there is still massive pressure on health systems globally. There is still a requirement for more effective therapies that may help to prevent intensive care unit admission and that may accelerate recovery in hospitalized patients. Currently, in numerous countries the numbers of patients with COVID-19 are rising and shortages in hospital and ICU beds are expectable. Asunercept not only holds the potential to beneficially impact on COVID-19, but may also be effective in other virus-induced acute respiratory distress syndrome. The clinical value of remdesivir is currently illdefined. Therefore, in hospitals and in situations where remdesivir is not part of SOC, patients may be randomized to receive remdesivir, SOC or lopinavir/ritonavir. However, since remdesivir is the only approved drug for COVID-19 patients in other arms may still receive remdesivir, if the clinical situation requires it. This decision will lie in the hand of treating physicians.

8.5 Study procedures

8.5.1 General rules for trial procedures

- All study measures like blood sampling and measurements (vital parameters, ECG, etc.) have to be documented with date (dd:mm:yyyy).
- In case several study procedures are scheduled at the same time point, adverse events will be collected first and study medication will be administered after.
- The dates of all procedures should be according to the protocol. The time margins mentioned in the study flow chart are admissible. If for any reason, a study procedure is not performed within scheduled margins a protocol deviation should be noted, and the procedure should be performed as soon as possible or as adequate.
- If it is necessary for organizational reasons, it is admissible to perform procedures which are scheduled for one visit at two different time points. Allowed time margins should thereby not be exceeded.

8.5.2 Screening investigation

During the screening procedure the patients' eligibility for each trial arm will be checked. The main prerequisite is a laboratory or radiologically proven infection with SARS-CoV-2. After obtaining informed consent the following procedures will be performed:
Vital parameters will be checked (body temperature, blood pressure, heart rate and respiratory rate). An ECG will be written, with special focus on the QTc time (recent ECGs may be used alternatively). For all patients a baseline laboratory analysis is required including differential blood counts, coagulation including D-dimer, electrolytes kidney function parameters (creatinine, blood urea nitrogen), liver function parameters (AST, ALT, total bilirubin), uric acid, blood glucose levels, lactate dehydrogenase levels, creatinine kinase, troponin T, C-reactive protein levels, IL-6, HBsAg, HIV antigen. Alternatively, to avoid blood loss, blood parameters obtained within 24h hours earlier can be used instead. Medical history, allergies and intolerances, and the disease course (first symptoms, etc.) will be documented. A physical examination will be performed. A pregnancy test will be done during the screening visit, and thereafter at least once a month in females with childbearing potential. Noteworthy, most of these procedures are part of normal clinical routine.
If the patient is eligible for study participation, he or she will be randomized in the next step.

Sub-study B: In- and outpatients may be included. For inpatients, blood sampling will be performed as part of their normal routine (3x/week). For outpatients and for patients discharged from hospital early, visits at the home of patients will be organized by the study staff, until the disease has dissolved (>48h no symptoms). For control groups a total of 4 blood samples will be drawn (Baseline and 3x a week, for a week). The visits at home will be specifically planned in order not to break home quarantine of these patients. Study staff will be wearing the required protection during these visits and will not include a risk population.
Healthy volunteers will be included as another control group.

Blood sampling for genetic analyses will be performed during screening, if the separate informed consent form is signed.

8.5.3 Treatment phase (First Day of Treatment = Day 1)

For treatment arms 2 the minimum duration of treatment is seven days but at least until sustained improvement. For treatment arm 3 (remdesivir) treatment duration is 5-10 days. In case of an early discharge from hospital (before 7 days) patients will receive the required treatment to take it at home (treatment arms 2). In case of an early discharge patients will be called (by telephone) on day 11 and 29 to obtain information on the clinical endpoints.
For all patients the clinical endpoints (including WHO ordinal scale, NEW Score, oxygenation, etc.) will be obtained on a daily basis.
Blood pressure, heart rate, respiratory rate, oxygen demand, oxygen saturation, mode of oxygen delivery, concomitant medication is documented as part of routine care. Laboratory analyses (similar to screening investigation, without HBsAg, but with D-Dimer and anti-FXa activity and troponin levels only between day 5 and 11) will be performed three times a week, and in case of clinical necessity possibly more often.
Blood sampling is recommended three times a week, if possible (e.g. Mo, Wed, Fr), to monitor patients for medical reasons. If this is not feasible because it is against hospital rules or puts too much burden on the already stressed health care workers, the frequency of blood sampling can be reduced without being a protocol deviation.
If such assays are available, pharmacokinetic measurements of antiviral substances will be performed (e.g. trough level analysis for lopinavir/ritonavir after 24h and on day 4 to 7).

ADJUNCTIVE THERAPIES
Randomization to one of the adjunctive therapies is desirable but not necessary. To make it more feasible, it is recommended that eligibility for adjunctive therapies is discussed during daily ward rounds.

For sub-study A patients who are not on chronic oral anticoagulation are eligible.
For sub-study B only hypertensive patients (>130/85mmHg) are eligible. Those patients with established arterial hypertension and antihypertensive treatment will be randomly switched to non-RAS-blocking therapy (e.g. nitrendipine or doxazosin, depending on comorbidities and concomitant medication) versus "stay-on-any-RAS-blockade". Patients with previously undiagnosed arterial hypertension will receive candesartan, as RAS blocking agent, and a non-RAS-blocking agent as standard of care (e.g. nitrendipine, amlodipine or doxazosin, depending on comorbidities and concomitant medication). Treating physicians will titrate the dose to normotension and may add other non-RAS blocking therapy if the maximum dose of the chosen antihypertensive medication does not suffice. RAS-fingerprint will be measured at the same timepoints. In case of an early discharge, RAS fingerprint will be measured at least once weekly (i.e. on days 7±1, 14±1, 21±1, 28±1, through recovery).
Additional analysis of local RAS activity in the lung will be performed in bronchoalveolar lavage samples in case of clinically indicated bronchoscopy or lung tissue following autopsy. End-of study visit will be performed after full recovery.
For asunercept the treatment will be administered once per week intravenously up to four weeks, or until hospital discharge or death, whichever occurs first. The clinical endpoints (including WHO ordinal scale,

NEW Score, oxygenation, etc.) will be obtained on a daily basis during visitation or until death or hospital discharge. Blood pressure, heart rate, respiratory rate, oxygen demand, oxygen saturation, mode of oxygen delivery, concomitant medication and radiologic changes regarding CXR/CT/lung ultrasound are documented as part of routine care. Laboratory analyses (similar to screening investigation) including c-reactive protein, D-Dimer, IL-6 and troponin levels will be performed three times a week, and in case of clinical necessity possibly more often.

8.5.4 End-of-study (EOS) examination

For all patients except for sub-study B and, in part, C the study will end on study day 29, although also day 60 and 90 mortality will be assessed. Either a telephone call or, if the patients are still hospitalized, an EOS visit will be performed. Given the specific circumstances, a formal visit at a hospital will not be required for each patient and only be performed for specific circumstances (e.g. ongoing, relevant side effects). Otherwise the telephone call will be the EOS. For sub-study B or C the study will end with patient death or full recovery. In case a telephone call is the EOS, females with childbearing potential will be asked about possible pregnancies, asked to do a pregnancy test or asked about the date of their last menstruation.

For control groups the last blood sample/oral/nasopharyngeal swab will be the EOS.

8.5.5 Laboratory tests

8.5.5.1 Laboratory analysis

Differential blood counts, liver function (total bilirubin, AST, ALT), kidney function (creatinine, blood urea nitrogen), lactate dehydrogenase, creatinine kinase, uric acid, IL-6, albumin, d-dimer levels and troponin T levels will be measured during screening and three times a week thereafter.
HBsAg and HIV antibodies will be measured once during screening.
Pharmacokinetics of antiviral substances, if available (e.g. lopinavir/ritonavir trough concentrations after 24h and once between day 4-7).

8.5.5.2 Viral load

The COVID-19 viral load will be quantified by PCR-based assays at the Department of Virology at the Medical University of Vienna. Blood samples, nasal and oropharyngeal swabs will be drawn during screening and three times a week thereafter until discharge or day 29 (if feasible), whatever occurs first.

8.5.5.3 RAS and bradykinin-system Fingerprint

The single components of the RAS and bradykinin system will be measured by liquid tandem mass spectrometry, as previously published (39). Briefly, equilibrium analysis (76) will be performed from standard heparinized plasma samples. Angiotensin product-to-substrate ratios directly reflect the activity of the corresponding enyzmes, while absolute equilibrium levels will provide information on the state of the soluble RAS. The concentrations of ACE2 will also be measured from serum, using Mca-Ala-Pro-Lys

at room temperature (77). For the RAS fingerprint blood samples will be drawn during routine blood sampling (heparin plasma, 9ml, 3 times a week). Bradykinin fingerprints will be performed in patients enrolled in Sub-study C.

8.5.5.4 Genetic analyses

Genomic DNA will be extracted in patients with SARS-CoV-2 infection from whole blood samples and stored after informed consent to genome-wide genetic analysis. Following genotyping / exome sequencing specific variants will be tested for association with disease course and response to therapy (PAXgene blood DNA tubes, 8.5 ml).
This blood sample should be drawn during screening investigation, but may also be conducted during one of the regular, routine blood sampling procedures.

8.5.5.5 Disease scores and oxygenation indices

The modified sequential organ failure assessment score (mSOFA score) will be analyzed in all patients. The SpO2/FiO2 ratio, as well as paO2/FiO2 ratio (as applicable), will be analyzed.

8.5.5.6 Exploratory endpoints

Exploratory endpoint analysis may include parameter of coagulation, inflammation, pharmacokinetics etc. Therefore, heparin-anticoagulated blood samples will be drawn and will be stored for analysis of exploratory endpoints including coagulation, platelet function, inflammation.

8.5.5.7 Additional laboratory measurements in Sub-Study C

In Sub-Study C additional peripheral blood samples will be collected at time of randomization and at day 7 of treatment: This includes peripheral blood mononuclear cells (PBMCs) for immune repertoire analysis and testing for viral infection of lymphocytes (3x 8 ml EDTA plasma), RNA for gene expression profiling (PAXgene blood RNA tubes, 8.5 ml), SARS-CoV-2 antibody testing including IgG subtypes and specificity analysis (serum 9 ml) as well as bradykinin system fingerprint (heparin plasma, 9ml).
For the Asunercept part of the study, 20mL EDTA-anticoagulated blood, 4 mL citrate-anticoagulated blood and 4.5mL blood to obtain serum will be drawn twice per week for a maximum of 4 weeks. The total blood volume by additional laboratory measurements in the asunercept arm will not exceed 60mL per week.

For the Pentaglobin substudy additional peripheral or central blood samples will be collected:
Immunoglobulin level at baseline, day 2, 3, 4, 5
C-reactive protein, procalcitonin, IL-6, differential blood count: Baseline and day 2, 3, 7

8.5.5.8 Electrocardiogram

Electrocardiograms will be performed on admission, but if feasible also on study day and 7 ± 3 days and before discharge. Specific focus will be on QTc intervals, especially in the lopinavir and ritonavir group.

8.5.5.9 Vital parameters

Vital parameters (blood pressure, pulse and temperature) will be measured at least once daily during visitation and as a routine process during hospitalization. Parameters will be documented in patient charts. However, daily measurements will not be feasible in all cases.

8.5.6 Definition of the end of the trial

The end of the trial is the last visit of the last patient. However, due to the above-mentioned dynamic processes within this pandemic, treatment arms may be closed earlier due to futility.

9 SAFETY DEFINITIONS AND REPORTING REQUIREMENTS

9.1 Averse events (AEs)

9.1.1 Summary of known and potential risks of the study drug

9.1.1.1 Treatment arm 1: hydroxychloroquine (INACTIVE)

Contraindications include hypersensitivities, allergies, retinopathy (maculopathy).

Main side effects include retinopathies, which may develop or worsen. However, this mainly concerns long-term treatment. Patients with existing maculopathies should be excluded and treatment should stop in case of visual disturbances. Furthermore, extrapyramidal disorders, hypoglycemia (especially when taking other antidiabetic substances), cardiac toxicity, bone marrow depression have been reported.

Furthermore, special caution is required when patients with a sensitivity to quinine, with glucose-6-phosphate dehydrogenase deficiency, with porphyria cutanea tarda, and patients with psoriasis are treated.

Side effects of hydroxychloroquine:

System organ class	Undesirable effect	Frequency
Blood and lymphatic system disorders	bone-marrow depression, anaemia, aplastic anaemia, agranulocytosis, leucopenia and thrombocytopenia	*Not known*
Immune system disorders	urticaria, angioedema, bronchospasm	*Not known*
Metabolism and nutrition disorders	Anorexia<br><br>hypoglycemia<br>Hydroxychloroquine may precipitate or exacerbate porphyria.	*Common*<br><br>*Not known*
Psychiatric disorders	affect lability<br>nervousness<br><br>psychosis	*Common*<br>Uncommon<br><br>Not known
Nervous system disorders	Headache	Common

	Dizziness<br><br>convulsions have been reported with this class of drugs.<br><br>extrapyramidal disorders such as dystonia, dyskinesia, tremor	Uncommon<br><br>*Not known*
Eye disorders	blurring of vision due to a disturbance of accommodation which is dose dependent and reversible<br><br>retinopathy with changes in pigmentation and visual field defects can occur, but appears to be uncommon if the recommended daily dose is not exceeded. In its early form it appears reversible on discontinuation of hydroxychloroquine sulfate. If allowed to develop, there may be a risk of progression even after treatment withdrawal.<br><br>Patients with retinal changes may be asymptomatic initially, or may have scotomatous vision with paracentral, peri-central ring types, temporal scotomas and abnormal colour vision.<br><br>Corneal changes including oedema and opacities have been reported. They are either symptomless or may cause disturbances such as haloes, blurring of vision or photophobia. They may be transient and are reversible on stopping treatment.<br><br>cases of maculopathies and macular degeneration have been reported (the onset	*Common*<br><br>*Uncommon*<br><br>Not known

	ranging from 3 months to several years of exposure to hydroxychloroquine) and may be irreversible.	
Ear and labyrinth disorders	Vertigo, tinnitus<br><br>Hearing loss	*Uncommon*<br><br>Not known
Cardiac disorders	cardiomyopathy which may result in cardiac failure and in some cases a fatal outcome<br><br>Chronic toxicity should be considered when conduction disorders (bundle branch block/atrioventricular heart block) as well as biventricular hypertrophy are found. Drug withdrawal may lead to recovery.	*Not known*
Gastrointestinal disorders	abdominal pain, nausea<br><br>diarrhoea, vomiting<br><br>These symptoms usually resolve immediately on reducing the dose or on stopping treatment.	*Very common*<br><br>*Common*
Hepatobiliary disorders	abnormal liver function tests<br><br>fulminant hepatic failure	*Uncommon*<br><br>*Not known*
Skin and subcutaneous tissue disorders	skin rash, pruritus<br><br>pigmentation disorders in skin and mucous membranes, bleaching of hair, alopecia<br><br>These usually resolve readily on stopping treatment.	*Common*<br><br>*Uncommon*

	bullous eruptions including erythema multiforme<br>· Stevens-Johnson syndrome and toxic epidermal necrolysis<br>· drug rash with eosinophilia and systemic symptoms (DRESS syndrome)<br>· photosensitivity<br>· exfoliative dermatitis, acute generalised exanthematous pustulosis (AGEP).<br>AGEP has to be distinguished from psoriasis, although hydroxychloroquine may precipitate attacks of psoriasis. It may be associated with fever and hyperleukocytosis. Outcome is usually favourable after drug withdrawal.	*Not known*
Musculoskeletal and connective tissue disorders	sensory motor disorders<br>· skeletal muscle myopathy or neuromyopathy leading to progressive weakness and atrophy of proximal muscle groups. Myopathy may be reversible after drug discontinuation, but recovery may take many months.<br>· depression of tendon reflexes and abnormal nerve conduction studies.	*Uncommon*<br>*Not known*
Metabolism and nutrition disorders	hypoglycaemia	*Not known*

Very common: ≥1/10

Common: ≥1/100 to <1/10

Uncommon: ≥1/1,000 to <1/100

Rare: ≥1/10,000 to <1/1,000

Very rare: <1/10,000

Not known: cannot be estimated from the available data

Main interactions:
Hydroxychloroquine is known to elevate digoxin plasma levels, which should be closely monitored, in case of concomitant treatment.
The interactions of chloroquine with other medications may also apply. The interactions with antidiabetics and QTc prolonging agents apply and have been mentioned above.

9.1.1.2 Treatment arm 2: Lopinavir/ritonavir

Contraindications include allergies and intolerances and patients with severe hepatic impairment.

Main interactions:
Ritonavir and lopinavir are both potent CYP3A inhibitors and may interact with drugs that are similarly metabolized. These include (amongst others) alfuzosin, bedaquiline, delamanid, ranolazine, amiodaron, dronedarone, fusidic acid, neratinib, venetoclax, colchicine, astemizole, terfenadine, lurasidone, pimozide, quetiapine, dihydroergotamine, ergonovine, ergotamine, methylergonovine, cisapride, elbasvir, grazoprevir, ombitasvir/paritraprevir/ritonavir ± dasabuvir, lovastatin, simvastatin, lomitapide, avanafil, sildenafil, vardenafil, midazolam, triazolam, rifampicin, fluticasone and other steroids, tenofovir, efavirenz, nevirapine, rilpivirine, maraviroc, raltegravir, fosamprenavir, amprenavir, indinavir, fentanyl, phenytoin, carbamazepine, phenobarbital, valproate, lamotrigine, trazodone, antifungal therapy, calcium channel blockers, cyclosporine, tacrolimus, sirolimus, methadone, bosentan, and St. john's worth. Furthermore, concomitant use of riociguat, vorapaxar, salmeterol, rivaroxaban, atorvastatin, tipranavir is not recommended.

In case of concomitant therapy checking with the summary of product characteristics is recommended.

Special caution is required when treating patients with hepatic impairment, because of a possible deterioration of liver function, hemophilia due to case reports of bleeding, pancreatitis

Side effects:
The most common side effects contain diarrhea, nausea, vomiting, hypertriglyceridemia, hypercholesterolemia.

System organ class	Undesirable effect	Frequency
Infections and infestations	Upper respiratory tract infection	Very common

	Lower respiratory tract infection, skin infections including cellulitis, folliculitis and furuncle	Common
Blood and lymphatic system disorders	Anaemia, leucopenia, neutropenia, lymphadenopathy	Common
Immune system disorders	Hypersensitivity including urticaria and angioedema<br><br>Immune reconstitution inflammatory syndrome	Common<br><br>Uncommon
Endocrine disorders	Hypogonadism	Uncommon
Metabolism and nutrition disorders	Blood glucose disorders including diabetes mellitus, hypertriglyceridaemia, hypercholesterolemia, weight decreased, decreased appetite	Common
Psychiatric disorders	Common<br><br>Uncommon	Anxiety<br><br>Abnormal dreams, libido decreased
Nervous system disorders	Headache (including migraine), neuropathy (including peripheral neuropathy), dizziness, insomnia<br><br>Cerebrovascular accident, convulsion, dysgeusia, ageusia, tremor	Common<br><br>Uncommon
Eye disorder	Visual impairment	Uncommon
Ear and labyrinth disorders	Tinnitus, vertigo	Uncommon
Cardiac disorders	Atherosclerosis such as myocardial infarction, atrioventricular block, tricuspid valve incompetence	Uncommon
Vascular disorders	Hypertension	Common

	Deep vein thrombosis	Uncommon
Gastrointestinal disorders	Diarrhoea, nausea<br><br>Pancreatitis, vomiting, gastrooesophageal reflux disease, gastroenteritis and colitis, abdominal pain (upper and lower), abdominal distension, dyspepsia, haemorrhoids, flatulence<br><br>Gastrointestinal haemorrhage including gastrointestinal ulcer, duodenitis, gastritis and rectal haemorrhage, stomatitis and oral ulcers, faecal incontinence, constipation, dry mouth	Very common<br><br>Common<br><br>Uncommon
Hepatobiliary disorders	Hepatitis including AST, ALT and GGT increases<br><br>Jaundice, hepatic steatosis, hepatomegaly, cholangitis, hyperbilirubinemia	Common<br><br>uncommon
Skin and subcutaneous tissue disorders	Rash including maculopapular rash, dermatitis/rash including eczema and seborrheic dermatitis, night sweats, pruritus<br><br>Alopecia, capillaritis, vasculitis<br><br>Stevens-Johnson syndrome, erythema multiforme	Common<br><br>Uncommon<br><br>Rare
Musculoskeletal and connective tissue disorders	Myalgia, musculoskeletal pain including arthralgia and back pain, muscle disorders such as weakness and spasms<br><br>Rhabdomyolysis, osteonecrosis	Common<br><br>Uncommon

Renal and urinary disorders	Creatinine clearance decreased, nephritis, haematuria	Uncommon
Reproductive system and breast disorders	Erectile dysfunction, menstrual disorders - amenorrhoea, menorrhagia	Common
General disorders and administration site conditions	Fatigue including asthenia	Common

Very common: ≥1/10

Common: ≥1/100 to <1/10

Uncommon: ≥1/1,000 to <1/100

Rare: ≥1/10,000 to <1/1,000

Very rare: <1/10,000

Not known: cannot be estimated from the available data

9.1.1.3 Treatment arm 3: Remdesivir

The most common adverse reactions in healthy volunteers were increased transaminases (14%, 10% grade I, 4% grade II). In COVID-19 patients, the most common side effect was nausea (4%). Interestingly, in a randomized, controlled COVID-19 patients, the incidence of ≥3 non serious adverse events of increased transaminases was 4% in the remdesivir group and 6% in the placebo group. In another study in COVID-19 patients receiving remdesivir, elevations of transaminases >1.25-fold the upper limit of normal occurred in approximately 40% of cases. Grade ≥3 (≥5x upper limit of normal) occurred in 7% of patients. These numbers were similar in other trials.

System organ class	Undesirable effect	Frequency
Immune system disorders	Hypersensitivity	rare
Nervous ystem disorders	Headache	Common
Gastrointestinal disorders	Nausea	Common
Hepatobiliary disorders	Transaminases increased	Very common
Skin and subcuntaenous tissue disorders	Rash	Common
Injury, poisoning and procedural complications	Infusion-related reaction	rare

Very common: ≥1/10

Common: ≥1/100 to <1/10

Uncommon: ≥1/1,000 to <1/100

Rare: ≥1/10,000 to <1/1,000

Very rare: <1/10,000

Not known: cannot be estimated from the available data

ADJUNCTIVE THERAPIES

9.1.1.4 Substudy-A: Rivaroxaban

Contraindications include hypersensitivities, intolerances or allergies against the trial drug or its ingredients, active bleeding, lesion or condition considered as major risk factor for bleeding, recent brain or spinal injury, recent brain or spinal or ophthalmic surgery, recent intracranial hemorrhage, known or suspected esophageal varices, arteriovenous malformations, vascular aneurysms, major intraspinal or intracerebral vascular abnormalities.
Concomitant treatment with unfractionated heparin, low molecular weight heparin, heparin derivatives, oral anticoagulants, except if intended to switch treatments, or when unfractionated heparin is given to at doses necessary to maintain an open central venous or arterial catheter. Hepatic disease associated with coagulopathy and clinically relevant bleeding risk including cirrhotic patients with Child Pugh B and C.

Special caution is required due to the increased hemorrhagic risk, in patients with renal impairment due to accumulation, interaction potential with other substances, other hemorrhagic risk factors, before and after invasive procedures and surgical interventions.

Main interactions include CYP3A4 and P-glycoprotein inhibitors and inducers, other anticoagulants, non-steroidal anti-inflammatory drugs, platelet inhibitors, and serotonin (and noradrenaline) reuptake inhibitors.
The main risk associated with intake of rivaroxaban is minor or major bleeding.

Side effects:

System organ class	Undesirable effect	Frequency
Blood and lymphatic system disorders	Anaemia<br><br>Thrombocytosis, thrombocytopenia	Common<br><br>Uncommon
Immune System disorders	Allergic reactions, dermatitis (allergic), angioedema and allergic edema<br><br>Anaphylactic reactions including anaphylactic shock	Uncommon<br><br>Very rare
Nervous system disorders	Dizziness, headache<br><br>Cerebral and intracranial haemorrhage, syncope	Common<br><br>Uncommon

Eye disorders	Eye haemorrhage	Common
Cardiac disorders	Tachycardia	Uncommon
Vascular disorders	Hypotension, haematoma	Common
Respiratory, thoracic and mediastinal disorders	Epistaxis, haemoptysis	Common
Gastrointestinal disorders	Gingival bleeding, gastrointestinal tract haemorrhage, gastrointestinal and abdominal pains, dyspepsia, nausea, constipation, diarrhea, vomiting<br><br>Dry mouth	Common<br><br>Uncommon
Hepatobiliary disorders	Increase in transaminases<br><br>Hepatic impairment, increased bilirubin, increased blood, alkaline phosphatase, increased GGT<br><br>Jaundice, bilirubin conjugated increased, cholestasis, hepatitis	Common<br><br>Uncommon<br><br>Rare
Skin and subcutaneous tissue disorders	Pruritus, rash, ecchymosis, cutaneous and subcutaneous haemorrhage<br><br>Urticaria<br><br>Stevens-Johnson syndrome/Toxic epidermal Necrolysis, DRESS syndrome	Common<br><br>Uncommon<br><br>Very rare
Musculoskeletal and connective tissue disorders	Pain extremity<br><br>Haemarthrosis<br><br>Muscle haemorrhage<br><br>Compartment syndrome secondary to a bleeding	Common<br><br>Uncommon<br><br>Rare<br><br>Not known
Renal and urinary disorders	Urogenital tract haemorrhage, renal impairment,<br><br>Renal failure /acute renail failure secondary to a bleeding	Common<br><br>Not known

	sufficient to cause hypoperfusion	
General disorders and administration site conditions	Fever, peripheral edema, decreased general strength and energy,<br><br>Feeling unwell<br><br>Localized edema	Common<br><br><br>Uncommon<br><br>Localized edema
Investigations	Increased LDH, lipase and amylase	Uncommon
Injury, poisoning and procedural complications	Postprocedural haemorrhage, contusion, wound secretion<br><br>Vascular pseudoaneurysm	Common<br><br><br>rare

Common: ≥1/100 to <1/10

Uncommon: ≥1/1,000 to <1/100

Rare: ≥1/10,000 to <1/1,000

Very rare: <1/10,000

Not known: cannot be estimated from the available data

9.1.1.5 Substudy-B: Candesartan

Contraindications include allergies, hypersensitivities and intolerances, severe hepatic impairment and/or cholestasis, and concomitant therapy with aliskiren-containing medications is contraindicated for patients with diabetes mellitus or a GFR<60ml/min/1.73m^2.

Special caution should be paid in patients with renal impairment due to risk of hyperkalemia, in patients with heart failure in whom periodic assessments are recommended, in patients requiring hemodialysis, in patients with bilateral renal artery stenosis (or unilateral to a solitary kidney), in patients with a hemodynamically relevant aortic or mitral valve stenosis or obstructive hypertrophic cardiomyopathy. In patients with primary hyperaldosteronism RAS blockade will not show any effects on blood pressure.

There is a general risk towards hyperkalemia and concomitant medication needs to be chosen with respect to this risk. Potassium monitoring may be indicated.

Main interactions:
The interaction with other medications that increase potassium levels have been mentioned. Reversible increases in lithium levels have been described. When NSAIDs are coadministered, the antihypertensive effect may be attenuated.

Side effects for treatment of hypertension (the side effects for treatment of patients with heart failure are not included, since these patients are excluded from the trial):
Side effects:

System organ class	Undesirable effect	Frequency
Infections and infestations	Respiratory infection	Common
Blood and lymphatic system disorders	Leukopenia, neutropenia and agranulocytosis	Very rare
Metabolism and nutrition disorders	Hyperkalaemia, hyponatraemia	Very rare
Nervous system disorders	Dizziness/vertigo, headache	Common
Respiratory, thoracic and mediastinal disorders	Cough	Very rare
Gastrointestinal disorders	Nausea	Very rare
Hepato-biliary disorders	Increased liver enzymes, abnormal hepatic function or hepatitis	Very rare
Skin and subcutaneous tissue disorders	Angioedema, rash, urticaria, pruritus	Very rare
Musculoskeletal and connective tissue disorders	Back pain, arthralgia, myalgia	Very rare
Renal and urinary disorders	Renal impairment, including renal failure in susceptible patients	Very rare

Very common: ≥1/10

Common: ≥1/100 to <1/10

Uncommon: ≥1/1,000 to <1/100

Rare: ≥1/10,000 to <1/1,000

Very rare: <1/10,000

Not known: cannot be estimated from the available data

9.1.1.6 Asunercept

As a generic contraindication of Asunercept (APG101) due to its nature, hypersensitivities against other drugs with Fc components.

Safety information from preclinical studies:

Pivotal toxicology studies were performed in mice (single dose [high dose: 1000 mg/kg], 4 weeks and 28 weeks) and Cynomolgus monkeys (4 weeks and 26 weeks), with doses up to 100 mg APG101/kg bw twice weekly in the repeated-dose studies. In addition, doses up to 300 mg/kg were injected / infused into mice and Cynomolgus monkeys in the GLP safety pharmacology studies. APG101 was very well tolerated, with no findings on body weight, local or systemic tolerance, hematology, ophthalmoscopy, auditory examinations, macroscopic examination, and bone marrow evaluation. In mice, no effects on organ weights and no histopathological findings were noted. A range of pharmacodynamic effects were noted upon exposure of Cynomolgus monkeys to APG101. Their extent was not severe enough to define an adverse effect and they were mostly completely reversible. Apart from two findings with questionable relevance (mild and transient increase of liver enzymes in high dose group monkeys and slight reduction of mean myeloid to erythroid ratio of high dose group male monkeys), all other findings are described in the literature for mutations in the CD95 / CD95L pathway, and are therefore regarded as expected pharmacodynamic effects of APG101.

So far, the pharmacokinetics, safety and efficacy of APG101 were evaluated in the following clinical trials:

1. A phase I study on 34 HV (20 were treated with APG101, 14 received placebo) at a dose range of up to 20 mg/kg body weight
2. A phase II study with patients suffering from first or second relapse of glioblastoma in a dosage of 400 mg/week. 84 patients were included in this study of which 58 patients were randomized in the APG101 arm.
3. A phase I study in low and intermediate risk MDS patients. 12 weekly doses of 400 mg (for the first 6 patients) or 100 mg (for the following patients) were applied to the 20 patients included.
4. A further 19 patients received APG101 under named-patient use conditions.

Based on the data received from the first-in-human study as well as from treatment for named-patient use in patients suffering from glioblastoma or MDS, APG101 was tolerated very well.

Ad 1: Phase I study in HV: In the first in human study in HV, only one AE (headache; intensity: mild) occurred in a healthy volunteer treated with APG101 that was considered related to APG101 in the dose group of 1 mg/kg bw; the subject recovered without sequelae the next day. No change in lab parameters was considered clinically significant and judged as an AE. All AEs and changes in lab parameters were reviewed by a DSMB on an ongoing basis during the study. It was concluded by all members of the DSMB that APG101 was tolerated very well in all doses that were applied to healthy volunteers.

Ad 2: Based on the results obtained with HV, a phase II study in patients suffering from first or second relapse / progression of glioblastoma was started. The safety was determined in terms of adverse events, routine laboratory examination, vital signs, electrocardiograms, neurologic examinations and abdominal sonography. Overall, 83 patients (98.8 %) experienced at least one treatment- emergent AE at the time of database lock. Similar percentages were observed in both treatment groups. Most of these AEs were related to nervous system disorders, general disorders and administration site conditions and gastrointestinal disorders. In 17 patients in the APG101 + RT group, treatment-emergent AEs were judged to be study drug related (including three patients with unknown relationship). No event was judged as definitely related to the study medication.

	Intensity	APG101 + RT (n = 58)		RT (n = 26)		Total (n = 84)	
**Any adverse event**		58	100 %	25	96.2 %	83	98.8 %
	Mild	8	13.8 %	3	11.5 %	11	13.1 %
	Moderate	28	48.3 %	9	34.6 %	37	44.0 %
	Severe	22	37.9 %	13	50.0 %	35	41.7 %

Ad 3: The third clinical study with APG101 was done in myelodysplastic syndrome. A dose reduction from 400 mg weekly over 12 weeks (applied to 5 patient) to 100 mg weekly (given to all other following patients) took place in this study which was not due to safety issues, but to new preclinical data suggesting a higher efficacy with a lower dose. None of the reported AEs raised concerns about safety. Most events are also linked to the clinical symptoms associated with MDS (78). With overall stable vital signs, only few findings in abdominal ultrasound and ECG mostly assessed as not clinically significant and an ECOG of < 2 in nearly all patients, no safety concerns arose during this study. Against this background, APG101 appears to be a safe and well tolerated drug for treating low / intermediate risk MDS patients not impairing the QoL of the patients. Even if 4 SUSARs had to be reported in this trial, APG101 was confirmed to be well tolerable.

9.1.1.7 Pentaglobin

Immunoglobulins are normal components of the human body. The determination of acute toxicity in animals is of no relevance since higher doses lead to circulatory overload. Chronic toxicity and embryo-fetal toxicity studies cannot be performed due to induction and interference with antibodies. Experimental studies on animals are not considered necessary.

Contraindications
· Hypersensitivity to the active substance (human immunoglobulin) or to any of the excipients listed in section 6.1.
· Patients with selective IgA deficiency who have developed antibodies against IgA, since the administration of a preparation containing IgA can lead to anaphylaxis.

Risks:
From a medical point of view, the risk of infection from viruses is so low that there is no additional testing in this context.
Summary of the security profile
Side effects caused by normal human immunoglobulins (ordered by decreasing frequency) include (see also section 4.4):
· Chills, headache, dizziness, fever, vomiting, allergic reactions, nausea, joint pain, low blood pressure and moderate lower back pain
· reversible hemolytic reactions; especially in patients with blood groups A, B and AB and (in rare cases) haemolytic anemia requiring transfusion

· (in rare cases) a sudden drop in blood pressure and, in individual cases, anaphylactic shock, even if the patients have not shown hypersensitivity with previous use
· (in rare cases) transient skin reactions (including cutaneous lupus erythematosus - frequency not known)
· (in very rare cases) thromboembolic reactions such as heart attack (myocardial infarction), stroke, blood clot in blood vessels in the lungs (pulmonary embolism), blood clot in a vein (deep venous thrombosis)
· Cases of reversible aseptic meningitis
· Cases of an increase in serum creatinine and / or acute kidney failure
· Cases of transfusion-associated acute lung insufficiency (TRALI)

Tab. 1 side effects from clinical studies

System Organ Class (SOC) according to MedDRA	Side effects	Frequency
Immune system disorders<br>Vascular disorders	Allergic reaction	occasionally
	Hypotonia	often
Gastrointestinal symptoms	Nausea and vomiting	occasionally
Skin and subcutaneous tissue disorders	Hyperhidrosis	often
	Cutaneous reactions / allergic dermatitis	occasionally
Skeletal muscles, connective tissue and Bone diseases	Back pain	occasionally

Tab. 2 Side effects from post marketing observations

System organ classes (SOC) according to MedDRA	Side effects
Infections and parasitic diseases	Aseptic meningitis
Blood and lymphatic system disorders	Haemolytic anaemia
Immune system disorders	Anaphylactic shock, anaphylactoid reactions, hypersensitivity
Nervous system disorders	Headache, dizziness
Heart disease	Tachycardia
Vascular diseases	Flush
Respiratory disorders, Chest and mediastinum	Dyspnea
Diseases of the skin and the Subcutaneous tissue	Pruritus
Kidney and urinary disorders	Acute kidney failure and / or increase in serum creatinine levels
General disorders and administration site complaints	Fever, chills

9.1.2 Definition of adverse events

An AE is any untoward adverse change from the subject's baseline condition, i.e., any unfavorable and unintended sign including an abnormal laboratory finding, symptom or disease which is considered to be clinically relevant by the physician that occurs during the course of the study, whether or not considered related to the study drug.
Adverse events include:

- Exacerbation of a pre-existing disease.
- Increase in frequency or intensity of a pre-existing episodic disease or medical condition.
- Disease or medical condition detected or diagnosed after study drug administration even though it may have been present prior to the start of the study.
- Continuous persistent disease or symptoms present at baseline that worsen following the start of the study.
- Lack of efficacy in the acute treatment of a life-threatening disease.
- Events considered by the Investigator to be related to study-mandated procedures.
- Abnormal assessments, e.g., ECG and physical examination findings, must be reported as AEs if they represent a clinically significant finding that was not present at baseline or worsened during the course of the study.
- Laboratory test abnormalities must be reported as AEs if they represent a clinically significant finding, symptomatic or not, which was not present at baseline or worsened during the course of the study or led to dose reduction, interruption or permanent discontinuation of study drug.

Adverse events do not include:

- Pre-planned interventions or occurrence of endpoints specified in the study protocol are not considered AE´s, if not defined otherwise (eg.as a result of overdose)
- Medical or surgical procedure, e.g., surgery, endoscopy, tooth extraction, transfusion. However, the event leading to the procedure is an AE. If this event is serious, the procedure must be described in the SAE narrative.
- Pre-existing disease or medical condition that does not worsen.
- Situations in which an adverse change did not occur, e.g., hospitalizations for cosmetic elective surgery or for social and/or convenience reasons.
- Overdose of either study drug or concomitant medication without any signs or symptoms. However, overdose must be mentioned in the Study Drug Log.

9.2 Serious adverse events (SAEs)

A Serious Adverse Event (SAE) is defined by the International Conference on Harmonization (ICH) guidelines and GCP guidelines as any AE fulfilling at least one of the following criteria:

- Results in deaths.
- Life-threatening – defined as an event in which the subject was, in the judgment of the Investigator, at risk of death at the time of the event;
- Requiring subject's hospitalization or prolongation of existing hospitalization
- Resulting in persistent or significant disability or incapacity (i.e., a substantial disruption of a person’s ability to conduct normal life functions).
- Congenital anomaly or birth defect.

- Optional: Is medically significant or requires intervention to prevent at least one of the outcomes listed above

Life-threatening refers to an event in which the subject was at risk of death at the time of the event. It does not refer to an event that hypothetically might have caused death if it were more severe.
Important medical events that may not immediately result in death, be life-threatening, or require hospitalization may be considered as SAEs when, based upon appropriate medical judgment, they may jeopardize the subject and may require medical or surgical intervention to prevent one of the outcomes listed in the definitions above. This means an individual case decision.

9.2.1 Hospitalization – Prolongation of existing hospitalization

Hospitalization is defined as an overnight stay in a hospital unit and/or emergency room.
An additional overnight stay defines a prolongation of existing hospitalization.
The following is not considered an SAE and should be reported as an AE only:

- Treatment on an emergency or outsubject basis for an event not fulfilling the definition of seriousness given above and not resulting in hospitalization.

The following reasons for hospitalizations are not considered AEs, and therefore not SAEs:

- Hospitalizations for cosmetic elective surgery, social and/or convenience reasons.
- Elective treatment of a pre-existing disease or medical condition that did not worsen, e.g., hospitalization for chemotherapy for cancer, elective hip replacement for arthritis.

9.2.2 SAEs related to investigational drug

Such SAEs are defined as SAEs that appear to have a reasonable possibility of causal relationship.

9.2.3 Suspected unexpected serious adverse reactions (SUSARs)

SUSARs are all serious adverse reactions with **suspected** causal relationship to the study drug that is **unexpected** (not previously described in the Summary of Product Characteristics or Investigator's brochure) and serious.

9.2.4 Pregnancy

Any pregnancy that occurs during study participation must be reported to the Investigator/sponsor. To ensure subject safety, each pregnancy must be reported to the Sponsor immediately. The pregnancy must be followed up to determine outcome (including premature termination) and status of mother and child. Pregnancy complications and elective terminations for medical reasons must be reported as an AE or SAE. Spontaneous abortions must be reported as an SAE.
Any SAE occurring in association with a pregnancy brought to the Investigator's attention after the subject has completed the study and considered by the Investigator as possibly related to the investigational product, must be promptly reported to the Investigator/sponsor.

In addition, the Investigator must attempt to collect pregnancy information on any female partners of male study subjects who become pregnant while the subject is enrolled in the study. Pregnancy information must be reported to the Investigator/sponsor as described above.

9.3 Severity of adverse events

The severity of clinical AEs is graded on a four-point scale: mild, moderate, severe, life-threatening and reported on specific AE pages of the CRF.
If the severity of an AE worsens during study drug administration, only the worst intensity should be reported on the AE page. If the AE lessens in intensity, no change in the severity is required.
Mild
Event may be noticeable to subject; does not influence daily activities; the AE resolves spontaneously or may require minimal therapeutic intervention;
Moderate
Event may make subject uncomfortable; performance of daily activities may be influenced; intervention may be needed; the AE produces no sequelae.
Severe
Event may cause noticeable discomfort; usually interferes with daily activities; subject may not be able to continue in the study; the AE produces sequelae, which require prolonged therapeutic intervention.
A mild, moderate, severe AE may or may not be serious. These terms are used to describe the intensity of a specific event (as in mild, moderate, or severe myocardial infarction). However, a severe event may be of relatively minor medical significance (such as severe headache) and is not necessarily serious. For example, nausea lasting several hours may be rated as severe, but may not be clinically serious. Fever of 39°C that is not considered severe may become serious if it prolongs hospital discharge by a day. Seriousness rather than severity serves as a guide for defining regulatory reporting obligations.

9.4 Relationship to study drug

For all AEs, the Investigator will assess the causal relationship between the study drug and the AE using his/her clinical expertise and judgment according to the following algorithm that best fits the circumstances of the AE:
Not related

- May or may not follow a temporal sequence from administration of the study product
- Is biologically implausible and does not follow known response pattern to the suspect study drug (if response pattern is previously known).
- Can be explained by the known characteristics of the subject's clinical state or other modes of therapy administered to the subject.

Unlikely

- There is a reasonable temporal relation between the AE and the intake of the study medication, but there is a plausible other explanation for the occurrence of the AE.

Possibly

- The AE has a reasonable temporal relationship with drug administration.
- The AE may equally be explained by the study subject's clinically state, environmental or toxic factors, or concomitant therapy administered to the study subject.

- The relationship between study drug and AE may also be pharmacologically or clinically plausible.

Probably

- There is a reasonable temporal relation between the AE and the intake of the study medication, and plausible reasons point to a causal relation with the study medication.

Related

- Reasonable temporal relation between the AE and the intake of the study medication and
- There is no other explanation for the AE and
- Subsidence or disappearance of the AE on withdrawal of the study medication and
- Recurrence of the symptoms on restart at previous dose (only applies for re-institution of mediation).

Not assessable

- The causal relationship between the study drug and the AE cannot be judged.

9.5 Reporting procedures

A special section is designated to adverse events in the case report form. The following details must thereby be entered:

- Type of adverse event
- Start (date and time)
- End (date and time)
- Severity (mild, moderate, severe, life-threatening)
- Serious (no / yes)
- Unexpected (no / yes)
- Outcome (resolved, resolving, not resolved, resolved with sequelae, unknown, fatal)
- Relation to study drug (Related/ Probably/ Possibly/ Unlikely/ Not related/ Not assessable)

Adverse events are to be documented in the case report form in accordance with the above-mentioned criteria.

9.5.1 Reporting procedures for SAEs

In case of a serious adverse event, the Investigator has to use all supportive measures for best patient treatment. A written report is also to be prepared and should at least contain the following:

- Patient number
- Patient: sex
- The suspected investigational medical product (IMP)
- The adverse event assessed as serious
- Short description of the event and outcome

If applicable, the initial report should be followed by the Follow up report, indicating the outcome of the SAE.

9.5.2 Reporting procedures for SUSAR

It must be remembered that the regulatory authorities, and the Institutional Review Board / Independent Ethics Committee (IRB / IEC) must be informed about all SUSAR. Such reports shall be made by the sponsor and should content at least the following details:

- Patient number (study code/screening number)
- Patient: age in years, sex
- Name of Investigator and investigating site
- Period of administration
- The suspected investigational medical product (IMP)
- The adverse event assessed as serious and unexpected, and for which there is a **suspected** causal relationship to the IMP
- Concomitant disease and medication
- Short description of the event:
    - Description
    - Onset and if applicable, end
    - Therapeutic intervention
    - Causal relationship
    - Seriousness criteria or reportable reason

Electronic reporting should be the expected method for reporting of SUSARs to the competent authority within 7 calendar days for fatal/life-threatening events and within 15 calendar days for all other serious events. In that case, the format and content as defined by the regulatory requirements should be adhered to. The latest version of MedDRA should be applied. Lower level terms (LLT) should be used.

9.5.3 Development safety update report

A Development Safety Update Report (DSUR) will be provided by the Sponsor annually.
This report will also be presented annually to the Independent Ethics (IEC) and to the competent authorities by the sponsor.

10 FOLLOW-UP

10.1 Follow-up of study participants including follow-up of adverse events

The study will end for the individual patient on day 29. However, in case of ongoing SARS-CoV-2 disease or ongoing side effects regular check-ups (e.g. once a week) will be performed to follow-up on the outcome. Importantly, given the special circumstances these follow-ups will be performed by telephone calls, if possible.

10.2 Treatment after end of study

Within the scope of the study treatment for patients is planned for 1-7 days (28 days for asunercept, 5 days for pentaglobin, 5-10 days for remdesivir). The optimal duration of treatment is currently unknown. Further treatment of patients will be decided by treating physicians on an individual basis. In case of improvement of patient status, a prolonged treatment is not required for patients. In case of a deterioration of the patient status, discharge from hospital may not be possible and patients may receive other treatments.

11 STATISTICAL METHODOLOGY AND ANALYSIS

The statistical analysis in the protocol are outlined for the main part A. More details will be provided in statistical analysis plan (SAP), which will be finalized before the first interim analysis. For sub study A, B and C separate statistical analysis plans (SAPs) will be developed. Importantly, each part of the study (e.g. Asunercept, pentaglobin, etc.) will have its own control group (randomized vs. standard of care) and will therefore be judged as an independent trial within the platform trial. Thus, the first patient in the asunercept trial is the first patient randomized to asunercept vs. standard of care and not the first patient in the platform trial. This is necessary because background therapy is constantly changing in COVID-19 treatment such as by the introduction of remdesivir or dexamethasone.

11.1 Analysis sets

Two different analysis sets are defined
(Modified) Intention to treat set
This analysis set includes subjects who were randomized (and received at least one dose study drug).

Per-protocol set
This analysis set comprises all subjects who received study drug (at least one dose) and did not violate the protocol in a way that might affect the evaluation of the effect of the study drug(s) on the primary objective, i.e., without major protocol violations.

11.2 Sample size considerations

There is currently only limited or no data available on expectable effect sizes. A preliminary sample size calculation was performed based on the primary endpoint defined as time-to-clinical improvement for the comparisons of two groups.
Based on the recent publication of Cao et al. (1) we assumed that the median time in the treatment arm 2 (Lopinavir/Ritonavir) is about 16 days. Assuming an improvement between two groups of 6 days in the median time to clinical improvement, a log-rank test at a two-sided significance level of $\alpha = 0.05$ with a sample size of 100 per treatment group would yield a power larger >80%. These assumptions would translate in a hazard ratio of 1.6 when assuming exponential time-to-event curves.
The sample size calculation was performed with N-Query: When the sample size in each group is 100, with a Total number of events required, E, of 154, an exponential maximum likelihood test of equality of survival curves with a 0,05 two-sided significance level will have 83,15% power to detect the difference between a Group 1 exponential parameter, λ_1, of 0,069 and a Group 2 exponential parameter, λ_2, of 0,043, (a constant hazard ratio of 1,6).
Two interim analyses are planned. The first interim analysis is planned after 20 patients have been included in the open study arms in at least 2 groups. A second interim analysis is planned after 50 patients in a treatment arm and this can trigger a sample size re-calculation.
Possibly, treatment arms may also be closed at the interim analyses.

11.3 Relevant protocol deviations

All protocol deviations will be listed in the study report.

11.4 Statistical analysis plan

Patient demographics and baseline characteristics will be summarized on the ITT set, overall and by treatment cohort, by means of summary descriptive statistics.

For qualitative variables (e.g. sex), absolute (n=) and relative frequencies will be calculated per treatment group. Data will be visualized by bar plots. For quantitative data (e.g. age), the number of valid observations (n =), mean, standard deviation, standard error, median, minimum and maximum will be calculated for each treatment group and each time point separately. Data will be visualized by boxplots and histograms.

11.5 Missing, unused and spurious data

Missing data will not be imputed.

11.6 Endpoints analysis

11.6.1 Primary endpoint analysis

All primary analyses will be on the modified ITT set and the analysis based on the per protocol set will be provided as supportive analysis.

The primary endpoint is time to clinical improvement which is defined as time from randomization to an (sustained) improvement of at least one category on two consecutive days compared to the status at randomization measured on a seven-category ordinal scale (proposed by WHO).

The 7-categories of the World Health Organization proposed scale, as follows:
1. Not hospitalized, no limitations on activities
2. Not hospitalized, limitation on activities;
3. Hospitalized, not requiring supplemental oxygen;
4. Hospitalized, requiring supplemental oxygen;
5. Hospitalized, on non-invasive ventilation or high flow oxygen devices;
6. Hospitalized, on invasive mechanical ventilation or ECMO;
7. Death.

During hospitalization this score will be determined daily (till day 29). If a patient is released from the hospital before day 29, the score will be determined at day 11 and 29 (and day 60 in clazakizumab arm) after randomization (depending when the patient was released or by telephone call).

The time to clinical improvement will be visualized by Kaplan-Meier Plots. The comparisons of the time-to-event curves will be performed using a log-rank at a two-sided level alpha of 5%.
(Please note in the initial phase only treatment arms (1-3, 5) might be available for the platform trial. A closed test procedure will be implemented to control the multiple level alpha. More details will be provided in the statistical analysis plan.
Additionally, a Cox-proportional hazard model will be performed. In this model we will use treatment as factor and it will be adjusted for calendar time to account for time trends (e.g. severity of cases might change over time).
Furthermore, we will include sex and age as factors in the model.

11.6.2 Secondary endpoint analysis

- Clinical Status of patients according to the above-mentioned WHO scale:
    - Time to improvement of one category from admission
    - Clinical status daily
    - Mean change in the ranking on an ordinal scale from baseline
- National Early Warning Score (NEWS):
    - Time to discharge or to a NEWS of ≤2 and maintained for 24 hours, whichever occurs first
    - Change from baseline
- Oxygenation
    - Oxygenation free days until day 29
    - Incidence and duration of new oxygen use during the trial
- Mechanical Ventilation
    - Ventilator free days until day 29
    - Incidence and duration of new mechanical ventilation use during the trial
- Viral load/viral clearance
    - Baseline, and three times a week until infection has resolved
- Hospitalization
    - Duration of hospitalization
    - Duration of intensive care unit treatment
    - Intensive care unit admissions
- Mortality
    - 15-day, 29-, 60-day mortality
- Within all patients, the impact of obesity and associated diseases on mortality will be investigated (e.g. mortality, inflammatory response, duration of hospitalization, intensive care unit admission, new oxygen use, duration of oxygen)
- Treatment arm 2: drug-drug interaction analysis with lopinavir/ritonavir, in a retrospective manner
- pharmacokinetics of antiviral substances, if assays are available (e.g. lopinavir/ritonavir trough level analysis)
- Sub-study A: thromboembolic events

- Sub-study B: Renin Angiotensin System (RAS) fingerprint in patients with SARS-CoV-2 infection randomized to no RAS blocking agents, randomized to angiotensin converting enzyme (ACE) inhibitor treatment and randomized to angiotensin II blockade
  - Quality of life: EuroQoL- 5 Dimension (EQ-5D)
  - Activities of daily living/life participation, Instrumental activities of daily living scale (IADLS)
  - PROMIS social function measures
  - Dizziness
  - Gastrointestinal symptoms including nausea and vomiting (PROMIS gastrointestinal symptom scales)
  - Gastrointestinal symptom rating scale (before respiratory symptoms, many patients with COVID-19 had diarrhea, nausea, vomiting, abdominal discomfort)
  - Headache: headache impact test 6-item (HIT-6)
  - Anxiety: hospital anxiety and depression scale (HADS)
  - Fear/anxiety reported with SARS-CoV-2 infection (qualitative interview at baseline and recovery, GAD-7 scale value at all study visits [telephone or in person])
- Exploratory assessment of transaminases (including alkaline phosphatase, gamma-glutamyltransferases (GGT), aspartate aminotransferase (ASAT), alanine aminotransferase (ALAT)) and liver function parameters (including bilirubine, prothrombin time, international normalized ratio, albumin, fibrinogen) and their course during the disease and treatment
- an exploratory endpoint will encompass a comprehensive assessment of inflammatory parameters and their changes over treatment and wash out time, as well as exploratory genotype and RNA analysis with a focus on inflammation, coagulation, and the specific pathophysiology of the disease.
- Sub-study C: modified SOFA score, paO2/FiO2 ratio, or SpO2/FiO2 ratio

For **binary secondary endpoints** (such as 15 and 29 day mortality, incidence rates) absolute (n=) and frequencies in percent (%) will be calculated per treatment group. 95%-confidence intervals will be calculated for rates, if appropriate. Such data will be visualized with bar charts. If appropriate, logistics regression model will be applied using treatment as independent factor. Furthermore, the model will be adjusted for sex and age.

For **ordinally scaled endpoints** (such as clinical status on day 3, 5, 8, 11 and 29 after admission) absolute (n=) and frequencies in percent (%) will be calculated for all categories separately for each treatment group. Treatment groups will be compared with each other

Continuous secondary endpoints such as “days free of Oxygenation free days within 29” and “Ventilator free days until day 29 days” will be summarized by mean, standard deviation (SD), median, first and third quartiles, minimum and maximum for each treatment arm separately. These secondary endpoints will be analysed using an analysis of covariance (ANCOVA) using the factor treatment adjusting for the factors Sex and the covariate Age (in Years). Mean estimates will be provided, together with their corresponding two-sided 95% confidence intervals. We will conduct further explorative analyses adjusting for calendar time in the models concerned to account for time trends (e.g. severity of cases might change over time).

For **time to event endpoints** (such as time to discharge or to a NEWS of ≤2 and maintained for 24 hours, whichever occurs first) we will provide similar analyses as described for the primary endpoint.

The analyses of all secondary endpoints are considered as exploratory. Unadjusted p-values and 95%-confidence intervals might be presented for secondary endpoints, but these are for descriptive purposes only,

<u>Statistical analysis for sub-studies A-C:</u>
Depending on the type of endpoint, similar statistical analyses as outlined above for the main study will be performed taking only the patients included in the respective sub study. The main comparison depends on the randomization for a specific substudy. If applicable, it will be investigated whether there is an interaction with the treatments options in the main study ("antiviral" treatment arms)

Sub-study A:

- Rivaroxaban as anticoagulant 10 mg ½-0-½
- Best standard of care (which will likely include prophylactic doses of low molecular weight heparin).

Sub-study B:

- RAS blocking agent (Candesartan)
- Best standard of care: non-RAS blocking agents (nitrendipine, amlodipine or doxazosin)

Sub-study C:

- Best standard of care (without IL-6 blockade), or placebo on top of standard of care
- Asunercept at 25mg, 100mg, 400mg once weekly
- Pentaglobin 7mg/kg bodyweight/day continuous iv infusion over 12 hours for 5 days

11.6.3 Safety and tolerability endpoints

- Cumulative incidence of serious adverse events
- Discontinuation or temporary suspension of therapy
- Changes in white cell count, hemoglobin, platelets, creatinine, glucose, total bilirubin, alanine aminotransferase (ALT), aspartate aminotransferase (AST) over time
- Occurrence of drug induced liver injury (DILI) in various treatment arms
- Bleeding events

11.6.4 Baseline parameters and concomitant medications

Baseline parameters, medical history and concomitant medication will be documented during screening and throughout the trial until day 29.
Enrolment, protocol deviations and discontinuations from the study drug and the study will be summarized. Demographics (age, race, ethnicity and sex) and medical history and study drug

administration will also be summarized by treatment group. This will be done for separately for the main study and each sub-study.

For qualitative variables (e.g. sex), absolute (n=) and relative frequencies will be calculated per treatment group. Data will be visualized by bar plots. For quantitative data (e.g. age), the number of valid observations (n =), mean, standard deviation, standard error, median, minimum and maximum will be calculated for each treatment group and each time point separately. Data will be visualized by boxplots and histograms.

11.7 Interim analysis

After inclusion of 20* and 50 patients in a treatment arm an interim analysis will be performed. In case of safety or results indicating futility, treatment arms may also be closed at this step.
The first interim analysis is mainly for safety and futility only (* Note that the first interim analysis will be triggered if at least 2 treatment arms have recruited 20 patients each). In the second interim analysis a sample size reassessment might be performed. To account for the sample size re-assessment, the stagewise p-values using the inverse normal combination function will be used (79-81). To allow for early stopping in case overwhelming effects are observed, an alpha spending function with O´Brien and Fleming (OF) boundaries will be used using a two-sided alpha of 5%. Further details will be provided in the charta of the DMC. Importantly, the interim analyses will not cause a stopping of the trial and will be conducted after patients have been included for two weeks.

11.8 Software program(s)

All statistical analyses will be conducted with statistical software like SAS 9.4. (or higher) and R 3.6.3. (or higher).

12 DOCUMENTATION AND DATA MANAGEMENT

12.1 Documentation of study results

A subject screening and identification Log will be completed for all enrolled subjects with the reasons for exclusion.

12.1.1 Case report form (CRF)

ECRFs will be used for this trial, as provided by the KKS (Koordinationszentrum Klinische Studien).
For each subject enrolled, regardless of study drug initiation, a CRF must be completed and signed by the Investigator or a designated sub-Investigator. This also applies to those subjects who fail to complete the study. If a subject withdraws from the study, the reason must be noted on the CRF. Case report forms are to be completed on an ongoing basis.
If screening failures should not be documented in the CRF, this has to be clearly defined in the protocol.
CRF entries and corrections will only be performed by study site staff, authorized by the Investigator.
In a paper based CRF all forms should be completed and must be legible. Entry errors have to be corrected according the ICH-GCP Guidelines.
The entries will be checked by trained personnel (Monitor) and any errors or inconsistencies will be checked immediately.
The monitor will collect original completed and signed CRFs at the end of the study. A copy of the completed and signed CRFs will remain on site, while the original data are handed out to the sponsor.

12.1.2 Data collection

Data collected at all visits are entered into an interactive form. The CRFs will be source documents verified following guidelines established before study onset as detailed in the Monitoring Plan. Maintenance of the study database will be performed by KKS.

12.2 Safekeeping

The Investigator will maintain adequate and accurate records to enable the conduct of the study to be fully documented and the study data to be subsequently verified (according to ICH-GCP “essential documents”). These documents will be classified into two different categories: Investigator's study site file (ISF) with all essential documents regarding the study conduct, and subject clinical source documents.
The Investigator's file will contain all essential documents listed in ICH-GCP Guidelines section 8.
Subject clinical source documents include all patient hospital clinical records in original version, such as original laboratory reports, ECG, X-ray prints and other reports.
These two categories of documents must be kept on file by the Investigator for as long as needed to comply with the regulatory requirements.

12.3 Quality control and quality assurance

12.3.1 Periodic Monitoring

According to GCP at least 3 monitoring visits are scheduled. An initiation visit, one routine visit and a close out visit after the last patient has finished the study or data base lock .
The designated monitor will contact and visit the Investigator on a regularly basis and will be allowed to have direct access to all source documents needed to verify the entries in the CRFs and other protocol-related documents provided that subject confidentiality is maintained in agreement with local regulations. It will be the monitor's responsibility to inspect the CRFs at regular intervals according to the monitoring plan throughout the study, to verify the adherence to the protocol and the completeness, consistency and accuracy of the data being entered on them.
Monitoring will be performed by the KKS.

3 visits/site are planned, only primary endpoint data will be reviewed.

12.3.2 Audit and inspections

Upon request, the Investigator will make all study-related source data and records available to a qualified quality assurance auditor mandated by the sponsor or to competent authority inspectors. The main purposes of an audit or inspection are to confirm that the rights and welfare of the subjects have been adequately protected, and that all data relevant for assessment of safety and efficacy of the investigational product have appropriately been reported to the sponsor.

12.4 Reporting and publication

12.4.1 Publication of study results

The findings of this study will be published by the sponsor (Investigators) in a scientific journal and presented at scientific meetings. The manuscript will be circulated to all co-Investigators before submission. Confidentiality of subjects in reports/publications will be guaranteed.

13 ETHICAL AND LEGAL ASPECTS

13.1 Informed consent of subjects

Following comprehensive instruction regarding the nature, significance, impact and risks of this clinical trial, the patient must give written consent to participation in the study.
During the instruction the trial participants are to be made aware of the fact that they can withdraw their consent – without giving reasons – at any time without their further medical care being influenced in any way.
In addition to the comprehensive instructions given to the trial participants by the Investigator, the trial participants also receive a written patient information sheet in comprehensible language, explaining the nature and purpose of the study and its progress.
The patients must agree to the possibility of study-related data being passed on to relevant authorities.
The patients must be informed in detail of their obligations in relation to the trial participants insurance in order not to jeopardize insurance cover.

Substudy C: in this part of the trial also patients with severe dyspnea and possibly acute respiratory distress syndrome, who may be mechanically ventilated, are eligible. Hence, obtaining informed consent for participation in the trial may not be possible and informed consent may be waived. All subjects will be informed about their participation as soon as possible.

13.2 Acknowledgement / approval of the study

The Investigator (or a designated CRO) will submit this protocol and any related document provided to the subject (such as subject information used to obtain informed consent) to an Ethics Committee (EC) or Institutional Review Board (IRB). Approval from the committee must be obtained before starting the study.
The clinical trial shall be performed in full compliance with the legal regulations according to the Drug Law (AMG - Arzneimittelgesetz) of the Republic of Austria.
An application must also be submitted to the Austrian Competent Authorities (Bundesamt für Sicherheit im Gesundheitswesen (BASG) represented by the Agency for Health and Food Safety (AGES Medizinmarktaufsicht) and registered to the European Clinical Trial Database (EudraCT) using the required forms. The timelines for (silent) approval set by national law must be followed before starting the study.

13.2.1 Changes in the conduct of the study

Protocol amendments

Proposed amendments must be submitted to the appropriate CA and ECs. Substantial amendments may be implemented only after CA/EC approval has been obtained. Amendments that are intended to eliminate an apparent immediate hazard to subjects may be implemented prior to receiving CA/EC approval. However, in this case, approval must be obtained as soon as possible after implementation.

Study Termination

If the sponsor or the Investigator decides to terminate the study before the planned completion, they will notify each other in writing stating the reasons of early termination. Both the sponsor and the investigator will ensure the protection of the subjects' wellbeing. The sponsor will notify the regulatory authority as well as the ethics committee about the premature termination. Documentation will be filed in the Trial Master File as well as in the Investigator Site File.

Clinical Study Report (CSR)

Within one year after the final completion of the study, a full CSR will be prepared by the sponsor and submitted to the EC and the competent authority.

The Investigator will be asked to review and sign the final study report.

13.3 Insurance

During their participation in the clinical trial the patients will be insured as defined by legal requirements. The Investigator of the clinical trial will receive a copy of the insurance conditions of the 'patients insurance'. The sponsor is providing insurance in order to indemnify (legal and financial coverage) the Investigator/center against claims arising from the study, except for claims that arise from malpractice and/or negligence. The compensation of the subject in the event of study-related injuries will comply with the applicable regulations.

Details on the existing patients insurance are given in the patient information sheet.

All subjects will be insured at the Zürich Versicherungs-AG, Schwarzenbergplatz 15, 1010 Wien, Tel.: +43 1 501255 1255, policy number 07229622-2.

13.4 Confidentiality

The information contained in this document, especially unpublished data, is the property of the principal investigator and the protocol author. It is therefore provided to you in confidence as an Investigator, potential Investigator, or consultant, for review by you, your staff, and an Ethics Committee or Institutional Review Board. It is understood that this information will not be disclosed to others without written authorization from the principal investigator.

13.5 Ethics and good clinical practice (GCP)

The Investigator will ensure that this study is conducted in full conformance with the principles of the "Declaration of Helsinki" (as amended at the 64th WMA General Assembly, Fortaleza, Brazil, 2013) and with the laws and regulations of the country in which the clinical research is conducted.

The Investigator of the clinical trial shall guarantee that only appropriately trained personnel will be involved in the study. All studies must follow the ICH GCP Guidelines and the regulatory requirements.

Therefore this study follows the EU Directive embedded in the Austrian drug act.

Clinical Trial Centers (alphabetically by institution) & Personnel:

Kaiser-Franz-Josef Spital, Department of Infectious Diseases, Division of Medicine 4
PD. Dr. Alexander Zoufaly (Site PI), Prim. PD. Dr Christoph Wenisch, Dr. Erich Pawelka, Dr. Mario Karolyi, Dr. Marianna Traugott, Dr. Tamara Seitz, Dr. Hermann Laferl, c.m. Agnes Abrahamowicz,

Wilhelminenspital, Department of Emergency Medicine and Department of Pulmology
Prim. Priv.-Doz.Dr. Georg-Christian Funk, Dr. Carolina Nell, Dr. Edvin Mrsic
Prim. Assoc.Prof.Priv.Doz.Dr.Alexander Spiel, Dr. Teresa Lindmayr, Dr. Severin Ehrengruber

Krankenhaus Hietzing, Departments of Internal Medicine II &III
Prim Univ. Prof. Dr. Kurt Redlich, Prim Univ. Prof. Dr. Thomas Stulnig, Dr. Andreas Zitterl, Dr. Slobodan Peric, Dr. Boris Lindner

Otto-Wagner Spital, Department of Pulmology
Dr. Brigitte Schmied (Site PI), Dr. Sebastian Schnaubelt

Medical University of Innsbruck, Department of Medicine
Univ. Prof. Dr. Günter Weiss, Ao. Univ. Prof. Dr. Rosa Bellmann-Weiler

Medical University of Vienna, Department of Medicine I, Div. of Infectious Diseases,
Univ. Prof. Dr. Burgmann, Ao. Univ. Prof. Dr Stefan Winkler, Dr. Ludwig Traby, Dr. Zoe Österreicher
Department of Clinical Pharmacology, Medical University of Vienna
Univ. Prof. Dr. Bernd Jilma (Site-PI), Ao. Univ. Prof. Dr. Ulla Derhaschnig, PD. DDr. Christian Schörgenhofer, Dr. Christa Firbas, Sarah Ely, RN, Sabine Schranz, RN, Mag Pharm. Katarina Kovacevic
Associated Departments
Department of Virology, Medical University of Vienna, Austria
Univ.Prof. Dr. Elisabeth Puchhammer, Assoc Prof. Dr. Judith Aberle
Department of Internal Medicine III, Medical University of Vienna, Austria
Assoc Prof. Dr. Manfred Hecking, PhD, Dr. Farsad Eskandary, PhD, Dr. Bianca Itariu
Department of Emergency Medicine, Medical University of Vienna, Austria
Dr. Michael Schwameis, Dr. Juergen Grafeneder (rotation)

Sozialmedizinisches Zentrum Ost
Prim Univ. Prof. Dr. Thomas Stefenelli, Dr.Anna Klicpera, MSC

Medical University of Graz, Department of Medicine
Univ.Prof. Dr. Robert Krause (Site PI), Ao.Univ.-Prof.Dr. Marianne Brodmann, Dr. Philipp Kreuzer

Johannes Kepler University Linz, Department of Medicine
Prim.Priv.Doz.Dr. Bernd Lamprecht (Site PI), Dr. Helmut Salzer

LK Neunkirchen
Ao.Univ.Prof.Dr. Ojan Assadian (Site PI), Prim.Dr. Michael Hüpfl

14 REFERENCES

1. Cao B, Wang Y, Wen D, Liu W, Wang J, Fan G, Ruan L, Song B, Cai Y, Wei M, Li X, Xia J, Chen N, Xiang J, Yu T, Bai T, Xie X, Zhang L, Li C, Yuan Y, Chen H, Li H, Huang H, Tu S, Gong F, Liu Y, Wei Y, Dong C, Zhou F, Gu X, Xu J, Liu Z, Zhang Y, Li H, Shang L, Wang K, Li K, Zhou X, Dong X, Qu Z, Lu S, Hu X, Ruan S, Luo S, Wu J, Peng L, Cheng F, Pan L, Zou J, Jia C, Wang J, Liu X, Wang S, Wu X, Ge Q, He J, Zhan H, Qiu F, Guo L, Huang C, Jaki T, Hayden FG, Horby PW, Zhang D, Wang C. A Trial of Lopinavir-Ritonavir in Adults Hospitalized with Severe Covid-19. N Engl J Med. 2020 Mar 18. Epub 2020/03/19. doi:10.1056/NEJMoa2001282. Cited in: Pubmed; PMID 32187464.

2. He F, Deng Y, Li W. Coronavirus Disease 2019 (COVID-19): What we know? J Med Virol. 2020 Mar 14. Epub 2020/03/15. doi:10.1002/jmv.25766. Cited in: Pubmed; PMID 32170865.

3. Zumla A, Chan JF, Azhar EI, Hui DS, Yuen KY. Coronaviruses - drug discovery and therapeutic options. Nat Rev Drug Discov. 2016 May;15(5):327-47. Epub 2016/02/13. doi:10.1038/nrd.2015.37. Cited in: Pubmed; PMID 26868298.

4. Liu J, Zheng X, Tong Q, Li W, Wang B, Sutter K, Trilling M, Lu M, Dittmer U, Yang D. Overlapping and discrete aspects of the pathology and pathogenesis of the emerging human pathogenic coronaviruses SARS-CoV, MERS-CoV, and 2019-nCoV. J Med Virol. 2020 May;92(5):491-494. Epub 2020/02/15. doi:10.1002/jmv.25709. Cited in: Pubmed; PMID 32056249.

5. Baud D, Qi X, Nielsen-Saines K, Musso D, Pomar L, Favre G. Real estimates of mortality following COVID-19 infection. Lancet Infect Dis. 2020 Mar 12. Epub 2020/03/17. doi:10.1016/S1473-3099(20)30195-X. Cited in: Pubmed; PMID 32171390.

6. Mizumoto K, Chowell G. Estimating Risk for Death from 2019 Novel Coronavirus Disease, China, January-February 2020. Emerg Infect Dis. 2020 Mar 13;26(6). Epub 2020/03/14. doi:10.3201/eid2606.200233. Cited in: Pubmed; PMID 32168464.

7. Zhou F, Yu T, Du R, Fan G, Liu Y, Liu Z, Xiang J, Wang Y, Song B, Gu X, Guan L, Wei Y, Li H, Wu X, Xu J, Tu S, Zhang Y, Chen H, Cao B. Clinical course and risk factors for mortality of adult inpatients with COVID-19 in Wuhan, China: a retrospective cohort study. Lancet. 2020 Mar 11. Epub 2020/03/15. doi:10.1016/S0140-6736(20)30566-3. Cited in: Pubmed; PMID 32171076.

8. Hoffmann M, Kleine-Weber H, Schroeder S, Kruger N, Herrler T, Erichsen S, Schiergens TS, Herrler G, Wu NH, Nitsche A, Muller MA, Drosten C, Pohlmann S. SARS-CoV-2 Cell Entry Depends on ACE2 and TMPRSS2 and Is Blocked by a Clinically Proven Protease Inhibitor. Cell. 2020 Mar 4. Epub 2020/03/07. doi:10.1016/j.cell.2020.02.052. Cited in: Pubmed; PMID 32142651.

9. Guan WJ, Ni ZY, Hu Y, Liang WH, Ou CQ, He JX, Liu L, Shan H, Lei CL, Hui DSC, Du B, Li LJ, Zeng G, Yuen KY, Chen RC, Tang CL, Wang T, Chen PY, Xiang J, Li SY, Wang JL, Liang ZJ, Peng YX, Wei L, Liu Y, Hu YH, Peng P, Wang JM, Liu JY, Chen Z, Li G, Zheng ZJ, Qiu SQ, Luo J, Ye CJ, Zhu SY, Zhong NS, China Medical Treatment Expert Group for C. Clinical Characteristics of Coronavirus Disease 2019 in China. N Engl J Med. 2020 Feb 28. Epub 2020/02/29. doi:10.1056/NEJMoa2002032. Cited in: Pubmed; PMID 32109013.

10. Wu C, Chen X, Cai Y, Xia J, Zhou X, Xu S, Huang H, Zhang L, Zhou X, Du C, Zhang Y, Song J, Wang S, Chao Y, Yang Z, Xu J, Zhou X, Chen D, Xiong W, Xu L, Zhou F, Jiang J, Bai C, Zheng J, Song Y. Risk Factors Associated With Acute Respiratory Distress Syndrome and Death in Patients With Coronavirus Disease 2019 Pneumonia in Wuhan, China. JAMA Intern Med. 2020 Mar 13. doi:10.1001/jamainternmed.2020.0994. Cited in: Pubmed; PMID 32167524.

11. Zhang JJ, Dong X, Cao YY, Yuan YD, Yang YB, Yan YQ, Akdis CA, Gao YD. Clinical characteristics of 140 patients infected with SARS-CoV-2 in Wuhan, China. Allergy. 2020 Feb 19. Epub 2020/02/23. doi:10.1111/all.14238. Cited in: Pubmed; PMID 32077115.

12. Dong L, Hu S, Gao J. Discovering drugs to treat coronavirus disease 2019 (COVID-19). Drug Discov Ther. 2020;14(1):58-60. Epub 2020/03/10. doi:10.5582/ddt.2020.01012. Cited in: Pubmed; PMID 32147628.

13. Mehta P MD, Brown M, Sanchez E, Tattersall RS, Manson JJ, on behalf of theHLH Across Speciality Collaboration, UK. COVID-19: consider cytokine storm syndromes and immunosuppression [Correspondence]. The Lancet. 2020 March 16, 2020;online first. doi:10.1016/S0140-6736(20)30628-0.

14. Ruan Q, Yang K, Wang W, Jiang L, Song J. Clinical predictors of mortality due to COVID-19 based on an analysis of data of 150 patients from Wuhan, China. Intensive Care Med. 2020 Mar 3. Epub 2020/03/04. doi:10.1007/s00134-020-05991-x. Cited in: Pubmed; PMID 32125452.

15. Yao X, Ye F, Zhang M, Cui C, Huang B, Niu P, Liu X, Zhao L, Dong E, Song C, Zhan S, Lu R, Li H, Tan W, Liu D. In Vitro Antiviral Activity and Projection of Optimized Dosing Design of Hydroxychloroquine for the Treatment of Severe Acute Respiratory Syndrome Coronavirus 2 (SARS-CoV-2). Clin Infect Dis. 2020 Mar 9. Epub 2020/03/10. doi:10.1093/cid/ciaa237. Cited in: Pubmed; PMID 32150618.

16. Touret F, de Lamballerie X. Of chloroquine and COVID-19. Antiviral Res. 2020 Mar 5;177:104762. Epub 2020/03/10. doi:10.1016/j.antiviral.2020.104762. Cited in: Pubmed; PMID 32147496.

17. Fox RI. Mechanism of action of hydroxychloroquine as an antirheumatic drug. Semin Arthritis Rheum. 1993 Oct;23(2 Suppl 1):82-91. Epub 1993/10/01. doi:10.1016/s0049-0172(10)80012-5. Cited in: Pubmed; PMID 8278823.

18. Colson P, Rolain JM, Raoult D. Chloroquine for the 2019 novel coronavirus SARS-CoV-2. Int J Antimicrob Agents. 2020 Mar;55(3):105923. Epub 2020/02/20. doi:10.1016/j.ijantimicag.2020.105923. Cited in: Pubmed; PMID 32070753.

19. Chan KS, Lai ST, Chu CM, Tsui E, Tam CY, Wong MM, Tse MW, Que TL, Peiris JS, Sung J, Wong VC, Yuen KY. Treatment of severe acute respiratory syndrome with lopinavir/ritonavir: a multicentre retrospective matched cohort study. Hong Kong Med J. 2003 Dec;9(6):399-406. Epub 2003/12/09. Cited in: Pubmed; PMID 14660806.

20. Chu CM, Cheng VC, Hung IF, Wong MM, Chan KH, Chan KS, Kao RY, Poon LL, Wong CL, Guan Y, Peiris JS, Yuen KY, Group HUSS. Role of lopinavir/ritonavir in the treatment of SARS: initial virological and clinical findings. Thorax. 2004 Mar;59(3):252-6. Epub 2004/02/27. doi:10.1136/thorax.2003.012658. Cited in: Pubmed; PMID 14985565.

21. Chan JF, Yao Y, Yeung ML, Deng W, Bao L, Jia L, Li F, Xiao C, Gao H, Yu P, Cai JP, Chu H, Zhou J, Chen H, Qin C, Yuen KY. Treatment With Lopinavir/Ritonavir or Interferon-beta1b Improves Outcome of MERS-CoV Infection in a Nonhuman Primate Model of Common Marmoset. J Infect Dis. 2015 Dec 15;212(12):1904-13. Epub 2015/07/23. doi:10.1093/infdis/jiv392. Cited in: Pubmed; PMID 26198719.

22. Sheahan TP, Sims AC, Leist SR, Schafer A, Won J, Brown AJ, Montgomery SA, Hogg A, Babusis D, Clarke MO, Spahn JE, Bauer L, Sellers S, Porter D, Feng JY, Cihlar T, Jordan R, Denison MR, Baric RS. Comparative therapeutic efficacy of remdesivir and combination lopinavir, ritonavir, and interferon beta against MERS-CoV. Nat Commun. 2020 Jan 10;11(1):222. Epub 2020/01/12. doi:10.1038/s41467-019-13940-6. Cited in: Pubmed; PMID 31924756.

23. Yao TT, Qian JD, Zhu WY, Wang Y, Wang GQ. A systematic review of lopinavir therapy for SARS coronavirus and MERS coronavirus-A possible reference for coronavirus disease-19 treatment option. J Med Virol. 2020 Feb 27. Epub 2020/02/28. doi:10.1002/jmv.25729. Cited in: Pubmed; PMID 32104907.

24. Roberts DM, Ray JE, Buckley NA. Mild clinical toxicity and dose-dependent pharmacokinetics following acute lopinavir/ritonavir poisoning in a HIV-positive patient. AIDS. 2008 Mar 30;22(6):792-3. Epub 2008/03/22. doi:10.1097/QAD.0b013e3282f4a0dd. Cited in: Pubmed; PMID 18356614.

25. Croxtall JD, Perry CM. Lopinavir/Ritonavir: a review of its use in the management of HIV-1 infection. Drugs. 2010 Oct 1;70(14):1885-915. Epub 2010/09/15. doi:10.2165/11204950-000000000-00000. Cited in: Pubmed; PMID 20836579.

26. Wang M, Cao R, Zhang L, Yang X, Liu J, Xu M, Shi Z, Hu Z, Zhong W, Xiao G. Remdesivir and chloroquine effectively inhibit the recently emerged novel coronavirus (2019-nCoV) in vitro. Cell Res. 2020 Mar;30(3):269-271. Epub 2020/02/06. doi:10.1038/s41422-020-0282-0. Cited in: Pubmed; PMID 32020029.

27. Beigel JH, Tomashek KM, Dodd LE, Mehta AK, Zingman BS, Kalil AC, Hohmann E, Chu HY, Luetkemeyer A, Kline S, Lopez de Castilla D, Finberg RW, Dierberg K, Tapson V, Hsieh L, Patterson TF, Paredes R, Sweeney DA, Short WR, Touloumi G, Lye DC, Ohmagari N, Oh MD, Ruiz-Palacios GM, Benfield T, Fatkenheuer G, Kortepeter MG, Atmar RL, Creech CB, Lundgren J, Babiker AG, Pett S, Neaton JD, Burgess TH, Bonnett T, Green M, Makowski M, Osinusi A, Nayak S, Lane HC, Members A-SG. Remdesivir for the Treatment of Covid-19 - Final Report. N Engl J Med. 2020 Oct 8. Epub 2020/05/24. doi:10.1056/NEJMoa2007764. Cited in: Pubmed; PMID 32445440.

28. Spinner CD, Gottlieb RL, Criner GJ, Arribas Lopez JR, Cattelan AM, Soriano Viladomiu A, Ogbuagu O, Malhotra P, Mullane KM, Castagna A, Chai LYA, Roestenberg M, Tsang OTY, Bernasconi E, Le Turnier P, Chang SC, SenGupta D, Hyland RH, Osinusi AO, Cao H, Blair C, Wang H, Gaggar A, Brainard DM, McPhail MJ, Bhagani S, Ahn MY, Sanyal AJ, Huhn G, Marty FM, Investigators G-U-. Effect of Remdesivir vs Standard Care on Clinical Status at 11 Days in Patients With Moderate COVID-19: A Randomized Clinical Trial. JAMA. 2020 Sep 15;324(11):1048-1057. Epub 2020/08/22. doi:10.1001/jama.2020.16349. Cited in: Pubmed; PMID 32821939.

29. Wang Y, Zhang D, Du G, Du R, Zhao J, Jin Y, Fu S, Gao L, Cheng Z, Lu Q, Hu Y, Luo G, Wang K, Lu Y, Li H, Wang S, Ruan S, Yang C, Mei C, Wang Y, Ding D, Wu F, Tang X, Ye X, Ye Y, Liu B, Yang J, Yin W, Wang A, Fan G, Zhou F, Liu Z, Gu X, Xu J, Shang L, Zhang Y, Cao L, Guo T, Wan Y, Qin H, Jiang Y, Jaki T, Hayden FG, Horby PW, Cao B, Wang C. Remdesivir in adults with severe COVID-19: a randomised, double-blind, placebo-controlled, multicentre trial. Lancet. 2020 May 16;395(10236):1569-1578. Epub 2020/05/20. doi:10.1016/S0140-6736(20)31022-9. Cited in: Pubmed; PMID 32423584.

30. Han W, Quan B, Guo Y, Zhang J, Lu Y, Feng G, Wu Q, Fang F, Cheng L, Jiao N, Li X, Chen Q. The course of clinical diagnosis and treatment of a case infected with coronavirus disease 2019. J Med Virol. 2020 May;92(5):461-463. Epub 2020/02/20. doi:10.1002/jmv.25711. Cited in: Pubmed; PMID 32073161.

31. Tang N, Li D, Wang X, Sun Z. Abnormal coagulation parameters are associated with poor prognosis in patients with novel coronavirus pneumonia. J Thromb Haemost. 2020 Feb 19. Epub 2020/02/20. doi:10.1111/jth.14768. Cited in: Pubmed; PMID 32073213.

32. Shi M, Wang L, Zhou J, Ji S, Wang N, Tong L, Bi J, Song Y, Hu J, Chen X. Direct factor Xa inhibition attenuates acute lung injury progression via modulation of the PAR-2/NF-kappaB signaling pathway. Am J Transl Res. 2018;10(8):2335-2349. Epub 2018/09/14. Cited in: Pubmed; PMID 30210674.

33. Fang L, Karakiulakis G, Roth M. Are patients with hypertension and diabetes mellitus at increased risk for COVID-19 infection? Lancet Respir Med. 2020 Mar 11. Epub 2020/03/15. doi:10.1016/S2213-2600(20)30116-8. Cited in: Pubmed; PMID 32171062.

34. Li XC, Zhang J, Zhuo JL. The vasoprotective axes of the renin-angiotensin system: Physiological relevance and therapeutic implications in cardiovascular, hypertensive and kidney diseases. Pharmacol Res. 2017 Nov;125(Pt A):21-38. doi:10.1016/j.phrs.2017.06.005. Cited in: Pubmed; PMID 28619367.

35. Wan Y, Shang J, Graham R, Baric RS, Li F. Receptor recognition by novel coronavirus from Wuhan: An analysis based on decade-long structural studies of SARS. J Virol. 2020 Jan 29. doi:10.1128/JVI.00127-20. Cited in: Pubmed; PMID 31996437.

36. Grisoni Colli A. [Early diagnosis of cerebral palsy in children]. Arch Ital Pediatr Pueric. 1967 Jan-Feb;25(1):1-10. Diagnosi precoce di paralisi cerebrale infantile. Epub 1967/01/01. Cited in: Pubmed; PMID 5607101.

37. G dS. Position Statement of the ESC Council on Hypertension on ACE-Inhibitors and Angiotensin Receptor Blockers. 2020.

38. Wang D, Chai XQ, Magnussen CG, Zosky GR, Shu SH, Wei X, Hu SS. Renin-angiotensin-system, a potential pharmacological candidate, in acute respiratory distress syndrome during mechanical ventilation. Pulm Pharmacol Ther. 2019 Oct;58:101833. Epub 2019/08/04. doi:10.1016/j.pupt.2019.101833. Cited in: Pubmed; PMID 31376462.

39. Poglitsch M, Domenig O, Schwager C, Stranner S, Peball B, Janzek E, Wagner B, Jungwirth H, Loibner H, Schuster M. Recombinant Expression and Characterization of Human and Murine ACE2: Species-Specific Activation of the Alternative Renin-Angiotensin-System. Int J Hypertens. 2012;2012:428950. Epub 2012/04/21. doi:10.1155/2012/428950. Cited in: Pubmed; PMID 22518284.

40. Haber PK, Ye M, Wysocki J, Maier C, Haque SK, Batlle D. Angiotensin-converting enzyme 2-independent action of presumed angiotensin-converting enzyme 2 activators: studies in vivo, ex vivo, and in vitro. Hypertension. 2014 Apr;63(4):774-82. Epub 2014/01/22. doi:10.1161/HYPERTENSIONAHA.113.02856. Cited in: Pubmed; PMID 24446061.

41. Ye M, Wysocki J, Gonzalez-Pacheco FR, Salem M, Evora K, Garcia-Halpin L, Poglitsch M, Schuster M, Batlle D. Murine recombinant angiotensin-converting enzyme 2: effect on angiotensin II-dependent hypertension and distinctive angiotensin-converting enzyme 2 inhibitor characteristics on rodent and

human angiotensin-converting enzyme 2. Hypertension. 2012 Sep;60(3):730-40. Epub 2012/07/11. doi:10.1161/HYPERTENSIONAHA.112.198622. Cited in: Pubmed; PMID 22777933.

42. Tuettenberg J, Seiz M, Debatin KM, Hollburg W, von Staden M, Thiemann M, Hareng B, Fricke H, Kunz C. Pharmacokinetics, pharmacodynamics, safety and tolerability of APG101, a CD95-Fc fusion protein, in healthy volunteers and two glioma patients [Clinical Trial, Phase I
Randomized Controlled Trial]. Int Immunopharmacol. 2012;13(1):93-100.

43. Dhein J, Walczak H, Baumler C, Debatin KM, Krammer PH. Autocrine T-cell suicide mediated by APO-1/(Fas/CD95). Nature. 1995;373(6513):438-41.

44. Zheng M, Gao Y, Wang G, Song G, Liu S, Sun D, Xu Y, Tian Z. Functional exhaustion of antiviral lymphocytes in COVID-19 patients. Cell Mol Immunol. 2020;19(10):020-0402.

45. Tan L, Wang Q, Zhang D, Ding J, Huang Q, Tang Y-Q, Wang Q, Miao H. Lymphopenia predicts disease severity of COVID-19: a descriptive and predictive study. medRxiv. 2020:2020.03.01.20029074. doi:10.1101/2020.03.01.20029074.

46. Xu T, Qiao J, Zhao L, Wang G, He G, Li K, Tian Y, Gao M, Wang J, Wang H, Dong C. Acute respiratory distress syndrome induced by avian influenza A (H5N1) virus in mice [Research Support, Non-U S Gov't]. Am J Respir Crit Care Med. 2006;174(9):1011-7.

47. Hogner K, Wolff T, Pleschka S, Plog S, Gruber AD, Kalinke U, Walmrath HD, Bodner J, Gattenlohner S, Lewe-Schlosser P, Matrosovich M, Seeger W, Lohmeyer J, Herold S. Macrophage-expressed IFN-beta contributes to apoptotic alveolar epithelial cell injury in severe influenza virus pneumonia [Research Support, Non-U S Gov't]. PLoS Pathog. 2013;9(2):28.

48. Davidson S, Crotta S, McCabe TM, Wack A. Pathogenic potential of interferon alphabeta in acute influenza infection [Research Support, Non-U S Gov't]. Nat Commun. 2014;5(3864).

49. Herold S, Ludwig S, Pleschka S, Wolff T. Apoptosis signaling in influenza virus propagation, innate host defense, and lung injury [Research Support, Non-U S Gov't
Review]. J Leukoc Biol. 2012;92(1):75-82.

50. Lopez AD, Avasarala S, Grewal S, Murali AK, London L. Differential role of the Fas/Fas ligand apoptotic pathway in inflammation and lung fibrosis associated with reovirus 1/L-induced bronchiolitis obliterans organizing pneumonia and acute respiratory distress syndrome [Comparative Study
Research Support, N I H , Extramural
Research Support, Non-U S Gov't]. J Immunol. 2009;183(12):8244-57.

51. Fujikura D, Chiba S, Muramatsu D, Kazumata M, Nakayama Y, Kawai T, Akira S, Kida H, Miyazaki T. Type-I interferon is critical for FasL expression on lung cells to determine the severity of influenza [Research Support, Non-U S Gov't]. PLoS One. 2013;8(2):8.

52. Peteranderl C, Morales-Nebreda L, Selvakumar B, Lecuona E, Vadasz I, Morty RE, Schmoldt C, Bespalowa J, Wolff T, Pleschka S, Mayer K, Gattenloehner S, Fink L, Lohmeyer J, Seeger W, Sznajder JI, Mutlu GM, Budinger GR, Herold S. Macrophage-epithelial paracrine crosstalk inhibits lung edema clearance during influenza infection [Research Support, N I H , Extramural]. J Clin Invest. 2016;126(4):1566-80.

53. Wick W, Fricke H, Junge K, Kobyakov G, Martens T, Heese O, Wiestler B, Schliesser MG, von Deimling A, Pichler J, Vetlova E, Harting I, Debus J, Hartmann C, Kunz C, Platten M, Bendszus M, Combs SE. A phase II, randomized, study of weekly APG101+reirradiation versus reirradiation in progressive glioblastoma [Clinical Trial, Phase II
Multicenter Study
Randomized Controlled Trial
Research Support, Non-U S Gov't]. Clin Cancer Res. 2014;20(24):6304-13.

54. Ullah W, H MA, Roomi S, Sattar Y, Almas T, Narayana Gowda S, Saeed R, Mukhtar M, Ahmad A, Oliver T, Alraies MC, Haas DC, Fischman DL. Safety and Efficacy of Hydroxychloroquine in COVID-19: A Systematic Review and Meta-Analysis. J Clin Med Res. 2020 Aug;12(8):483-491. Epub 2020/08/28. doi:10.14740/jocmr4233. Cited in: Pubmed; PMID 32849936.

55. Group RC. Lopinavir-ritonavir in patients admitted to hospital with COVID-19 (RECOVERY): a randomised, controlled, open-label, platform trial. Lancet. 2020 Oct 5. Epub 2020/10/09. doi:10.1016/S0140-6736(20)32013-4. Cited in: Pubmed; PMID 33031764.

56. Group RC, Horby P, Lim WS, Emberson JR, Mafham M, Bell JL, Linsell L, Staplin N, Brightling C, Ustianowski A, Elmahi E, Prudon B, Green C, Felton T, Chadwick D, Rege K, Fegan C, Chappell LC, Faust SN, Jaki T, Jeffery K, Montgomery A, Rowan K, Juszczak E, Baillie JK, Haynes R, Landray MJ. Dexamethasone in Hospitalized Patients with Covid-19 - Preliminary Report. N Engl J Med. 2020 Jul 17. Epub 2020/07/18. doi:10.1056/NEJMoa2021436. Cited in: Pubmed; PMID 32678530.

57. Imai Y, Kuba K, Rao S, Huan Y, Guo F, Guan B, Yang P, Sarao R, Wada T, Leong-Poi H, Crackower MA, Fukamizu A, Hui CC, Hein L, Uhlig S, Slutsky AS, Jiang C, Penninger JM. Angiotensin-converting enzyme 2 protects from severe acute lung failure. Nature. 2005 Jul 7;436(7047):112-6. Epub 2005/07/08. doi:10.1038/nature03712. Cited in: Pubmed; PMID 16001071.

58. Sauler M, Bazan IS, Lee PJ. Cell Death in the Lung: The Apoptosis-Necroptosis Axis [Research Support, Non-U S Gov't
Research Support, N I H , Extramural
Research Support, U S Gov't, Non-P H S]. Annu Rev Physiol. 2019;81:375-402.

59. Matute-Bello G, Liles WC, Steinberg KP, Kiener PA, Mongovin S, Chi EY, Jonas M, Martin TR. Soluble Fas ligand induces epithelial cell apoptosis in humans with acute lung injury (ARDS) [Research Support, Non-U S Gov't
Research Support, U S Gov't, Non-P H S
Research Support, U S Gov't, P H S]. J Immunol. 1999;163(4):2217-25.

60. Kitamura Y, Hashimoto S, Mizuta N, Kobayashi A, Kooguchi K, Fujiwara I, Nakajima H. Fas/FasL-dependent apoptosis of alveolar cells after lipopolysaccharide-induced lung injury in mice [Research Support, Non-U S Gov't]. Am J Respir Crit Care Med. 2001;163(3 Pt 1):762-9.

61. Kuwano K, Hagimoto N, Kawasaki M, Yatomi T, Nakamura N, Nagata S, Suda T, Kunitake R, Maeyama T, Miyazaki H, Hara N. Essential roles of the Fas-Fas ligand pathway in the development of pulmonary fibrosis. J Clin Invest. 1999;104(1):13-9.

62. Albertine KH, Soulier MF, Wang Z, Ishizaka A, Hashimoto S, Zimmerman GA, Matthay MA, Ware LB. Fas and fas ligand are up-regulated in pulmonary edema fluid and lung tissue of patients with acute lung injury and the acute respiratory distress syndrome [Comparative Study
Research Support, U S Gov't, P H S]. Am J Pathol. 2002;161(5):1783-96.

63. Hagimoto N, Kuwano K, Miyazaki H, Kunitake R, Fujita M, Kawasaki M, Kaneko Y, Hara N. Induction of apoptosis and pulmonary fibrosis in mice in response to ligation of Fas antigen. American journal of respiratory cell and molecular biology. 1997;17(3):272-278.

64. Matute-Bello G, Liles WC, Frevert CW, Nakamura M, Ballman K, Vathanaprida C, Kiener PA, Martin TR. Recombinant human Fas ligand induces alveolar epithelial cell apoptosis and lung injury in rabbits. American Journal of Physiology-Lung Cellular and Molecular Physiology. 2001;281(2):L328-L335.

65. McCormick S, Shaler CR, Small CL, Horvath C, Damjanovic D, Brown EG, Aoki N, Takai T, Xing Z. Control of pathogenic CD4 T cells and lethal immunopathology by signaling immunoadaptor DAP12 during influenza infection. J Immunol. 2011;187(8):4280-92.

66. Matute-Bello G, Frevert CW, Liles WC, Nakamura M, Ruzinski JT, Ballman K, Wong VA, Vathanaprida C, Martin TR. Fas/Fas ligand system mediates epithelial injury, but not pulmonary host defenses, in response to inhaled bacteria [Research Support, Non-U S Gov't
Research Support, U S Gov't, P H S]. Infect Immun. 2001;69(9):5768-76.

67. Matute-Bello G, Winn RK, Martin TR, Liles WC. Sustained lipopolysaccharide-induced lung inflammation in mice is attenuated by functional deficiency of the Fas/Fas ligand system [Research Support, U S Gov't, Non-P H S
Research Support, U S Gov't, P H S]. Clin Diagn Lab Immunol. 2004;11(2):358-61.

68. Perl M, Chung CS, Perl U, Lomas-Neira J, de Paepe M, Cioffi WG, Ayala A. Fas-induced pulmonary apoptosis and inflammation during indirect acute lung injury [Research Support, N I H , Extramural Research Support, Non-U S Gov't]. Am J Respir Crit Care Med. 2007;176(6):591-601.

69. Kong Q, Wu X, Duan W, Zhan L, Song X. Penehyclidine hydrochloride exerts protective effects in rats with acute lung injury via the Fas/FasL signaling pathway. Exp Ther Med. 2019;17(5):3598-3606.

70. Rodrigue-Gervais IG, Labbe K, Dagenais M, Dupaul-Chicoine J, Champagne C, Morizot A, Skeldon A, Brincks EL, Vidal SM, Griffith TS, Saleh M. Cellular inhibitor of apoptosis protein cIAP2 protects against

pulmonary tissue necrosis during influenza virus infection to promote host survival [Research Support, N I H , Extramural
Research Support, Non-U S Gov't]. Cell Host Microbe. 2014;15(1):23-35.

71. Matute-Bello G, Liles WC, Frevert CW, Dhanireddy S, Ballman K, Wong V, Green RR, Song HY, Witcher DR, Jakubowski JA, Martin TR. Blockade of the Fas/FasL system improves pneumococcal clearance from the lungs without preventing dissemination of bacteria to the spleen [Research Support, Non-U S Gov't
Research Support, U S Gov't, Non-P H S
Research Support, U S Gov't, P H S]. J Infect Dis. 2005;191(4):596-606.

72. Rodriguez A, Rello J, Neira J, Maskin B, Ceraso D, Vasta L, Palizas F. Effects of high-dose of intravenous immunoglobulin and antibiotics on survival for severe sepsis undergoing surgery. Shock. 2005 Apr;23(4):298-304. Epub 2005/04/02. doi:10.1097/01.shk.0000157302.69125.f8. Cited in: Pubmed; PMID 15803051.

73. Kreymann KG, de Heer G, Nierhaus A, Kluge S. Use of polyclonal immunoglobulins as adjunctive therapy for sepsis or septic shock. Crit Care Med. 2007 Dec;35(12):2677-85. Epub 2007/12/13. Cited in: Pubmed; PMID 18074464.

74. WHO.

75. Cohen AT, Spiro TE, Buller HR, Haskell L, Hu D, Hull R, Mebazaa A, Merli G, Schellong S, Spyropoulos AC, Tapson V, Investigators M. Rivaroxaban for thromboprophylaxis in acutely ill medical patients. N Engl J Med. 2013 Feb 7;368(6):513-23. Epub 2013/02/08. doi:10.1056/NEJMoa1111096. Cited in: Pubmed; PMID 23388003.

76. Attoquant.

77. Rice GI, Thomas DA, Grant PJ, Turner AJ, Hooper NM. Evaluation of angiotensin-converting enzyme (ACE), its homologue ACE2 and neprilysin in angiotensin peptide metabolism. Biochem J. 2004 Oct 1;383(Pt 1):45-51. Epub 2004/07/31. doi:10.1042/BJ20040634. Cited in: Pubmed; PMID 15283675.

78. Valent P, Orazi A, Steensma DP, Ebert BL, Haase D, Malcovati L, van de Loosdrecht AA, Haferlach T, Westers TM, Wells DA. Proposed minimal diagnostic criteria for myelodysplastic syndromes (MDS) and potential pre-MDS conditions. Oncotarget. 2017;8(43):73483.

79. Bauer P, Bretz F, Dragalin V, Konig F, Wassmer G. Twenty-five years of confirmatory adaptive designs: opportunities and pitfalls. Stat Med. 2016 Feb 10;35(3):325-47. Epub 2015/03/18. doi:10.1002/sim.6472. Cited in: Pubmed; PMID 25778935.

80. Lehmacher W, Wassmer G. Adaptive sample size calculations in group sequential trials. Biometrics. 1999 Dec;55(4):1286-90. Epub 2001/04/21. doi:10.1111/j.0006-341x.1999.01286.x. Cited in: Pubmed; PMID 11315085.

81. Magirr D, Jaki T, Koenig F, Posch M. Sample Size Reassessment and Hypothesis Testing in Adaptive Survival Trials. PLoS One. 2016;11(2):e0146465. Epub 2016/02/11. doi:10.1371/journal.pone.0146465. Cited in: Pubmed; PMID 26863139.

82. Hodgson LE, Dimitrov BD, Congleton J, Venn R, Forni LG, Roderick PJ. A validation of the National Early Warning Score to predict outcome in patients with COPD exacerbation. Thorax. 2017 Jan;72(1):23-30. Epub 2016/08/25. doi:10.1136/thoraxjnl-2016-208436. Cited in: Pubmed; PMID 27553223.

15 Appendix I National Early Warning Score (NEWS)

**Physiological Parameters**	**3**	**2**	**1**	**0**	**1**	**2**	**3**
**Respiration rate**	≤8		9-11	12-20		21-24	≥25
**Oxygen saturation**	≤91	92-93	94-95	≥96			
**Any supplemental oxygen**		yes		no			
**Temperature**	≤35		35.1-36	36.1-38	38.1-39	≥39	
**Systolic BP**	≤90	91-100	101-110	111-219			≥220
**Heart rate**	≤40		41-50	51-90	91-110	111-130	≥131
**Level of consciousness**				A			V, P, or U

National Early Warning Score (NEWS) (82), A = Alert, V = verbal response or voice, P = painful stimuli or pain, U = unresponsive
